# Late-Stage Functionalization of Living Organisms: Rethinking Selectivity in Biology

**DOI:** 10.1021/acs.chemrev.3c00579

**Published:** 2024-01-17

**Authors:** Andrew M. Giltrap, Yizhi Yuan, Benjamin G. Davis

**Affiliations:** † The Rosalind Franklin Institute, Oxfordshire OX11 0FA, U.K.; ‡ Department of Pharmacology, University of Oxford, Oxford OX1 3QT, U.K.

## Abstract

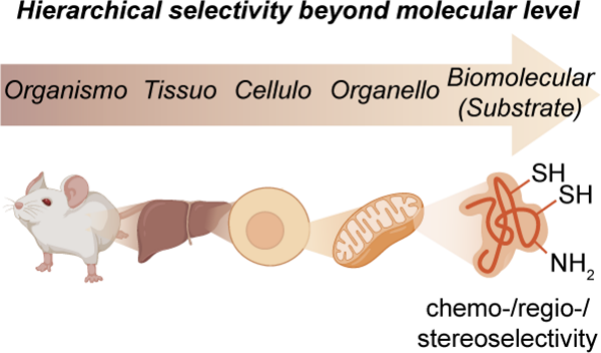

With unlimited selectivity, full post-translational chemical control of biology would circumvent the dogma of genetic control. The resulting direct manipulation of organisms would enable atomic-level precision in “editing” of function. We argue that a key aspect that is still missing in our ability to do this (at least with a high degree of control) is the selectivity of a given chemical reaction in a living organism. In this Review, we systematize existing illustrative examples of chemical selectivity, as well as identify needed chemical selectivities set in a hierarchy of anatomical complexity: organismo- (selectivity for a given organism over another), tissuo- (selectivity for a given tissue type in a living organism), cellulo- (selectivity for a given cell type in an organism or tissue), and organelloselectivity (selectivity for a given organelle or discrete body within a cell). Finally, we analyze more traditional concepts such as regio-, chemo-, and stereoselective reactions where additionally appropriate. This survey of late-stage biomolecule methods emphasizes, where possible, functional consequences (*i.e.*, biological function). In this way, we explore a concept of late-stage functionalization of living organisms (where “late” is taken to mean at a given state of an organism in time) in which programmed and selective chemical reactions take place in life. By building on precisely analyzed notions (*e.g.*, mechanism and selectivity) we believe that the logic of chemical methodology might ultimately be applied to increasingly complex molecular constructs in biology. This could allow principles developed at the simple, small-molecule level to progress hierarchically even to manipulation of physiology.

## 1 Introduction

The functional manipulation of biology is at one, reductionist, level a question of chemical control with the potential for precision afforded by atomic-level alteration. The implementation of such manipulation therefore becomes a challenge, fundamentally in chemical selectivity, that will allow the correct localization and identity of these changes through, typically, altered covalency (and thus covalent bond breaking and making). Such selectivity will be the major focus of this Review. In biology, the strategic timing of such alterations makes a profound difference. Current strategies are divided in this timing. While it may be argued that more traditional pharmacological notions focus on related chemical changes at a late stage that are relevant to function in the immediate (*i.e.*, “in the present”) of a given organism, more recent postgenomic methods (*e.g.*, “gene editing” and mRNA delivery) have instead exploited semipredictable pathways of “sequential information”^1^ to change later organismal function in a less direct yet programmable way. Although seemingly conceptually distinct, we therefore posit that useful parallels may be drawn between the design of synthetic chemical pathways and the implementation of such biological alteration. In this way, by building on precisely analyzed notions of, for example, mechanism and selectivity, the logic of chemical methodology might ultimately be applied to increasingly complex molecular constructs. While noting the danger of oversimplification, this could allow principles developed at the simple, small-molecule level to progress hierarchically even to manipulation of physiology. It is clearly trite to consider organisms to *only* be far-from-equilibrium supramolecular assemblies, but we nonetheless believe that the principles that might emerge through this form of chemical analysis will help to dissect challenges in a useful and addressable manner and at the same time maintain relevance to biological and physiological function.

We realize too that the concept of “late” timing becomes potentially multifaceted in our analyses. Here we explicitly take this to mean a given state of an organism in time. This is necessarily arbitrary but serves the purpose of largely discounting processes where the levers of selectivity are temporally more remote, such as those that require time for the transfer of sequential information to take effect. Dynamics in living organisms is a vast and very relevant subject^2^ and the role of even simple underlying chemical kinetics well-noted;^3^ we essentially side-step these important issues in our Review. This means that some powerful endogenous selectivities in physiology (*e.g.*, transcriptional regulation) will not be recapitulated or examined. Instead, we will focus on molecules that are more immediate workhorses of biology and thus largely post-translational.

**Figure 1:**
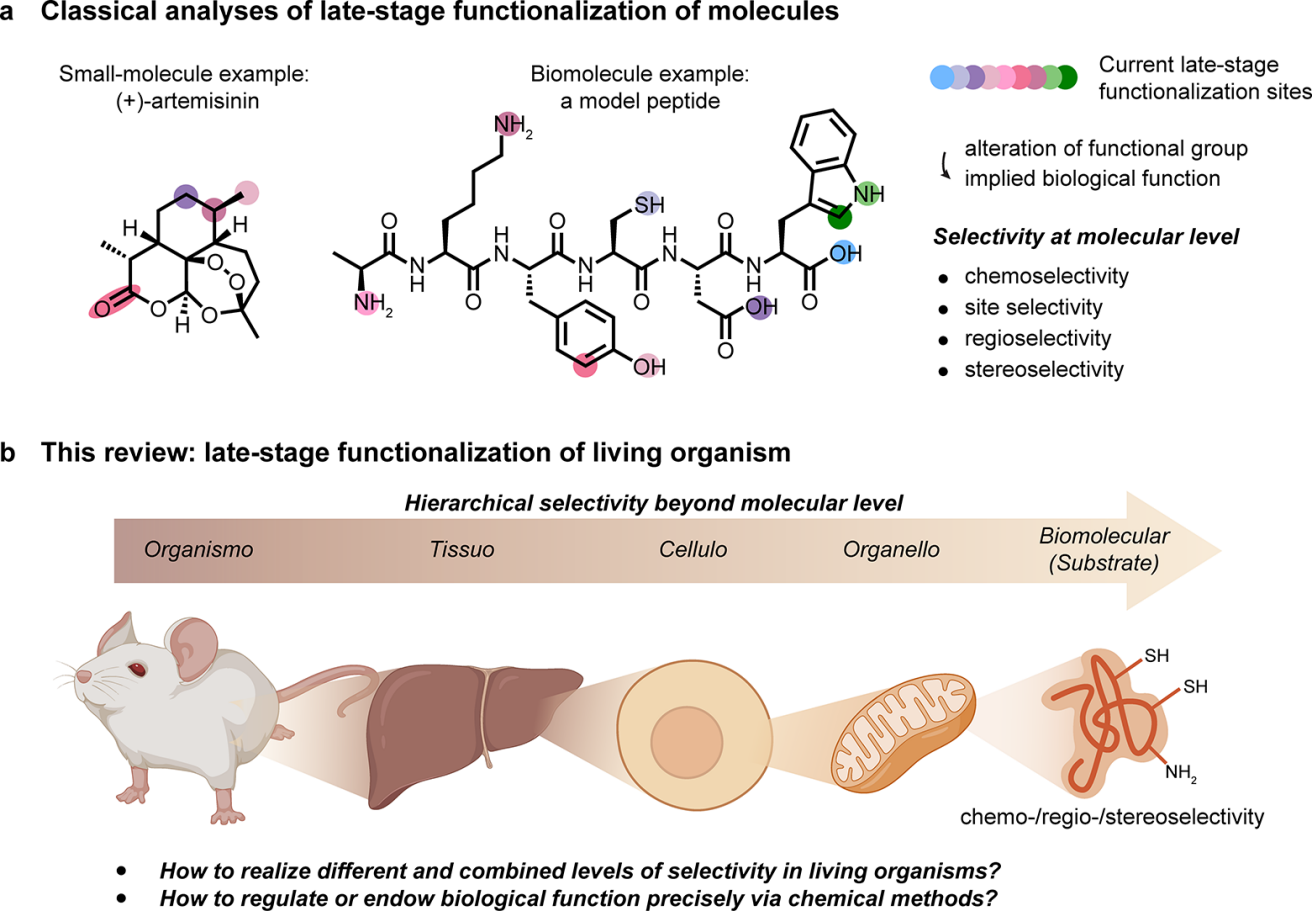
Considering organisms as a target for late stage functionalization (LSF). (a) Classical consideration of approaching small molecules for “LSF” and the associated needed modes of selectivity in chemical synthesis. (b) A suggested use of the LSF strategy for living organisms. How might one achieve layered levels of selectivity from the organismal to biomolecular that would then complement (“sit on top of”) the classical?

The concept of late-stage functionalization (LSF) is typically applied to small-molecule systems in which a “reactive handle” is present or installed into an advanced intermediate, which can then be selectively reacted under a given manifold to generate a large degree of diversity and/or generate a large compound library^4−6^ [Figure 1a]. The nature of the site of modification has been increasingly extended to encompass a notion of sites, such as C–H, that have not traditionally been viewed as reactive.^7^ Yet, the concept of function—the F in LSF—has perhaps been diluted in this notion, and for biomolecules this is perhaps paramount. Here, we focus on a survey of late-stage biomolecule methods by placing functional consequences as a critical filter. In this way, we aim to explore a concept of late-stage functionalization of living organisms in which programmed and selective chemical reactions take place in life. This research area has of course been aided by advances in what has become known as bioorthogonal chemistry (*e.g.*, “click” reactions), the topic of the 2022 Nobel Prize in Chemistry and for which there are already many thorough and excellent reviews.^8−11^ These provide a good starting point in moving toward a chemical manipulation of biology, that is, not just the genetic manipulation of biological systems but the exploitation of chemical reactivity in living organisms. However, to date, many methods focus simply on the observation (and sometimes retrieval) of the compounds of biology rather than the ability to regulate or endow function. A key aspect that is still missing in our ability to do this (at least with a high degree of control) is the *selectivity* of a given chemical reaction in a living organism. While bioorthogonal chemistry has greatly addressed the issue of functional group selectivity/chemoselectivity in biology, there are many further aspects of selectivity, as well as those of functional recapitulation, that remain elusive.

Here we will take a hierarchical viewpoint for a potentially more targeted approach in which we consider how selectivity can be imparted during the late-stage functionalization of living organisms. We plan to analyze this topic through the lens of selectivity moving down a hierarchy of anatomical complexity, in this case starting from selectivities that we refer to as organismoselectivity (selectivity for a given organism over another), tissuoselectivity (selectivity for a given tissue type in a living organism), celluloselectivity (selectivity for a given cell type in an organism or tissue), organelloselectivity (selectivity for a given organelle or discrete body within a cell), and biomolecular selectivity (selectivity for a specific biomolecule or substrate within a cell) and finally analyzing more traditional concepts such as regio-, chemo-, and stereoselective reactions where additionally appropriate [Figure 1b]. While the layers of selectivity are inherently interdependent, in this Review we partition examples based on the “highest” (in terms of organismal complexity) level of selectivity (and so are discussed in order). We note the long-standing arguments on correct phrasing (*in cellulo* vs *in cellulis*, *etc.*(12)) and do not pretend that such terms are well-founded in their etymology.

We also do not intend to give a comprehensive review of every reaction that has been used to chemically modify an organism in some way, and these have been considered, at least in part, elsewhere.^13,14^ Rather, we consider how selectivity for different aspects of a living system can be achieved and so, hopefully, provoke further research into methods to improve the precise chemical manipulation of life.

Our focus on biological (even endogenous) function means that we also therefore discount so-called “click-to-release” and other related prodrug-type strategies.^15−19^ While the fields of drug delivery and prodrug utility share some potentially similar concepts with regard to compatibility and utility of chemistry inside living systems, we consider them to be a fundamentally different approach. The control of biological function that we study here is different from the use or exploration of an organism as a vessel, albeit some examples include the generation of useful therapeutics in that vessel.

## 2 Organismoselectivity

The concept of organismal selectivity considers the ability to carry out specific chemical modification of one organism in the presence of (or even inside) another. This may seem obtuse in the context of chemical reactivity, but it has of course been routinely exploited in a more crude fashion for decades in the context of some anti-infective agents. Not all anti-infective agents result in covalent modification of e.g., target bacteria, but the desired selective targeting of a pathogenic organism inside a mammal (human) by exploiting different biochemical pathways and thus biomolecules that the different organisms utilize, is illustrative. As such, one may also consider this to be species selectivity in that a given species can be targeted over another.

Classical examples illustrate the case in point. Penicillins inhibit the biosynthesis of bacterial cell walls with an apparently minimal interaction with the host system. This selectivity comes in part via the mimicry of penicillin for the d-Ala-d-Ala dipeptide^20^ present in bacterial cell wall precursor lipid II; this covalently inhibits key transpeptidase enzymes responsible for peptidoglycan cross-linking and cell-wall maturation, a process which is absent in mammalian biology. Similarly, quinine has the ability to selectively cause the death of the malarial parasite plasmodium in mammals by exploiting a typical and classical drug mode of selective competitive inhibition. While quinine was the first antimalarial discovered (isolated in 1820) and a long-standing “drug”, a molecular target—purine nucleoside phosphorylase (PNP), an enzyme responsible for the salvage and recycling of purines—has only more recently been proposed.^21^ Quinine’s inhibition of *Plasmodium falciparum* PNP (*K*_i_ ∼ 140 nM) but lack of interaction with the endogenous human homologue is one of the most familiar forms of selectivity, namely, biomolecule selectivity, a mode in essence of host–guest chemistry (see section 6). Yet, as for many drugs, it simultaneously exploits both tissue and cellular selectivity, as it is able to inhibit its target enzyme *Pf*PNP in the pathogenic plasmodium that itself infects red blood cells in circulation, thereby highlighting the need for synergistic consideration of combined layers of selectivity that we hope to draw out in this Review. Despite this, quinine actually has a relatively low therapeutic index, with off-target toxic effects that manifest in a clinical indication commonly known as cinchonism, highlighting the need even in archetypal systems for improvement. In this way, we postulate that largely familiar pharmacological notions may start to influence strategic synthetic thinking in *in vivo* chemistry.

Thus, while there are many other examples of species-selective targeting that rely on exploitable biomolecular differences, the concept of selective late-stage functionalization of a given living organism within another remains a rare, yet extremely interesting concept. Indeed, while a number of examples are almost able to achieve this goal, or parts thereof, the true late-stage functionalization of a given organism within another remains elusive.^22^

Selective bacteria–bacteria conjugation can be considered inside living mice as an illustration of useful compatibility. A system utilizing two prefunctionalized bacterial populations, one displaying cell-surface azides (via cell-wall metabolic labeling with azido-d-Ala) and the other cell-surface cyclooctynes (via simple *N*-hydroxysuccinimide (NHS) ester-mediated cell-surface labeling) allowed cross-linking via a strain-promoted azide–alkyne cycloaddition (SPAAC) reaction *in vitro* and in a living mouse. Here selectivity is essentially driven by chemoselectivity alone, yet apparently good compatibility is shown. This is thus a case where no inherent organismal selectivity is exploited and the living system (mouse) is used essentially as a “reaction bag”. Nonetheless, this example is illustrative in two ways: first, function, as use of DBCO-displaying *Clostridium butyricum* enhanced colonization and so allowed intriguing control of function in a colitis model;^23^ second, future potential when combined with other modes of selectivity, as organism-selective incorporation and display of azide in bacteria can be achieved via differential biosynthetic pathways for metabolic incorporation between mammals and prokaryotes. 8-Azido-Kdo (3-deoxy-d-manno-octulosonic acid) can thus be incorporated into the lipopolysaccharide (LPS) of the outer cell wall of Gram negative bacteria (but not Gram positive bacteria or mammals), as shown by gavage removal of gut microbiota and *ex vivo* copper-catalyzed AAC (CuAAC) with a fluorescent alkyne.^24^ In this way, although not shown in these examples, combined “layering” of selectivities could in principle yield elegant *in vivo* function, that is, specific metabolic labeling of a given (sub)population such as Gram negative bacteria in the presence of Gram positive bacteria (the latter study)^24^ coupled with demonstrated chemoselective compatibility inside a living mammal (the former).^23^

**Figure 2:**
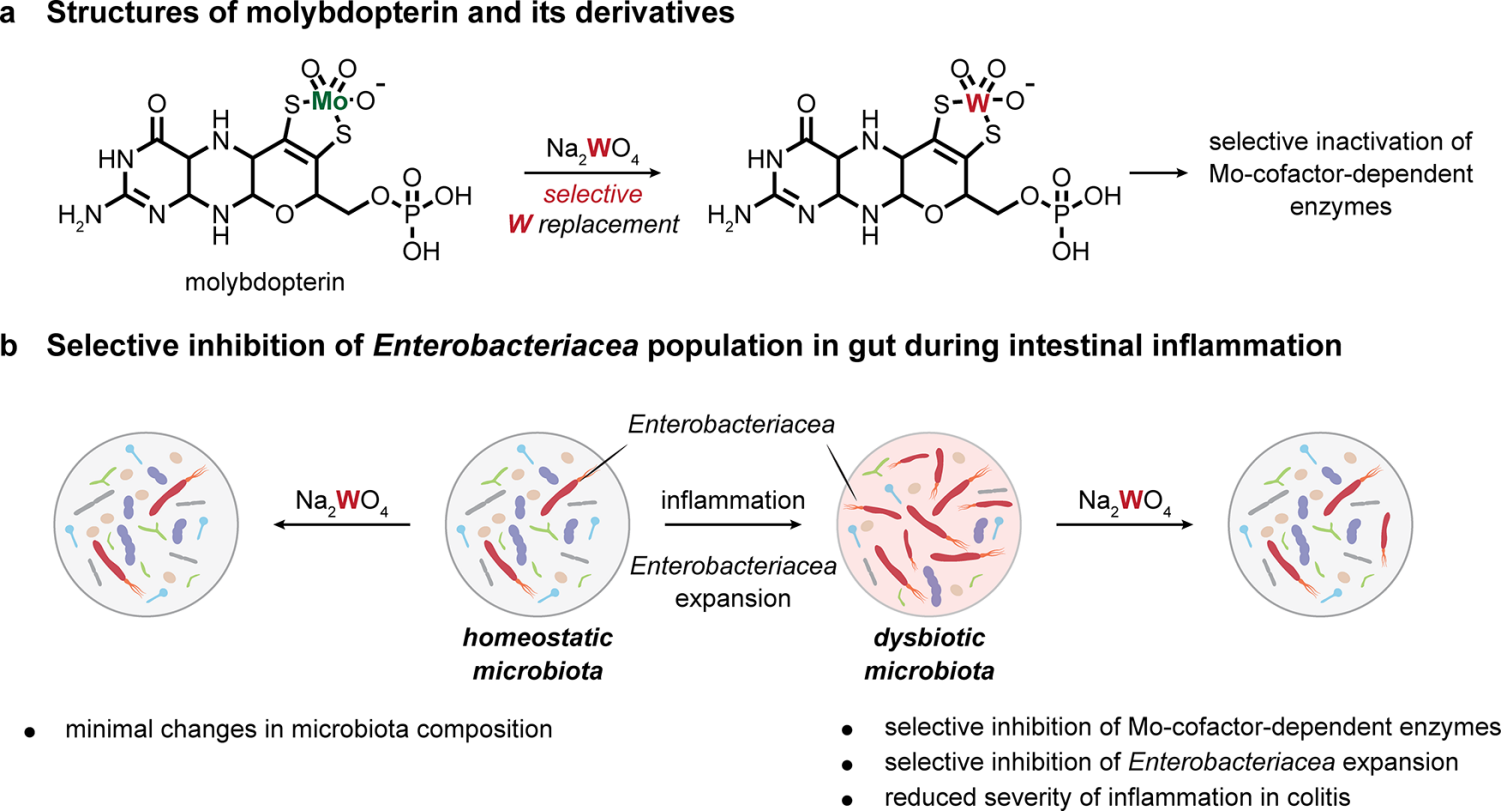
Targeting unique cofactors for organismoselectivity.^30^ (a) Molybdenum guanine dinucleotide (molybdopterin) can be effectively transmetalated by tungsten. (b) *Enterobacteriaceae* expand during gut inflammation, and this can be selectively inhibited by tungsten transmetalation without effecting the homeostatic microbiota population.

An expansion of facultative anaerobic bacteria of the *Enterobacteriaceae* family is usually observed in the inflammatory disease of the gastrointestinal tract and presents another example where the living gut is effectively used as a reaction vessel. A molybdenum-cofactor-dependent microbial respiratory pathway in *Enterobacteriaceae* is a signature of inflammation-associated dysbiosis. Remarkably, the molybdenum guanine dinucleotide form of the molybdopterin cofactor used by molybdoenzymes [Figure 2]^25^ can be effectively transmetalated by tungsten and so lead to inhibition^26−29^ (due to the lower reduction potential of W(VI)/W(V) and W(V)/W(IV) couples). Treatment of a dextran sulfate-induced colitis model with tungsten in mice can selectively inhibit the expansion of *Enterobacteriaceae* population while leaving other taxonomic families only marginally affected.^30^ Moreover, efficacy was also seen in humanized, germ-free mice using gut microbiota from patients with inflammatory bowel disease, showing that the effect of tungsten was not limited to mouse microbiota. Treatment was only effective on *Enterobacteriaceae* populations in an inflammatory state rather than homeostatic, consistent with the proposed mode of action. Impressively, therefore, although exploiting near-trivial chemical control via selective coordinate W–S (versus Mo–S) bond formation, the resulting exquisite organism-selectivity of this strategy was illustratively effective in a disease state subpopulation of a microbial community in relevant mouse models.^30^

## 3 Tissuoselectivity

The ability to selectively carry out chemistry in specific tissue in a living organism opens up further possibilities in modulating function and disease that move beyond the use of organisms as reaction vessels. A key driver in generating selectivity for a given tissue may often be its different physiological state that can then be exploited in the fine-tuning of the pharmacodynamics (PD) and pharmacokinetics (PK) of an exogenous substance. As such, when focusing on mammalian (and in particular human) tissues, consideration of tissues under a number of groupings^31−33^ based on their likely exposure to an administered compound (as well as route of administration) is of obvious relevance in achieving selectivity. One well-studied challenge (“dividing line”) is presented by the so-called blood–brain barrier (BBB) that delineates “within” from “without” the central nervous system (CNS); this can be considered a two-pool system for many compounds (or reagents).^34^ Further subdivision may then be invoked, such as blood as a circulatory tissue and associated processing tissues (*e.g.*, liver and kidney) that will extract and sequester. The limited availability of clinical data for distribution across key “barriers”, such as peritoneal–plasma,^35^ highlights the broader challenge and yet opportunities for innovative chemistries. Other tissues are more often characterized by their individual identities and physiological function.

As such, one can consider that, following intravenous administration, those tissues with the highest exposure to the circulatory system (including the heart) will have the highest *dynamic* exposure to a given compound. Those that sequester (*e.g.*, liver) and so generate a resting “sink” are likely to be less challenging to target. Moreover, an aberrant tissue/tumor may display in some cases unusual circulation networks long proposed to lead to enhanced permeability, albeit controversially^36^ (and indeed may display aberrant cellular hallmarks, such as cell-surface glycosylation that will allow combined selectivities, see section 5.1). Some will display inherent incompatibilities with certain functional groups and, therefore, associated chemistries for LSF. We aim to survey these aspects in passing.

Finally, aspects of tissue selectivity may be further enhanced by exploiting tissue-associated biomarkers or pathways. Some may be associated with the dysfunction or pathology of that tissue. For instance, the use of a tissue-surface biomarker can allow an attachment site (to localize reagents), a tissue-specific active transport path can allow tissue specific uptake (to localize, internalize, and concentrate reagents), and a tissue-specific genetic, biosynthetic, and/or metabolic pathway would allow exploitation to incorporate unnatural residues into substrates (to change chemoselective addressability). While aspects of these considerations sit on a spectrum (tissue-specific metabolic generation of a substrate, *cf*., tissue-specific hijacking of metabolism to incorporate a substrate) separate sections below explore tissuoselective aspects of so-called pretargeting^37,38^ and metabolic labeling.^39^

### 3.1 Serum, Plasma, and Blood

Serum or plasma protein binding assays are used widely in potentially^40^ delineating the pharmacological efficacy of small molecule therapeutics. Therefore, it is apparent that essentially identical considerations apply to reagents that might be used for LSF in serum or that, following *iv* administration, will be transported via blood. This will be exacerbated by their potential reactivity.

It is therefore relevant that apparent serum reactivity of cyclooctynes (Western blot analysis that suggests covalent linkage)^41^ is likely linked to the confirmed *in vitro* reactivity of Cys residues within serum albumin with alkynes.^42^ This has been suggested to account for differing *in vivo* selectivities. Independent, careful evaluation of such early generation strained alkyne derivatives, which are shown to modify proteins *in vitro*, have similarly revealed serum interactions that led to sequestering in pretargeting (see section 3.6) and so limit their use *in vivo*.^43^

### 3.2 Liver

**Figure 3:**
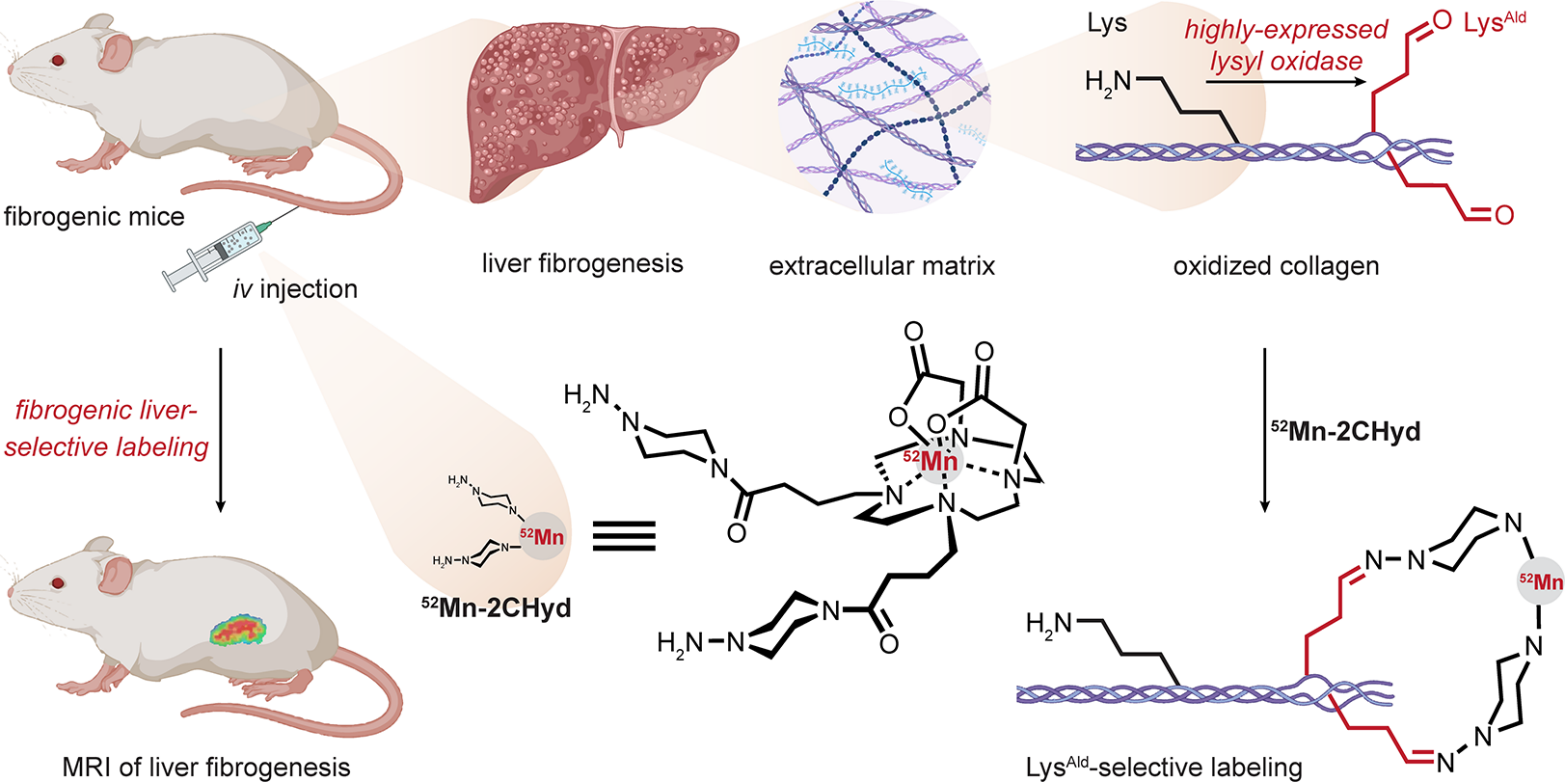
Allysine creates tissuoselectivity in the liver. Lysyl oxidase is overexpressed in liver fibrogenesis, resulting in the accumulation of allysine modification. Liver fibrogenesis could be selectively detected using MRI with a hydrazine-labeled manganese complex as a contrast agent.^44,45^

Liver fibrogenesis is accompanied by the upregulation of the lysyl oxidase (LOX) enzyme. This enzyme, normally localized to smooth muscle and cardiomyocytes at lower levels, catalyzes the oxidation of the terminal amino group on lysine residues to form aldehydes in so-called allysine (Lys^Ald^); this typically leads to diverse cross-linking pathways, often in the extracellular matrix of tissues. Its upregulation therefore generates potential tissuoselective reactivity. By simply targeting aldehyde with ^52^Mn(II) complexes bearing hydrazides, liver tissue in mouse models of fibrogenesis was selectively labeled (effectively both disease and tissue selectivity) [Figure 3].^44,45^ This study usefully exemplifies that combined modes of selectivity may drive desired modifications. Here that includes residue selectivity (as one mode of chemoselectivity) for Lys residues that are oxidized with additional chemoselectivity via the resultant aldehydes that are of very low natural abundance. Moreover, tissue selectivity arises from the overexpression of an enzyme specific to the disease.

### 3.3 Muscle

**Figure 4:**
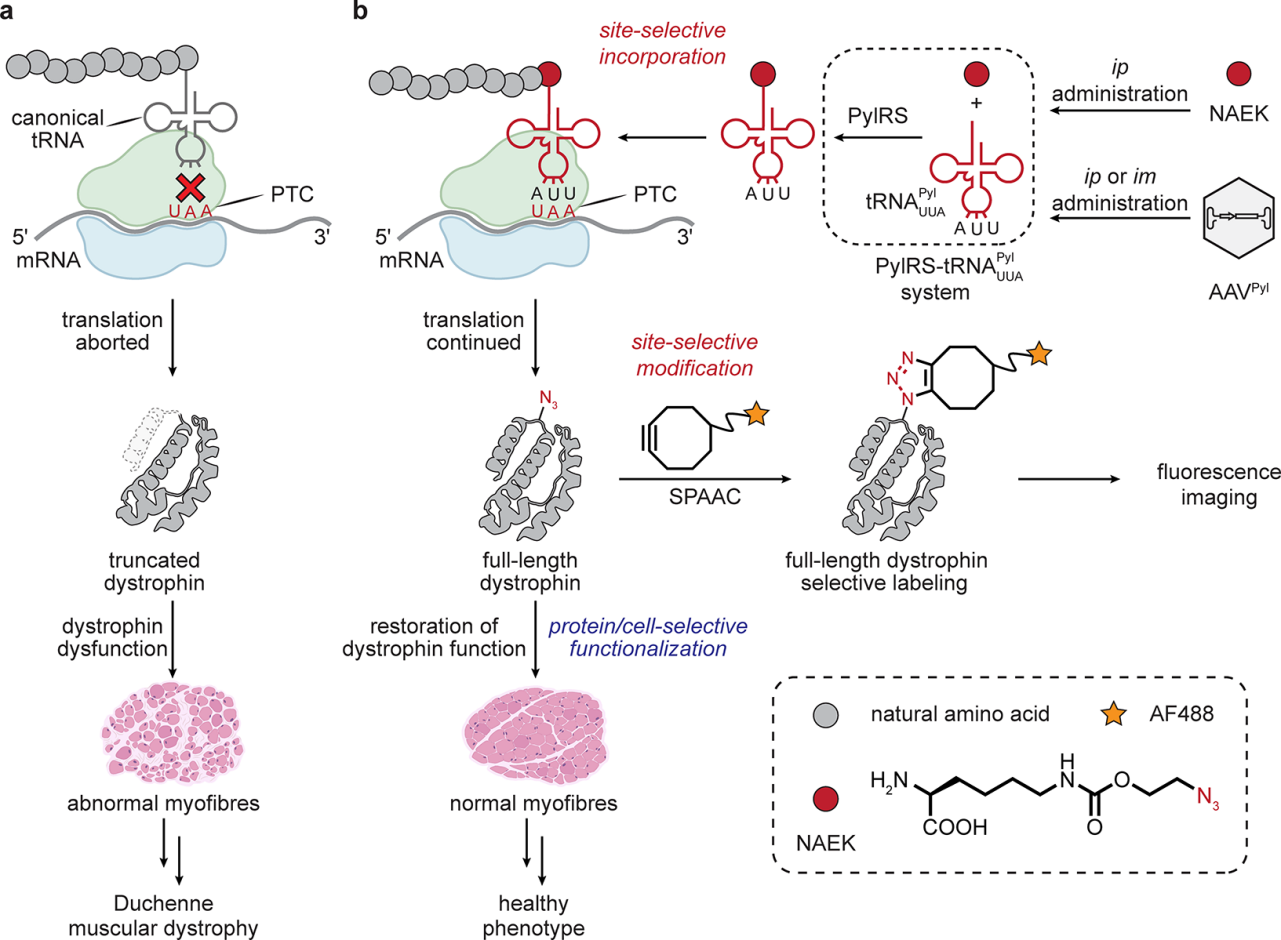
Tissue-associated pathology can create a target for tissuoselectivity.^49^ Protein function restoration by suppression with uAA in muscle. i.m. administration = intramuscular administration.

Tissue-associated pathology can provide additional windows into selectivity. Duchenne muscular dystrophy (DMD) is caused by a nonsense mutation in the *DMD* gene encoding dystrophin, leading to progressive muscle weakness. It is an example of a monogenic disease arising from unwanted termination at a UAA stop-codon site (a so-called premature termination codon (PTC)) that leads to truncated gene products. Suppression of termination at stop (often at amber UAG) sites is a typical aspect of genetic code expansion strategies.^46^ Adoption of one of the primary tool sets of stop codon suppression in one provocative study (a *Methanosarcina mazei* PylRS-tRNA^Pyl^ pair along with coadministered unnatural amino acid (uAA) *N*^ε^-2-azidoethyloxycarbonyl-l-lysine (NAEK)) allowed suppression and thus expression of the full-length protein, leading to a corrected state with only minor loss of function in differentiated myoblasts derived from mice and patients bearing the nonsense mutation. Restoration of the function of dystrophin *in vivo* was shown by administering the genes of the PylRS-tRNA pair via an adeno-associated virus AAV2/9 system as well as intraperitoneal (*ip*) injection of the uAA. No effects caused by background suppression of “correct” termination were observed, despite the potential for disruption; this may be a consequence of the very different context of these premature stop sites as compared to normal.^47,48^ Although in this example there is no inherent selectivity of the AAV2/9 system for muscle, the predominant presence of the *DMD* transcript in muscle highlights a tissue-specific trait. While LSF was only employed in this study to fluorescently label *ex vivo* muscle tissue sections (via SPAAC) [Figure 4], it raises the possibility of using a similar approach to install amino acids that might subsequently generate novel or improved function.^49^

### 3.4 Cancerous Tissue: Tissuoselectivity via a Generally Altered Biochemical State

While generalizations in a disease category that is associated with many different cell and tissue types should be treated with obvious caution, efficacy has been demonstrated by suggested exploitation of tissuoselective biochemical alterations in cancer.

**Figure 5:**
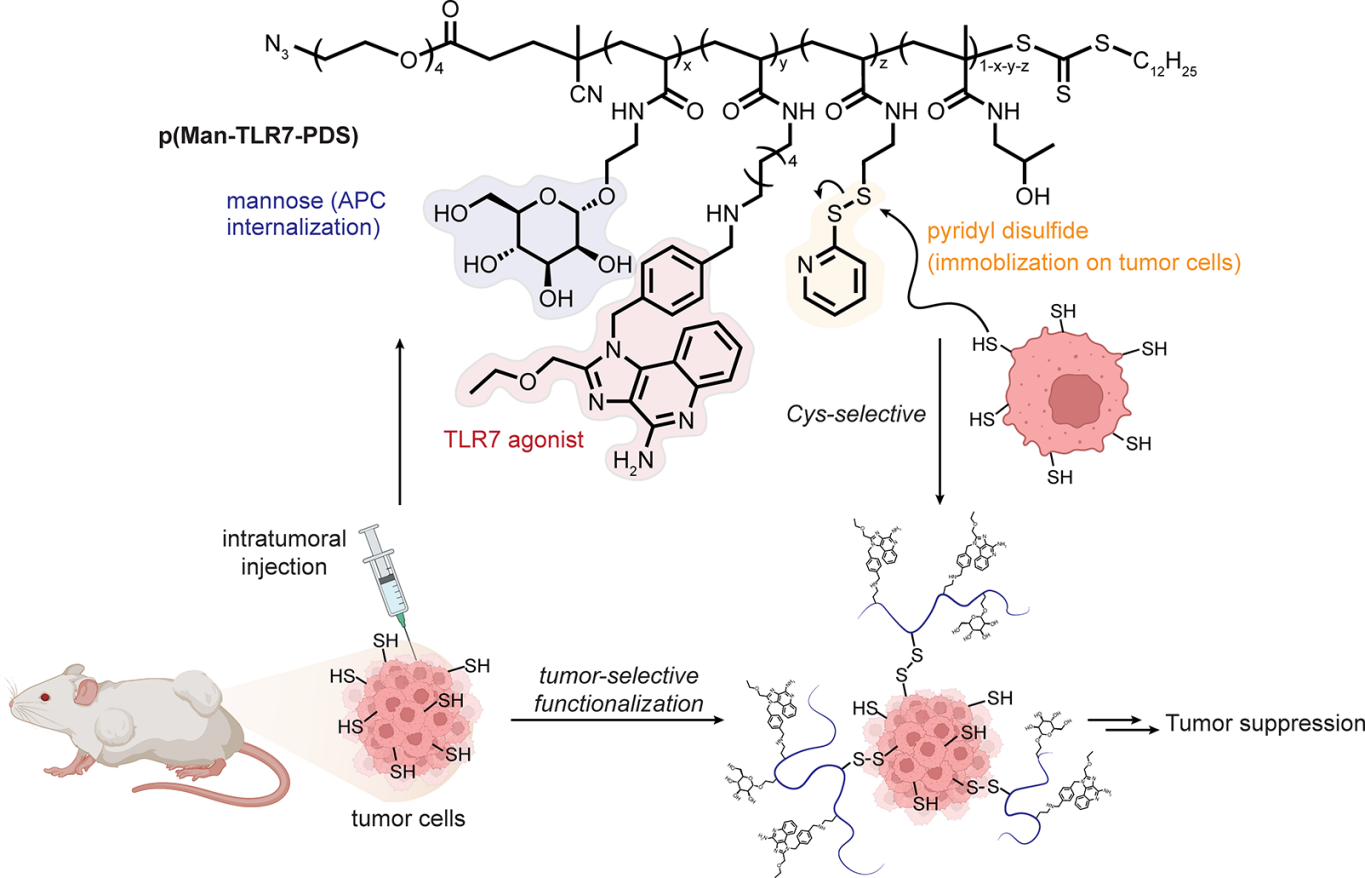
An altered biochemical state can create a target for tissuoselectivity. The reductive tumor extracellular environment was selectively targeted by use of the classical pyridyldisulfide motif to install polymers able to recruit immune effector cells through TLR7 agonism.^53^ Abbreviations are as follows: Man = mannoside, PDS = pyridyldisulfide, and APC = antigen presenting cell.

The extracellular environment of cancer cells is considered to be reductive^50,51^ and so potentially more likely to bear free thiols, suggesting exploitation of tissuoselective reactivity even with thiols (perhaps surprising as one of the most generic functional groups in nature). In a bold example, polymers containing the long-known thiol-reactive^52^ pyridyldisulfide moiety, additionally bearing Toll-like receptor 7 (TLR7) agonists and d-mannosides, have been proposed as recruiters of immune effector cells via the formation of asymmetric disulfides with free thiols in cancerous tissue. Mice with MC38 colon carcinoma or B16F10 melanoma showed significant slowing of tumor growth compared to those treated with a polymer control that did not possess the pyridyldisulfide moiety [Figure 5].^53^

**Figure 6:**
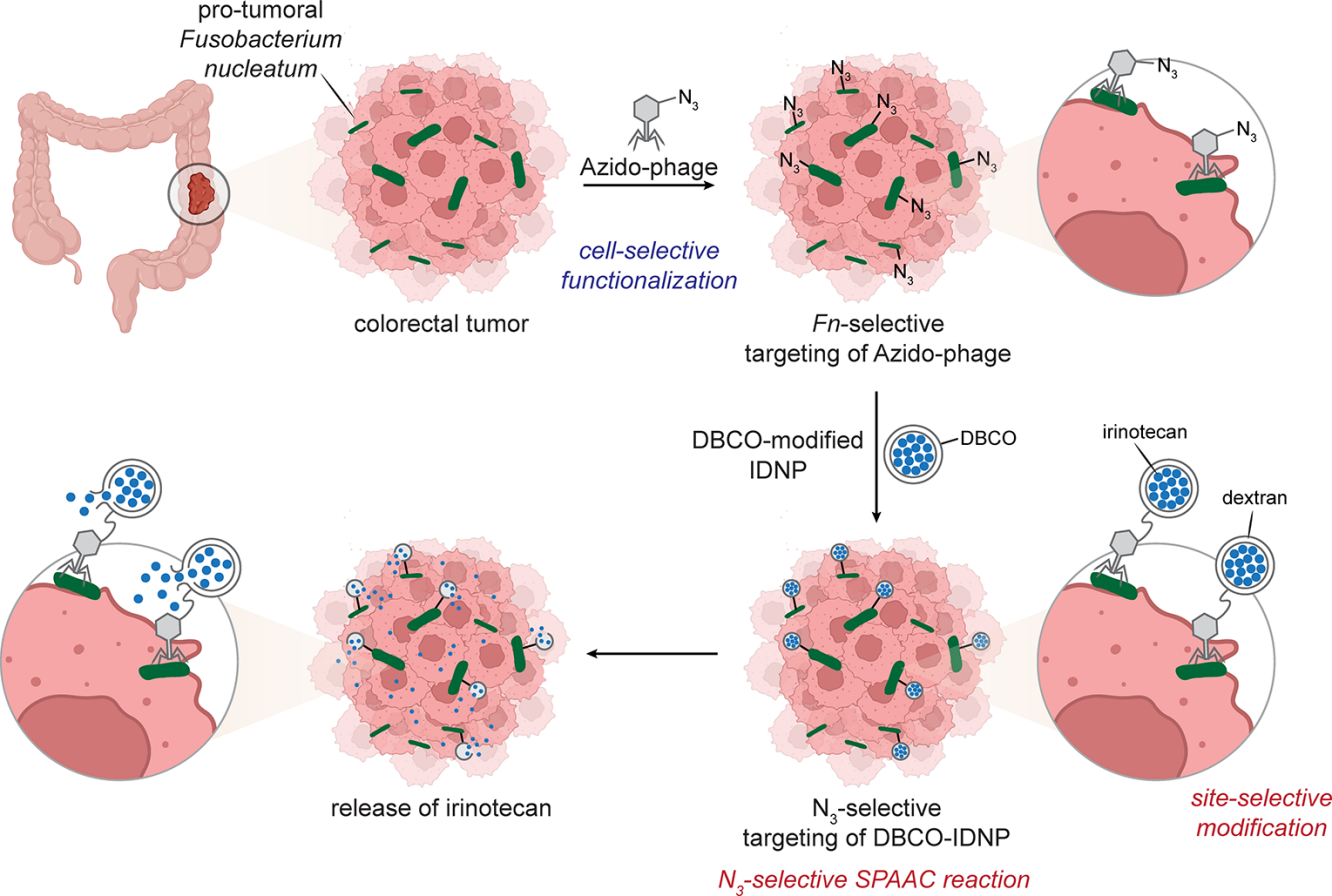
Tissuoselectivity gained by “piggy-backing” microbes. The pro-tumoral bacteria *F. nucleatum* was targeted by a selective azido-phage. Tumours thus displaying azides could be targeted with DBCO-labeled reagents (*e.g.*, nanoparticles that release cytotoxic agent irinotecan).^54^ Abbreviations are as follows: IDNP = irinotecan-loaded dextran nanoparticle.

In colorectal cancer, via mechanisms that are yet to be fully elucidated, the bacterium *Fusobacterium nucleatum* (*Fn*) is known to be both pro-tumoral and exist at higher concentrations in associated cancerous tissue. An intriguing approach^54^ has explored exploitation via LSF in an *in vivo* mouse model of colorectal cancer that has been precolonized with *Fn*. Azide-displaying *Fn*-selective phages were generated by “culturing” the phage inside a *F. nucleatum* bacteria in which azidohomoalanine (Aha) was added to the culture media, resulting in incorporation of azides into the phage. When mice were treated with the *Fn*-selective “azido-phage”, selective accumulation resulted. The use of carboxymethyl dextran-derived DBCO-functionalized nanoparticles encapsulating irinotecan as a cytotoxic agent allowed an apparently selective conjugation reaction at *Fn* bacteria localized in tumor tissue. Mice treated with both phage and nanoparticle displayed the smallest tumor volume suggesting efficacy in a chemically augmented approach to antitumor therapy. This lodging of bacterial species as a tissue-specific pseudobiomarker suggests a tissuoselective LSF approach that is somewhat reminiscent of molecular pretargeting approaches [Figure 6].

### 3.5 Metabolic Incorporation into Tissues: Tissuoselectivity via Metabolism

When endogenous tissue-selective traits for the chemistry of choice prove insufficient, it may be possible to locally endow traits or exploit precursor-associated traits of a tissue to change its role as a chemical substrate. One potential example of this is through the incorporation of altered precursors that are tolerated by metabolism. Different tissue-dependent tolerance then, in principle, could give rise to a certain tissue displaying a different chemistry based on differential tolerance.

The incorporation of modified metabolic precursors exploits the auxotrophic activity of living systems and so, in the context of the selectivities that we discuss here, is primarily relevant at the levels of celluloselectivity and above. Indeed, despite long-standing origins in organismal systems, most current examples of metabolic labeling only exploit cells; put crudely, if bathed in a precursor via culture medium, then cells may take stuff up.

The smuggling of unnatural (whether in functional group type or location) moieties into an organism is an obviously useful method for installing a precursor that can be further elaborated through (largely chemo-) selective chemistry, thereby creating opportunities for LSF. The method is long-established^55−58^ and at one level reflects the distribution of solute carriers found in tissue and cells,^59^ leading to a focus on unnatural amino acid (uAA) and unnatural carbohydrate/glycan (uG) residues. This can be enhanced by the use of obligate auxotrophs^60,61^ to drive/ensure higher incorporation but has found wide use simply through feeding to organisms, tissues, or cells. We explore this here as a general category of the tissuoselectivity level, as this is where broadly relevant opportunities in uptake variation might in principle be most exploited (one tissue over another), although many aspects have typically focused on cell culture (one cell over another). Some species-level selectivities noted above also are at one level, exploiting such differences.

**Figure 7:**
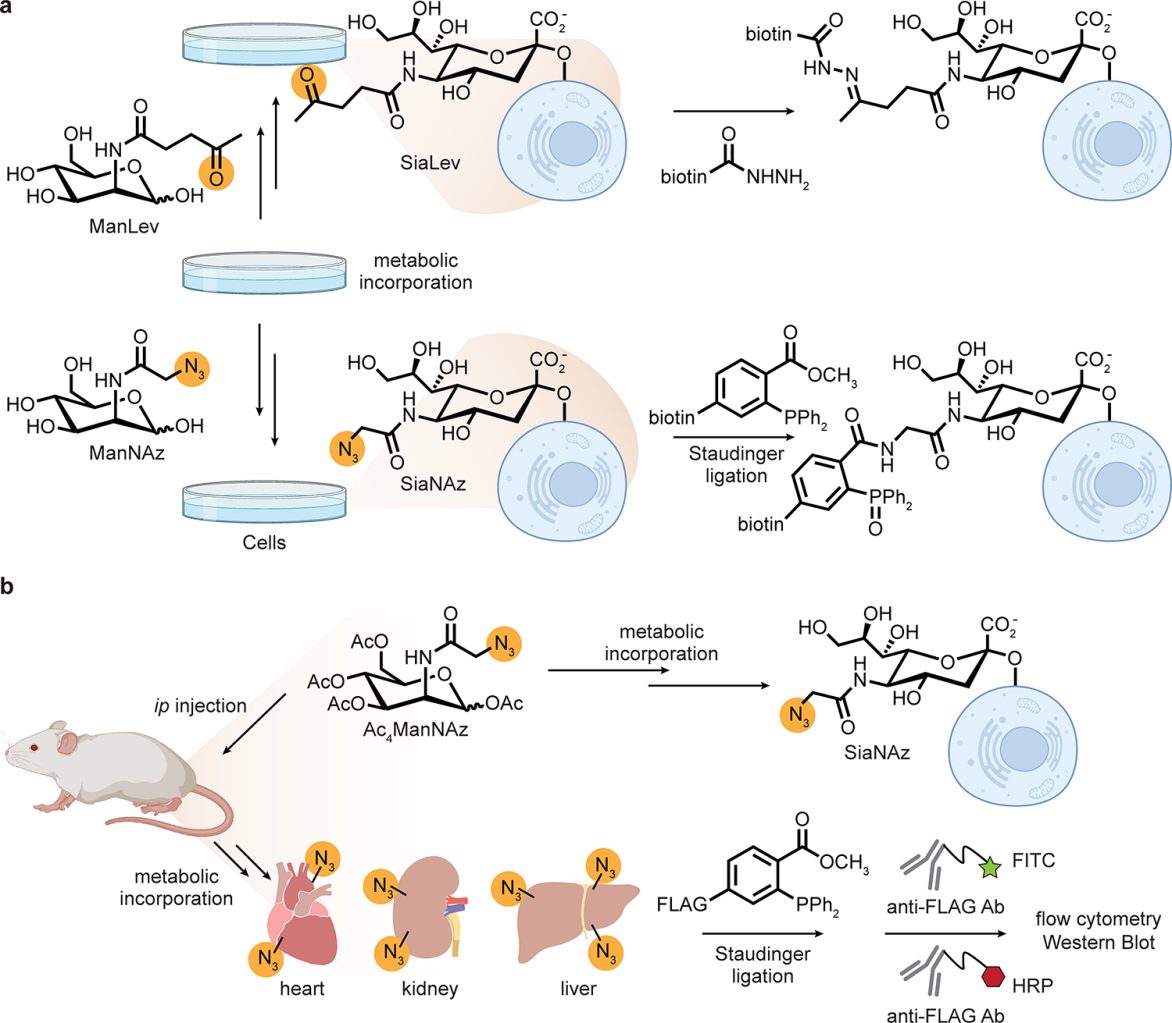
Metabolic incorporation of modified sialic acids through the glycan salvage pathway. (a) Incorporation of ketones or azides into cell-surface sialic acids via *N*-acyl-altered d-mannose derivatives followed by chemoselective reaction with acylhydrazides or through the Staudinger reaction, respectively. (b) Incorporation of azido sialic acid into a living organism and detection through Staudinger ligation via the FLAG epitope tag. HRP = horseradish peroxidase, FITC = fluorescein isothiocyanate. This strategy revealed tissue selectivity for the heart, kidney, and liver.^39,62−65^

Salvage pathways for (re)uptake of metabolically expensive precursors provide a key opportunity. For carbohydrates, glycan salvage pathways allow use of modified glycans in biosynthesis [Figure 7]. Indeed this was elegantly demonstrated by Reutter^39,62^ in the early 1990s: *N*-acyl-altered variants of the sugars ManNAc and GlcNAc were taken up and then tolerated during biosynthetic elaboration to higher carbon sugar sialic acids. These, in turn, were incorporated into complex bioconjugates, thereby displaying altered chemical moieties in cell-surface glycans in tissues in a living mouse. This systemic incorporation of modified glycans showed apparent tissue-based selectivity dependent on the size of the *N*-acyl modification and the nature of the donor sugar.^39^*N*-Propanoyl-d-mannosamine gave the most efficient wide-scale incorporation into cellular glycoproteins.

This observation was ingeniously used by Bertozzi for cell-surface chemistry through the additional display of a ketone handle/tag (via appropriate *N*-acyl-ManNAc); efficient display on cell-surface glycoproteins allowed subsequent modification with acyl hydrazides to form stable hydrazone adducts.^63^ This work was further extended to the azido-acyl variant of ManNAc, leading to the large body of work and widespread use of this method as a source of the azide moiety in living systems—this is now a frequently applied motif of chemical biology. The first reactions were performed via a modified Staudinger reaction on cells (a Staudinger ligation (SL)).^64^ Ligation with a “FLAG”-peptide bearing phosphine *in vivo* followed by analysis of splenocytes by flow cytometry against the antibody (Ab)-detectable epitope revealed qualitatively successful *in vivo* Staudinger reaction.^65^ Notably, splenocytes express high levels of sialosides; therefore, selectivity was likely already imparted by the spleen and by these cells. *Ex vivo* late-stage functionalization of harvested organs revealed further tissue-level selectivity in the incorporation of the azide tag: only the heart, kidney, and liver revealed the presence of azide (no reactivity was observed in the brain or thymus). The liver is responsible for first-pass metabolism and also expresses a high level of cell-surface sialosides. The kidney and heart express lower levels of UDP-GlcNAc 2-epimerase (which produces ManNAc), suggesting a greater “auxotrophic need”. As such, this highlights a nice example of exploiting differential enzyme expression to impart tissue selectivity.^65^ Notably, the phosphine-FLAG reagent fairs better in terms of splenocyte modification efficiency than a corresponding difluorocyclooctyne despite slower *in vitro* kinetics in small-molecule systems.^41^ This may be attributable to apparent serum reactivity of cyclooctynes,^41^ which is potentially linked to their known *in vitro* reactivity with serum albumin (see section 3.1).^42^

A complementary strategy using the azido variant of GalNAc (GalNAz, to label *O*-linked mucin type cell surface glycans) in live zebrafish allowed for tissue-targeted labeling of azide-modified cell surface glycans using a temporally selective approach. Specifically, by adding the metabolic precursor and labeling, followed by further addition and labeling with a different colored fluorophore (pulsing and chasing), time and consequential tissue selective modification revealed different spatiotemporal mucin production. As such, here the tissuoselectivity relies on the altering “tissues” of a zebrafish embryo in the early stages of its development. Therefore, while different tissues are beautifully labeled, given the changing and dynamic nature of embryo physiology, the driving selectivity here is likely for new proteins being expressed rather than intrinsic selectivity for a given tissue *per se*.^66^

**Figure 8:**
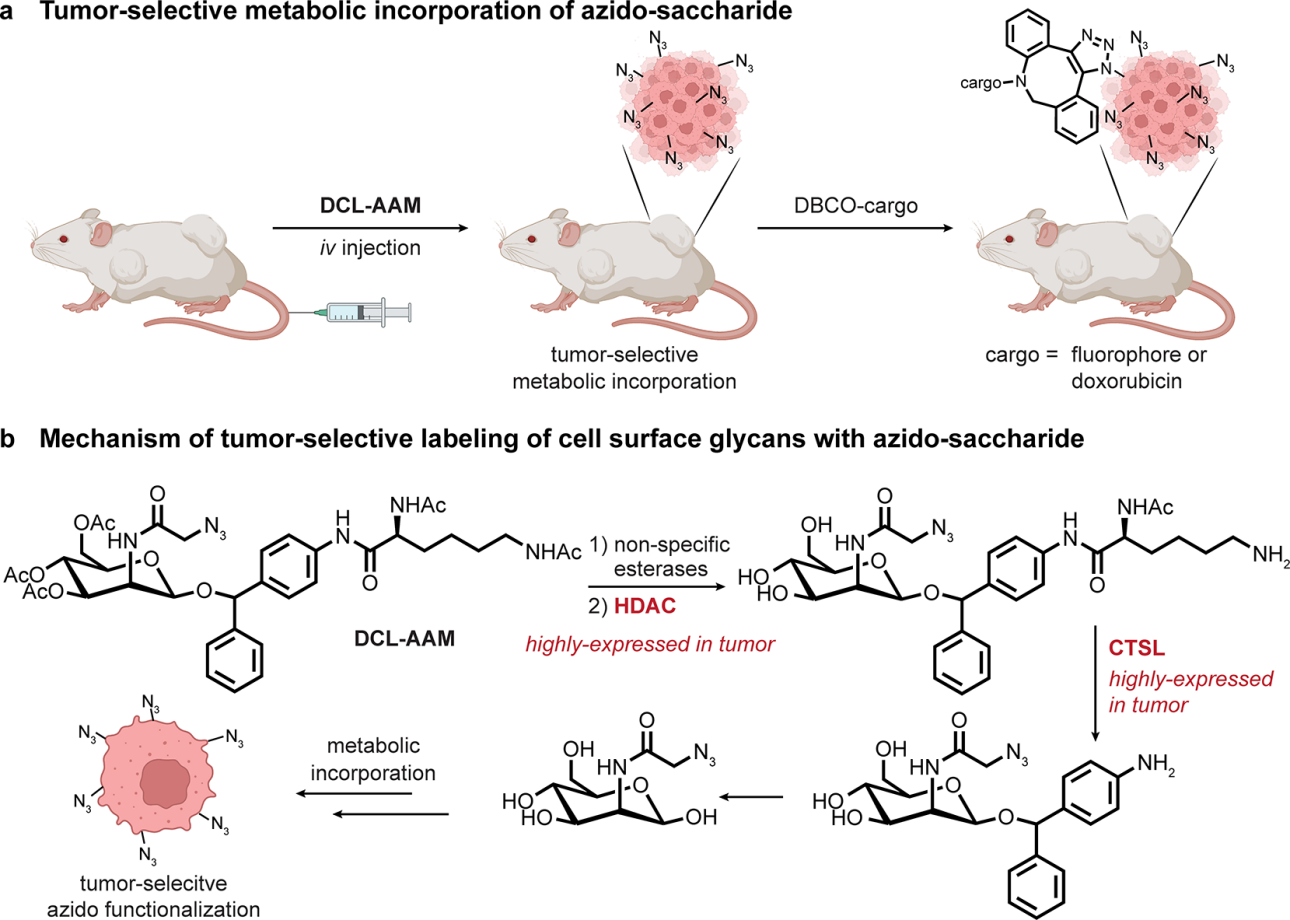
Tissuoselectivity via a “pro-metabolite” for metabolic labeling. (a) Incorporation of azides into tumor tissue as a strategy for selective tumor tissue targeting. (b) An *O*-1-protected ManAz “pro-metabolite”—named DCL-AAM—is suggested as being selectively deprotected through the sequential action of two enzymes that are overexpressed in tumors, allowing for selective incorporation into cell-surface glycans in that tissue.^68^ Abbreviations are as follows: CTSL = cathepsin L.

The potential role of tissue-specific metabolism in the context of metabolic labeling and tissuoselectivity is illustrated by a proof-of-principle study [Figure 8], which suggested the efficacy of tissue-specific enzyme-mediated release of certain sugar precursors that are used to incorporate tags. For sugar-mediated metabolic labeling, peracetylated sugars are typically used as precursors.^67^ By considering cancerous tissue as a class of pseudo-distinctive tissue, it has been suggested that overexpression of histone deacetylase (HDAC) enzymes and cathepsins found in some cancerous tissues might together engender selectivity in the uptake and/or use of an acetylated azido-sugar that instead bears an anomeric substituent (specifically, an ether linked diphenylamino moiety with a pendant diacetyl-lysine [Figure 8b]) requiring sequential HDAC (for the ε-NH_2_)-then-cathepsin (for the α-NH_2_)-mediated release of this anomeric group. The seemingly enhanced presentation of azido-sialic acid on cell surfaces of cancers in living mice was imaged with DBCO-Cy5 dye and targeted with DBCO-conjugated-doxorubicin, thereby increasing effective killing over doxorubicin alone.^68^

It should be noted that peracetylated sugars can act as precursors to α,β-unsaturated open-chain aldehydes^69^ that may drive nonspecific-Cys modification.^70^ Therefore, given that few *in vivo* studies have yet fully characterized the nature of observed conjugates (*i.e.*, the linkages formed) and verification is typically performed by imaging and/or epitope detection, the involvement of additional chemistries or introduction of other structures cannot perhaps be discounted in all cases. These “artificial” *S*-glycosylations^70^ highlight that some displayed azides might instead be a result of Cys-modification rather than metabolic incorporation into biosynthetic pathways. 1,3-di-*O*-propionyl-variants have since been proposed as improved precursors.^69^

**Figure 9:**
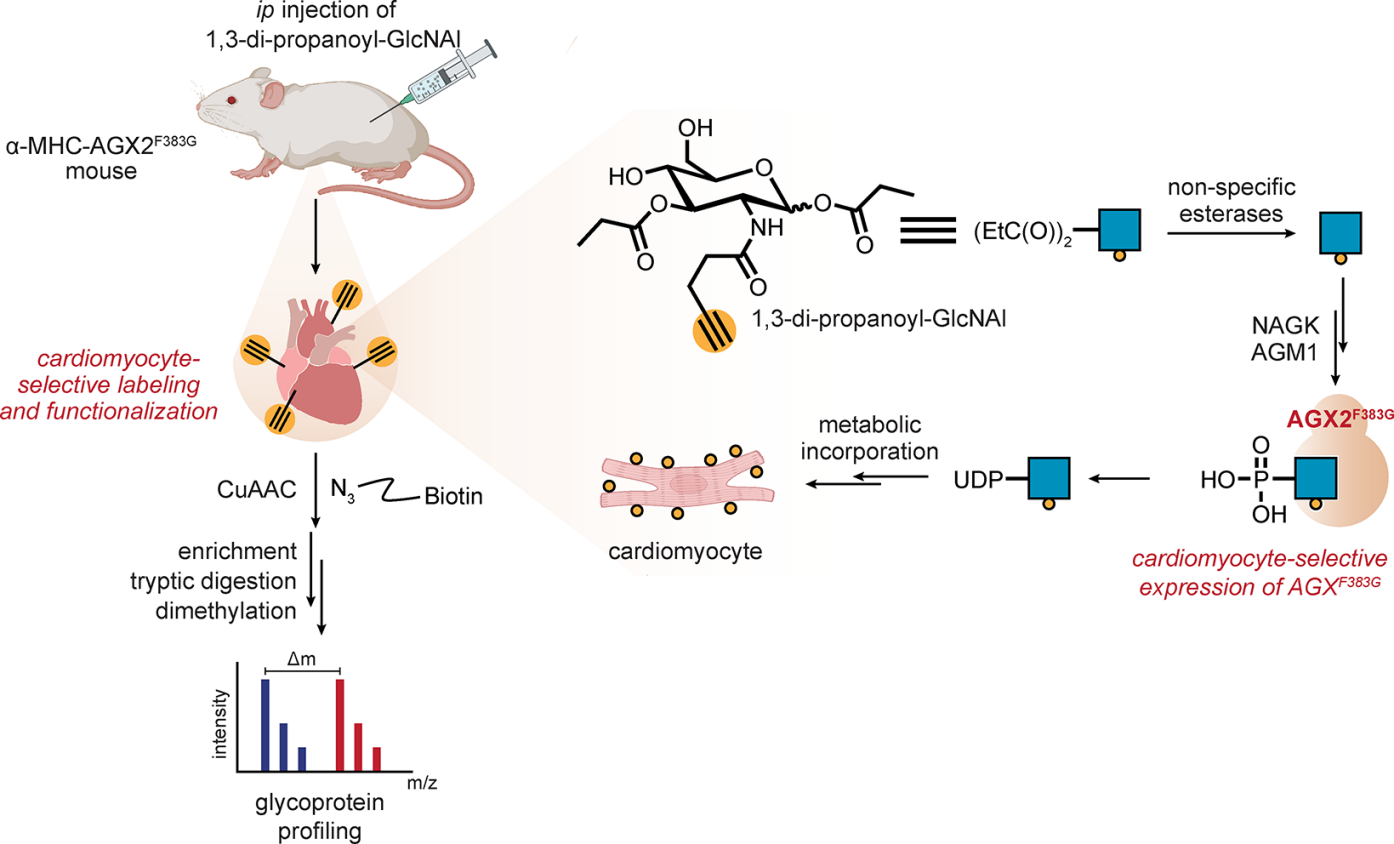
Tissuoselectivity via tissue-specific alteration of metabolic enzymes. Cardiomyocytes were genetically engineered to express a mutant pyrophosphorylase, resulting in tissue-selective incorporation of an alkynyl-GlcNAc variant into mouse hearts after systemic dosing of a metabolic precursor that could be processed by that enzyme. Abbreviations are as follows: NAGK = *N*-acetyl-d-glucosamine kinase, and AGM1 = *N*-acetylglucosamine-phosphate mutase 1.^71^

Such ideas suggest that manipulation of endogenous enzymes may prove a powerful driver of tissue-selective incorporation and, hence, LSF. One example shows that this can be achieved by the manipulation of host biosynthetic machinery. The creation of a “holed” UDP-GlcNAc pyrophosphorylase mutant (AGX2^F383G^) in combination with “bumped” 1,3-di-*O*-propionyl-*N*-pentynylacetyl-d-glucosamine (1,3-EtC(O)_2_GlcNAl) treatment allows eventual conversion to UDP-GlcNAl and incorporation into cell-membrane glycans [Figure 9]. Expression of AGX2^F383G^ within the cardiomyocytes of a transgenic mouse line allowed (following several intraperitoneal doses) *ex vivo* labeling of glycans on cardiomyocyte (but interestingly not fibroblast) surfaces (with azido-TAMRA) with strong selectivity. It also proved initially useful in chemoproteomic analyses of associated glycoproteins. Here, while tissue selectivity is imparted through artificially induced genetic means (in which cardiomyocytes expressed mutant enzyme and thus were the only cell type able to utilize this metabolic precursor), this study nonetheless elegantly highlights striking potential to exploit unique tissue-associated genetic markers.^71^

Tissue-directed expression can also be powerfully exploited in the interrogation of a tissue- or organ-specific proteome using retrieval methods in chemical proteomics. Such approaches valuably complement the competitive methods (discussed below) that rely upon the plasticity of certain aminoacyl-tRNA synthetases (aaRSs). In one elegant example the use of plastic PylRS variants along with specifically anticodon-altered PyltRNA^XXX^ variants allowed expansion of this stochastic approach to six other target protein residue types with the added advantage of tissue-directed expression of the associated machinery. In this way, protein content from, for example, fly ovaries could be selectively interrogated via *ex vivo* cyclopropane-tetrazine (Tz) (IEDDA-mediated retrieval in a dissection-independent manner. This interestingly revealed hidden hallmarks of protein transport when compared with dissection-dependent classical methods.^72^

**Figure 10:**
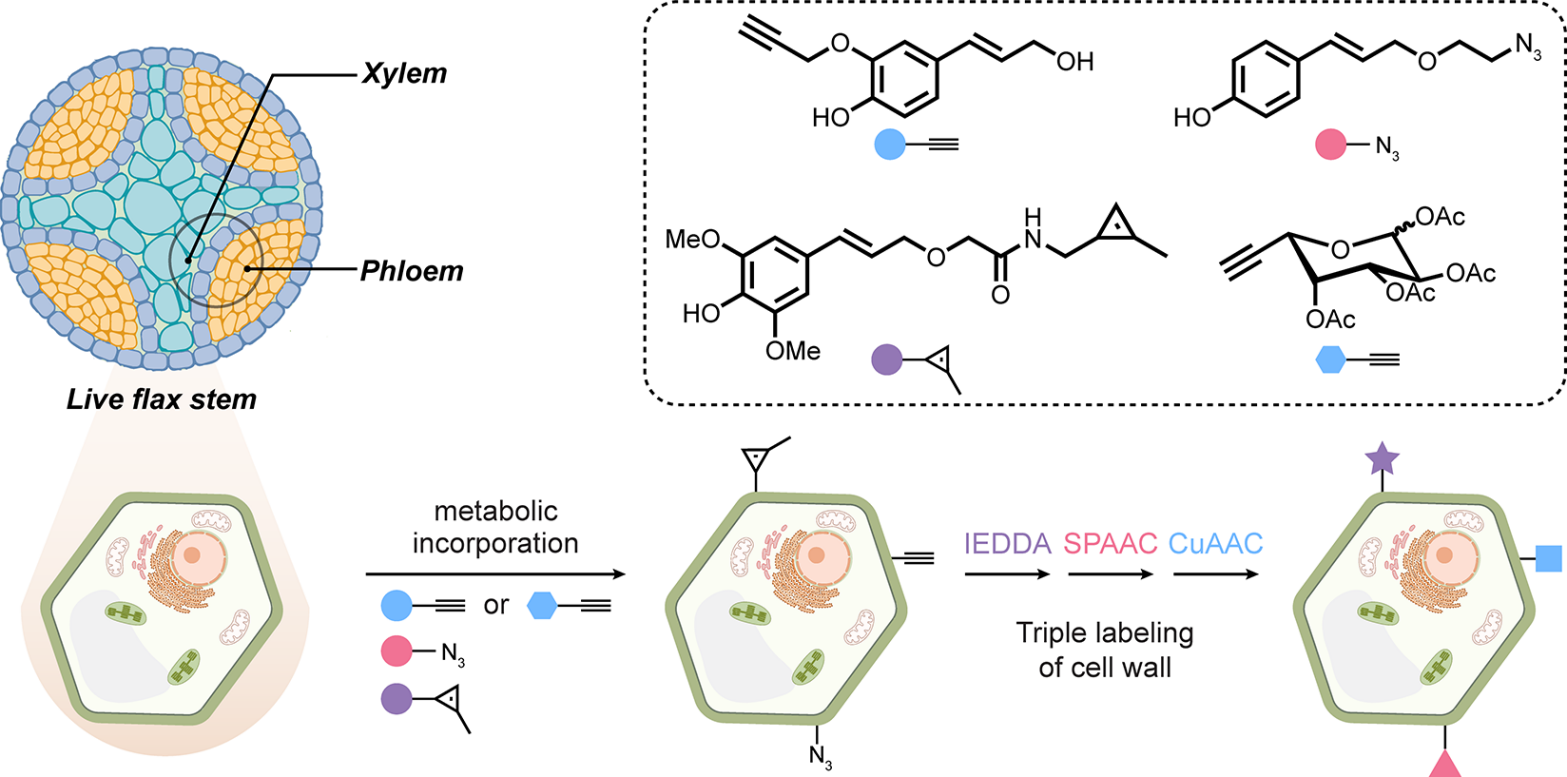
Cell wall-selective labeling of plant tissues. Incorporation of three lignin precursors bearing orthogonal chemical handles and a noncellulose precursor allows for the labeling of distinct parts of the plant cell wall tissue.^73^

It is apparent that tissue architectures are highly species-dependent. In plants, cell wall-specific incorporation of unnatural lignin monomers has extended metabolic strategies elegantly, with strongly endowed tissue selectivity for subsequent LSF. Three different lignin precursors (applied to the base of a cut stem) illustrated applicability to *ex vivo* (tissue slice) CuAAC, SPAAC, or IEDDA with different fluorophores [Figure 10]. Interestingly, these distinguished different parts of the cell-wall, suggesting that even a different “subtissue” selectivity could also be achieved based on regions of plant cell wall composed of different ratios of the three lignin precursors. When combined with a noncellulose precursor (an alkynyl-l-fucose), further layers of selective chemistries allowed further cell wall stratification.^73^

### 3.6 Tissue Pretargeting: Tissuoselectivity via Tissue-Surface Markers

The differential kinetics of circulation, clearance, or indeed chemistries (including radioactive decay) may lead to incompatible time regimes for efficacy in various applications. One elegant solution first explored using biotin–avidin noncovalent affinity methods, namely, pretargeting,^37,38^ is to target a tissue type, typically with an antibody raised against a relevant tissue-specific “biomarker”, under one time frame (*e.g.*, allowing clearance and off-target effects to pass) and to then target that localized agent via affinity methods at a tissue-specific site.

Different tissue types may be distinguished by the differential level of expression of certain extracellular components, *e.g.*, proteoglycans or receptors (as “biomarkers”). This has allowed for the targeting of specific tissue types based on these molecules and has been extensively exploited for antibody therapies, for example. This strategy has also been utilized to pretarget a tissue with a reactive group (*e.g.*, loaded onto a prelocalized antibody) to allow for subsequent late-stage functionalization of that tissue type selectively, albeit through the localization of an auxiliary.

**Figure 11:**
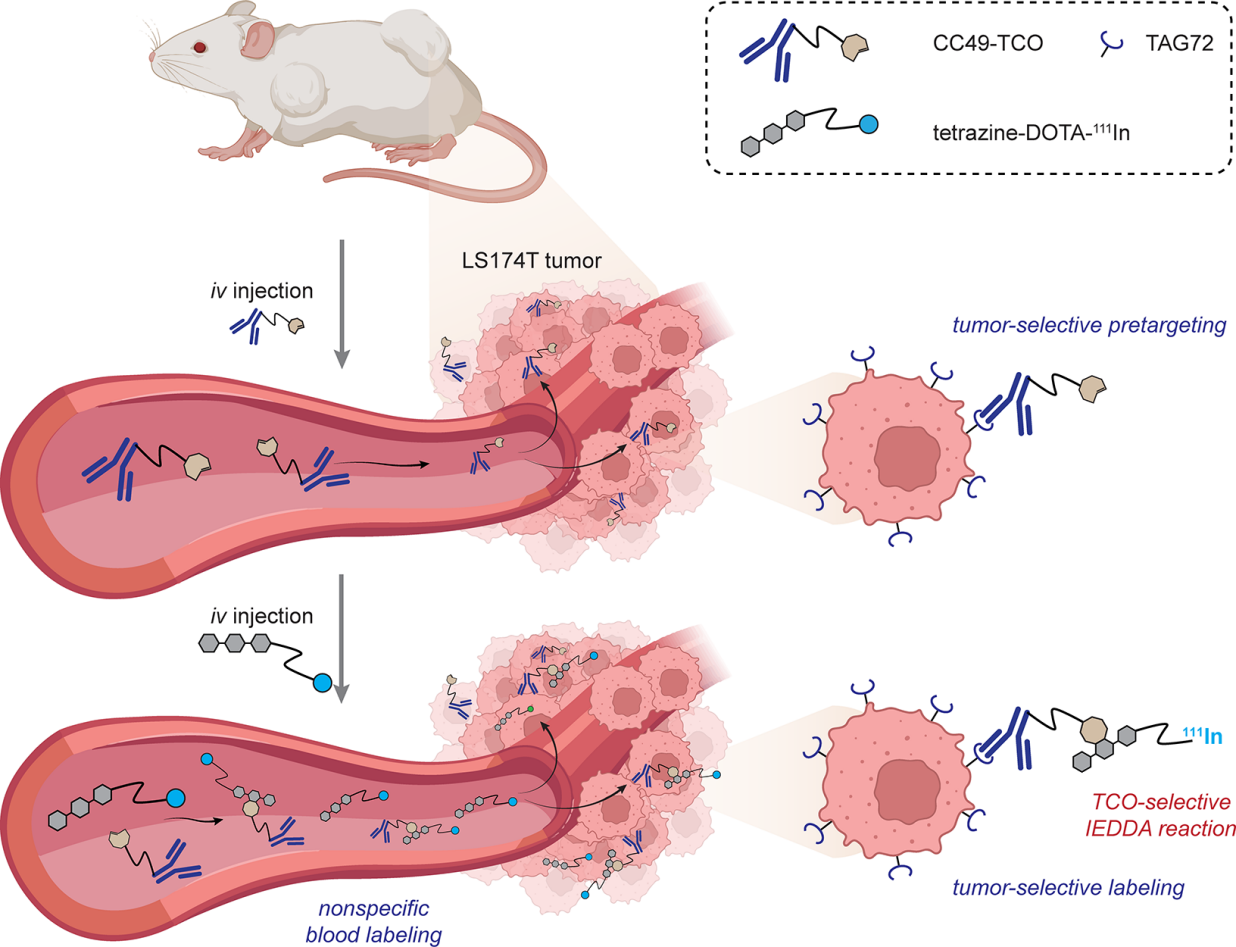
IEDDA-enabled pretargeting for tumor-specific imaging.^74^ This demonstrated the first *in vivo* use of a TCO-Tz reaction.

Robillard demonstrated early proofs-of-principle (and the first *in vivotrans*-cyclooctene (TCO)-tetrazine (Tz) reaction): a first example in mice (implanted with human colon cancer cell line LS174T) used an antitumor-associated glycoprotein (anti-TAG72)-IgG labeled statistically with TCOs via NHS chemistry followed in 24 h by ^111^In-DOTA conjugated to a tetrazine. Pronounced localization of the radionuclide at the tumor site was visualized through single-photon emission computed tomography/computed tomography (SPECT/CT) imaging with a tumor/muscle ratio of 13.1:1 [Figure 11].^74^ Notably, nonspecific blood labeling was also seen (see also section 3.1).

Use of a *trans*-cyclooctene (TCO)-bearing antihuman epidermal growth factor receptor 2 (Her2)-Ab (a modified trastuzumab) has similarly been addressed in a pretargeting variant. A tetrazine-modified albumin loaded with paclitaxel (via reaction of a corresponding *O*-succinyl-NHS ester with Lys) was used as the second “chasing” reaction. This led to apparently enhanced internalization of the albumin–drug–carrier complex into Her2(+) cells in a tumor implant in mice (inoculated with Her2(+) BT-474 human breast cancer cells).^75^

**Figure 12:**
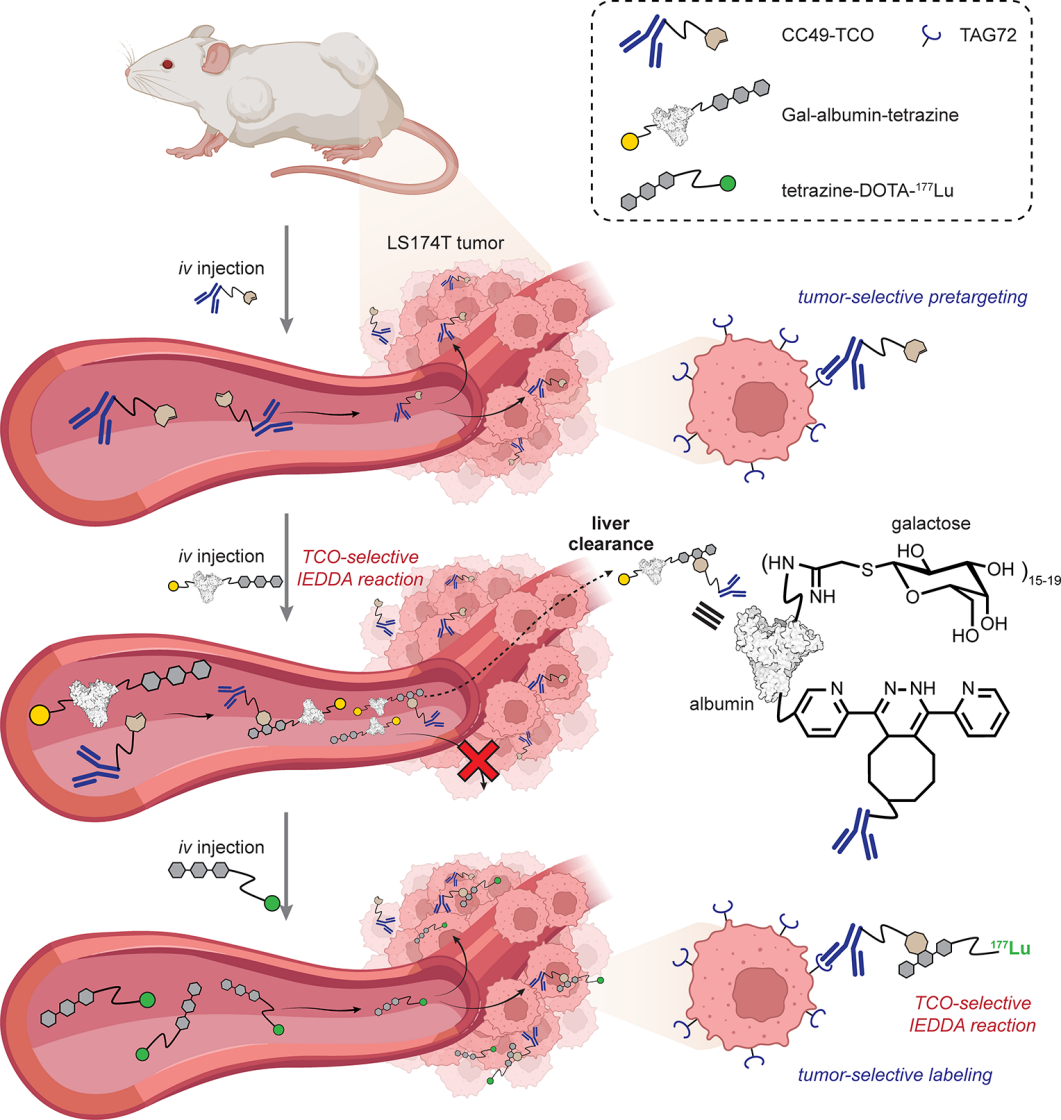
Control of off-target blood labeling in pretargeting approaches using an intervening liver-targeted synthetic glycoprotein for clearance.^76^ TAG72 = tumor-associated glycoprotein 72.

Off-target blood labeling or reactivity has been seen in several systems exploring pretargeting with TCOs^74^ or with strained alkynes via pretargeting^43^ or direct^41^ targeting (see section 3.1). Careful dual isotope (^177^Lu/^125^I) evaluation of some early generation strained alkyne derivatives revealed corresponding reactivities or efficacies too low for pretargeting to azido-Abs (rituximab with statistical azide incorporation) *in vivo*.^43^ To tackle this observation of such “nonspecific radioactivity” in blood, without specifying its origins, a two-stage approach has been explored [Figure 12]. Specifically, following administration of monoclonal Ab (mAb) labeled with transcyclooctynes to mice (here to target tumors), a second step was employed that utilized synthetically galactosylated (via 2-imino-2-methyoxyethyl (IME) chemistry) and tetrazine-modified (via NHS esters) albumin. This second conjugate was designed to clear blood-associated mAb via reaction and then active galactose-dependent asialoglycoprotein-mediated uptake to liver. Subsequent use of ^177^Lu-DOTA-tetrazine allowed enhanced specificity in conjugation in targeted tumor tissue. Thus, this system represents an interesting selectivity manifold in which tripartitite tissuoselectivities are exploited: tumor via mAb and blood via albumin circulation (and a reduced ability to diffuse from vasculature).^76^

While this type of pretargeting approach (in which an auxiliary is prelocated for late-stage functionalization) circumvents endogenous selectivities, it nonetheless highlights a possible functional purpose and exploits differences in PK/PD properties of two different agents. These are also an inherent part of tissuoselectivity, *i.e.*, a radio-isotope in a small molecule is rapidly cleared, thereby separating it from a larger targeting moiety (*e.g.*, antibody) with a longer biodistribution equilibrium. The examples that are built upon use of implanted tumors, which occur prevalently in this area, are essentially a borderline example of tissuo- or celluloselectivity but are nonetheless illustrative of *in vivo* compatibility (if not of fully endogenous selectivity). Other examples that straddle this boundary are therefore covered in the section 4 below on celluloselectivity.

### 3.7 Physical Methods

It is perhaps also worth considering other more physical and even surgical modes of tissuoselectivity. Powerful molecular selectivity has long been achieved using physical methods such as microinjection, for example, by combining so-called “misacylated” tRNAs as injected reagents with use of stop codon suppression to allow photocontrol mediated by incorporated uAAs.^77,78^ While this Review is deliberately chemical in its analysis, the application of chemical technologies in living systems will undoubtedly also take advantage of physical procedures beyond simple injection.

**Figure 13:**
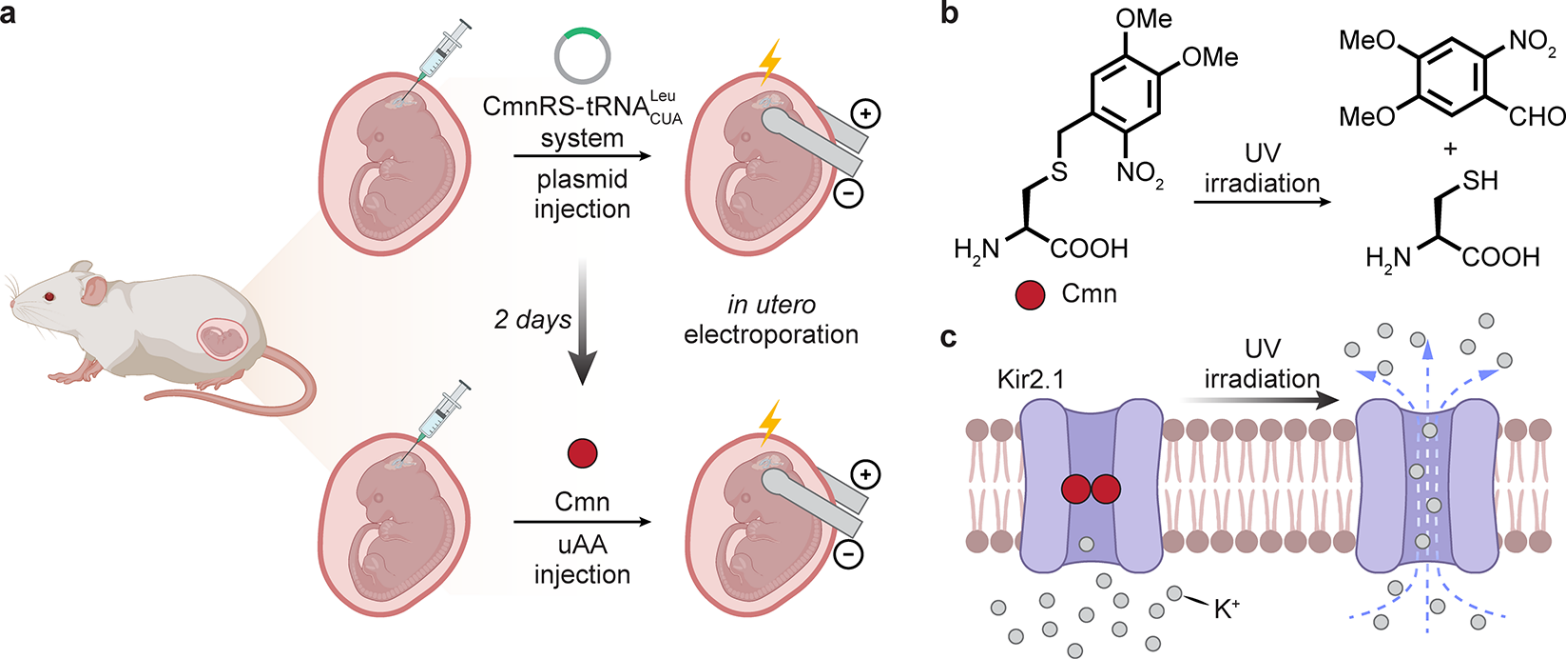
Demonstration of *in utero* electroporation and subsequent UAA incorporation for the modulation of rat cortical neurons.^80^

In one illustration, the use of electroporation to direct usefully selective activities toward tissue types can be considered [Figure 13]. For instance, *in utero* electroporation^79^ has allowed delivery of relevant genes (amino acid-tRNA synthetase and “stopped” protein-of-interest (POI) gene) for the use of stop-codon suppression in a mouse brain, applied to the potassium channel Kir2.1.^80^ Here, use of 4,5-dimethoxy-2-nitrobenzyl-cysteine (Cmn) as the suppressing uAA blocked potassium current under physiological conditions. Upon exposure to ultraviolet (UV) light, the C–S bond within Cmn is cleaved in Kir2.1, leading to the restoration of outward K^+^ current and reduction of membrane excitability not only in model cellular systems (rat hippocampal primary neurons) but also, through the use of prior electroporation, in a living mouse neocortex, where a light-activated K^+^ current in cortical neurons was observed.

**Figure 14:**
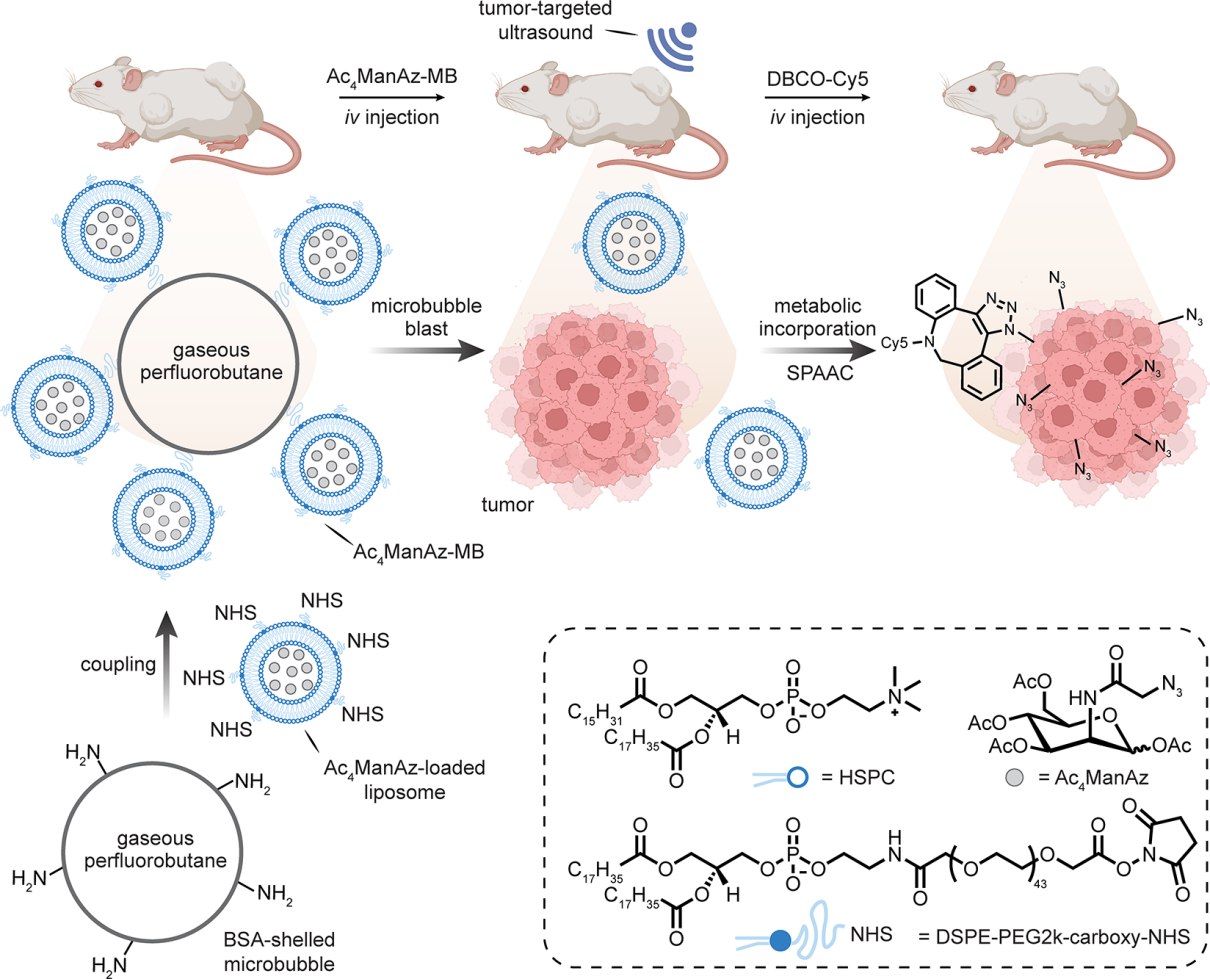
Bursting of “microbubbles” through ultrasonication as a suggested mode of tumor-selective metabolic incorporation.^81,82^ Abbreviations are as follows: MB = microbubble, BSA = bovine serum albumin, and HSPC = hydrogenated l-α-phosphatidylcholine.

Selectivity has also been apparently achieved by ultrasound “bursting” of so-called microbubbles. It is suggested that ManAz can be encapsulated in liposomes conjugated to microbubbles using a hierarchical assembly method. Thus, liposomes encapsulating ManAz and bearing NHS-activated carboxylates (via the use of 1,2-distearoyl-*sn*-glycero-3-phosphoethanolamine (DSPE)-PEG2k-carboxy-NHS) were generated. Amine “microbubbles” were synthesized through the sonication of decafluorobutane-saturated solutions of serum albumin and dextrose [Figure 14].^81^ These were then conjugated to each other via incubation, and the conjugate-microbubbles were allowed to separate from the rest of the mixture based on size/emulsion. These “emulsion-selected” systems were then given to mice, and subsequently explanted tumors were “treated” with ultrasound, which resulted in proposed disruption of the microbubbles. Subsequent intravenous (*iv*) injection of a DBCO-Cy5 label resulted in selective reaction only at the tumor tissue that was treated with ultrasound, suggesting ultrasound-dependent local selectivity.^82^ At some level, these complex systems may suffer from the subtleties of not only expertise in reproducibility but also layered contributions from underlying effects (*e.g.*, localized permeabilization by ultrasound-induced cavitation^83^). Nonetheless, it cannot be discounted that these may also exploit intriguing additional additive effects associated with selectivity engendered by such multicomponent systems.

## 4 Celluloselectivity

It is trite to consider tissues as merely collections of different cell types, but it is true that sufficient prevalence of a cell-type within a tissue can convey useful selectivity. The ability to target a specific cell type over another finds significant utility within the blood, and these form the bulk of examples here. This exploits deep knowledge of different lineages of blood cells with distinct repertoires of cell surface markers. These are often key to their native context and have long been exploited in a selective manner for biological and even immunological applications. Indeed, this knowledge has found broad clinical relevance particularly in oncology in the context of blood-based cancers, *e.g.*, treatments for leukemia.^84^ The ability to selectively block or enhance interactions between two different cell types has led to great advances in immunotherapeutic approaches for many cancers. Highly selective cell-specific targeting is the basis, for example, of chimeric antigen receptor (CAR) T-cell therapies, which exploit on one level artificially installed pathways to exploit endogenous celluloselectivities to induce desired cell-killing.^85^

Celluloselectivity is, as for tissuoselectivity, likely to exploit cell-associated traits that include cell-surface “biomarkers” and cell-specific uptake (and recycling) and/or metabolisms. As we note elsewhere, bathing just one cell type in a reagent is not celluloselectivity as defined in this Review, but we acknowledge that it is a common approach to developing cell-associated chemistries, can usefully test compatibilities, and can be a starting point for lower levels of selectivity of course (organelle, biomolecule–substrate, *etc.*). As for other parts of the spectrum of *in vivo* selectivity, we suggest here that there is therefore some straddling of strategic boundaries. For celluloselectivity, the cell surface may be considered in one sense as a gateway to selectivity; it may also be considered a distinct cellular region in the context of organelloselectivity and so will also be covered by some examples later in section 5.

**Figure 15:**
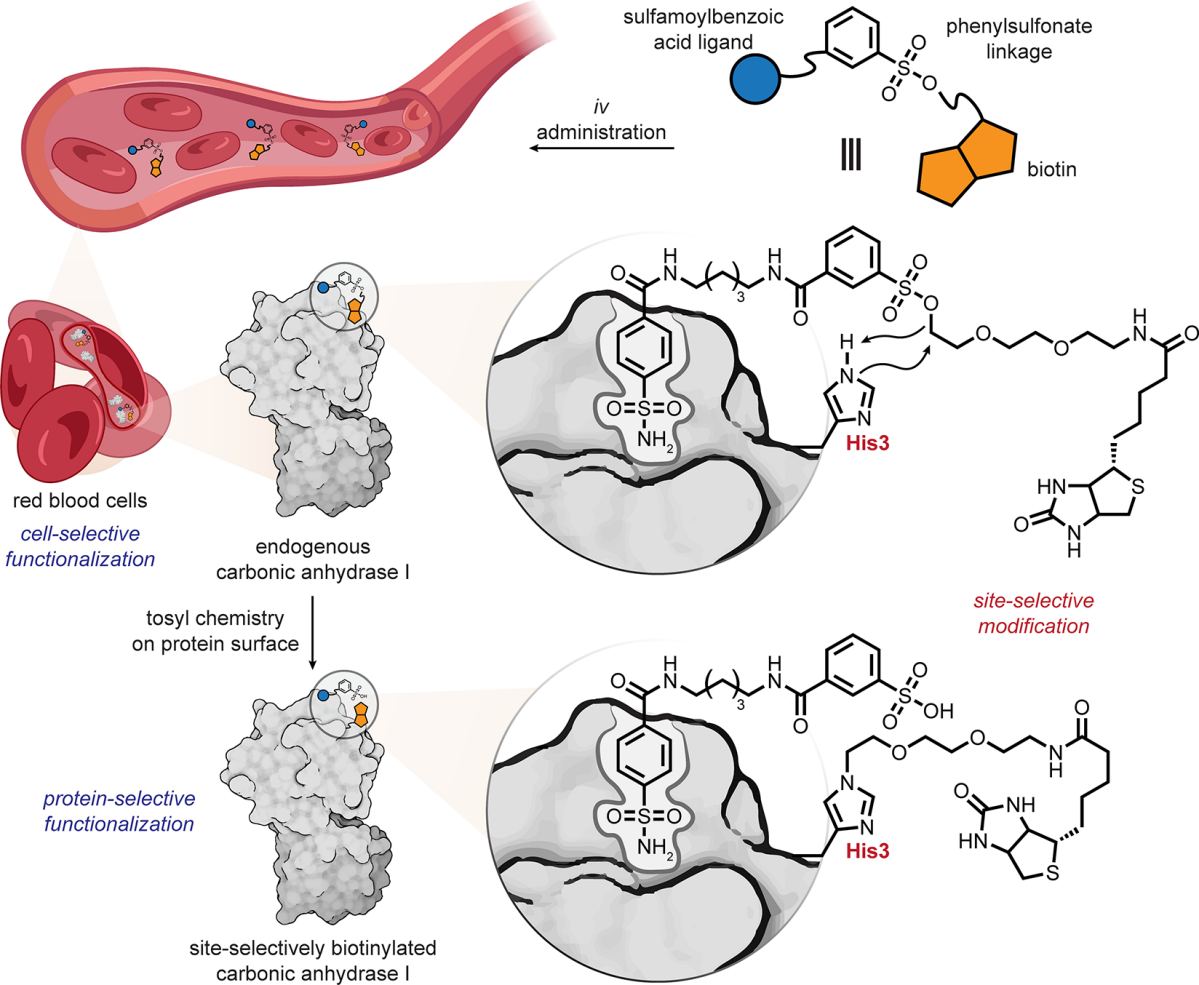
Red blood cell ligand-directed arylsulfonate chemistries.^87^

The factors that may lead to celluloselectivity may include a highly expressed or associated biomolecule that may be targeted by biomolecule-specific chemistries (see section 6). The cell viewed as a target is then characterized (as in all areas of selectivity) by the potential for layered selectivity. An example of red blood cell targeting is illustrative. Within blood as a tissue, red blood cells are distinguished by an abundance of cytosolic carbonic anhydrase (CA), as well as a permeability that allows for small-molecule access. While noncovalently directed (including recently termed proximity-directed methods) chemistries are seeing a current resurgeance, Hamachi’s ligand-directed chemistries^86^ have long shown utility in biomolecule–substrate selectivity (see also below). When applied to CA in blood as a tissue, this can further allow celluloselectivity. Thus, the use of a benzylsulfonamide moiety as a CA-selective ligand when conjugated to both a label (fluorescent, biotin, *etc.*) and a tuned electrophilic moiety (*e.g.*, arylsulfonyl) allowed targeting by the application of simple S_N_2 chemistries *in vivo*, enhanced by proximity [Figure 15].^87^*Iv* administration of a biotin-containing sulfonamide-arylsulfonate into mouse, followed by removal of blood, and analysis by Western blot revealed red blood cell-selective (and indeed CA-selective) biotinylation.

In addition to selectivity that is mediated by intracellular targets, abundant extracellular markers are perhaps even more obviously tractable. Here again, protein–target binding is often exploited but mediated typically by either Ab–protein interfaces or, more rarely, non-Ab–protein interfaces. Subsequent cell-selective reactions may then be engendered chemically or indeed biocatalytically. For example, anti-Her2-sialidase conjugates^88,89^ were used to selectively drive antitumor immune responses via the sialic acid-binding immunoglobulin-type lectin (Siglec)-E that is present on tumor-infiltrating myeloid cells. Notably, in this case, the choice of sialidase with appropriate kinetic parameters was suggested to be critical to avoiding off target activities.

**Figure 16:**
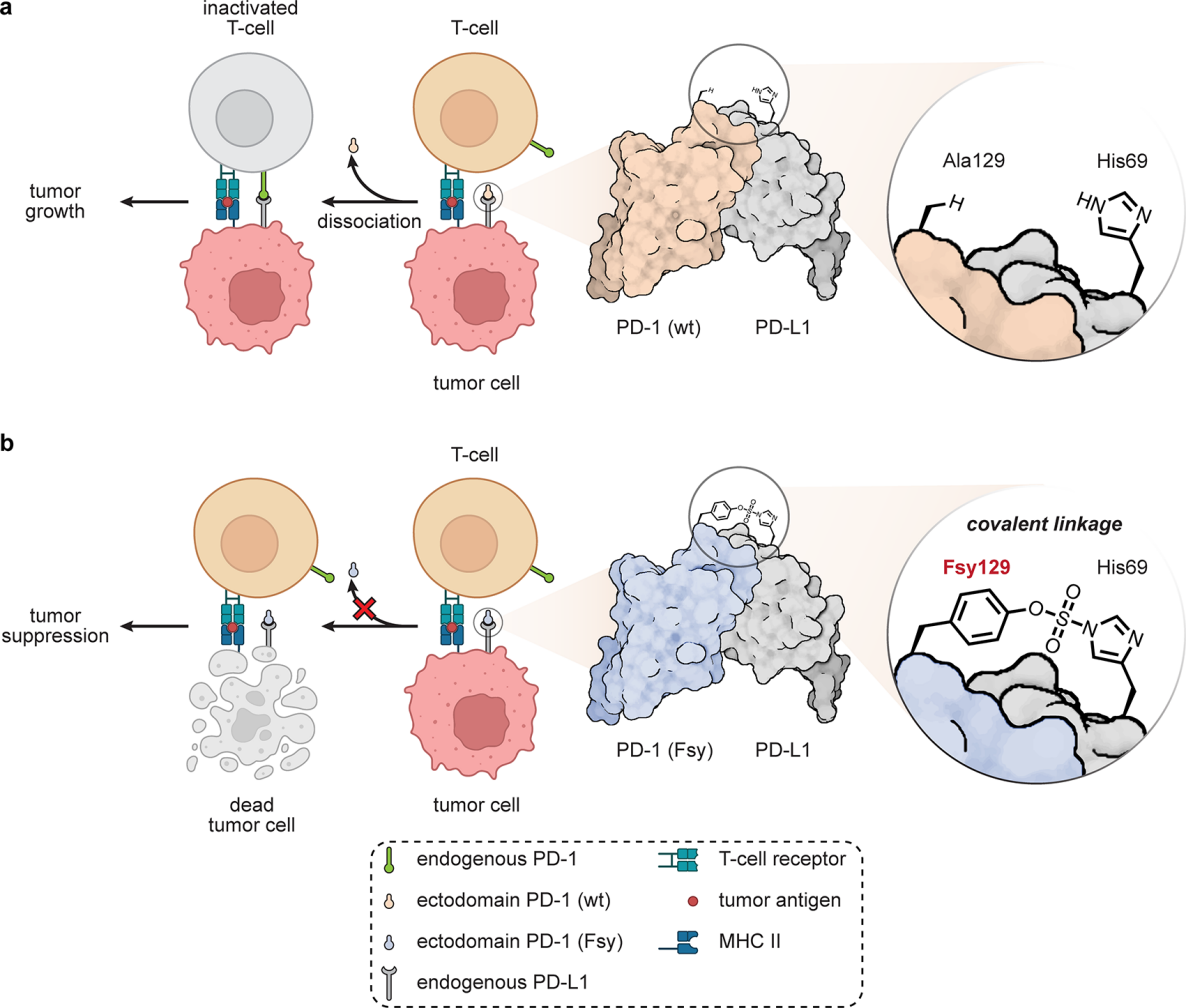
Potential adaptation of PD-1–PD-L1 binding for cell-targeted chemistries.^90^ (a) The exogenous WT-PD-1 ectodomain competes with endogenous membrane bound PD-1 on T-cells, but does not inhibit tumor growth. (b) Installation of a reactive Fsy uAA near its PPI allows the use of an exogenously added PD-1 variant that can intervene via covalent bond formation between PD-1 and PD-L1, leading to tumor suppression.

Non-Ab protein–protein interfaces (PPIs) can also be considered. In an instance of one type of “covalent protein inhibitor” (see also below), as proteins that possess uAAs bearing reactive functional groups that may trap partners, the installation of uAA fluorosulfate-tyrosine (Fsy) into programmed cell death protein 1 (PD-1) has created a very interesting example [Figure 16].^90^ Using amber codon suppression to site-selectively place this into the PD-1 ectodomain created a reactive protein selective for ligand PD-L1. PD-1–PD-L1 interaction is a key part of the immune checkpoint axis in immune signaling responsible for attenuating T-lymphocyte proliferation, release of cytokines, and cytotoxicity. The resulting dampened immune response is exploited by some tumors overexpressing PD-L1. Fsy-bearing PD-1 reacted and trapped PD-L1 *in vitro*; in tumor models in humanized mice, this synthetic protein inhibited tumor growth to the same extent as the clinically approved anti-PD-L1 antibody atezolizumab, which engages noncovalently. Nonreactive wild-type (wt)-PD-1 showed only minimal tumor growth inhibition, suggesting that *in vivo* celluloselectivity was important for functional activity.

Even nonproteinaceous interfaces may be considered for affinity-mediated chemistries to drive celluloselectivity. In an early example of targeted protein degradation/affinity-proteolysis (see section 6.5), celluloselective targeting of sugar-specific adhesins in bacterial cocultures exploited synthetically glycosylated proteases to catalyze LSF.^91^ Gal-terminated glycodendrimeric motifs were proposed as mimics of *N*-linked-glycans able to engage and erode cell-surface fimbrial Gal-receptors on human pathogen *Actinomyces naeslundii* needed for coculture and pathogenicity. Dose–response analyses revealed nanomolar inhibition (IC_50_ = 20 nM) of coaggregation with copathogen *Streptococcus oralis* substantially more potent than that of lactose as a small-molecule equivalent inhibitor (IC_50_ = 33 mM).

**Figure 17:**
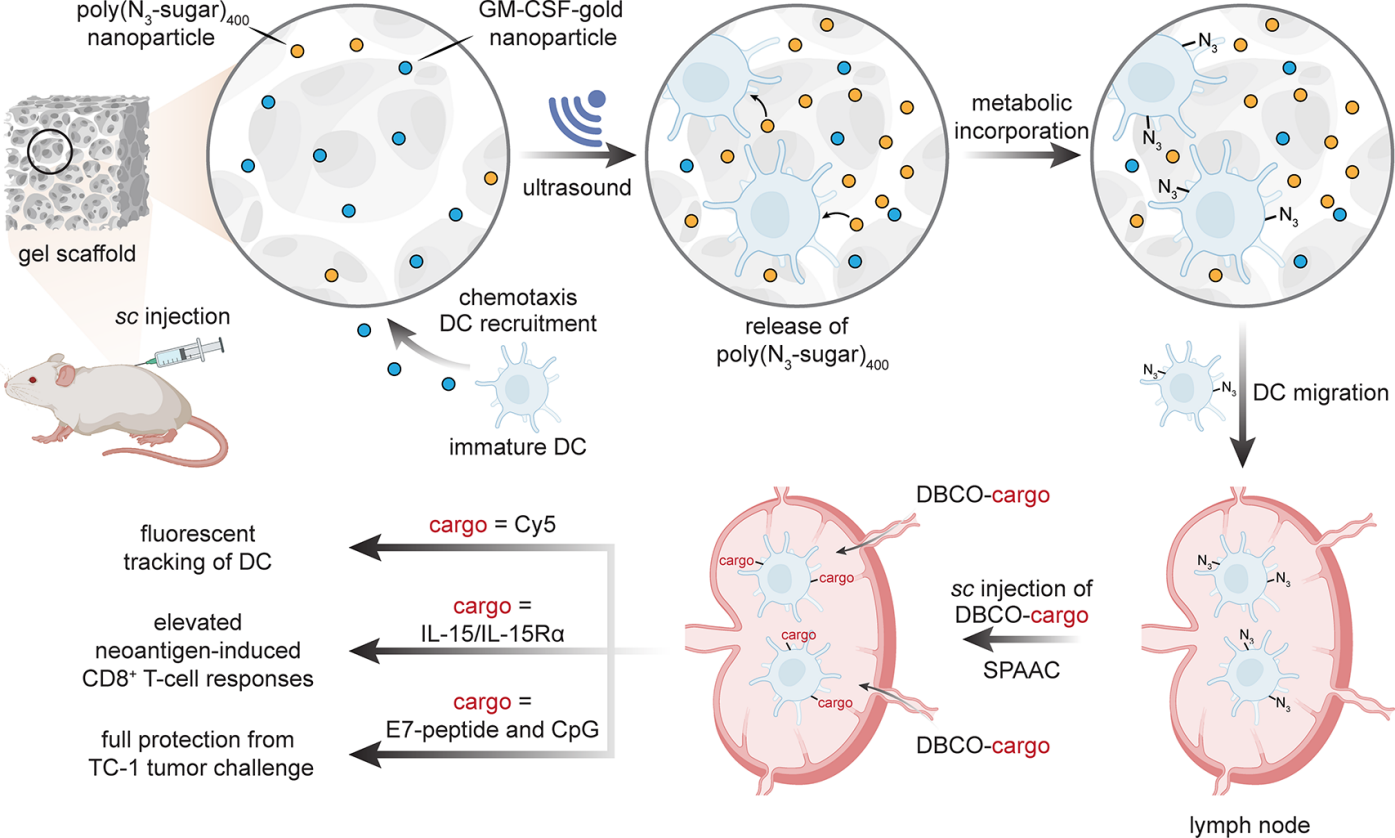
A “corralled herd” of dendritic cells can be labeled through metabolic incorporation of unnatural sugars. This allowed DCs to be selectively modified by SPAAC and tracked via fluorescence, with suggested modulation of immune response.^92,93^ s.c. = subcutaneous.

Chemotaxis and chemokinesis are other celluloselective traits that may be exploited. Dendritic cells (DCs) play critical roles in the adaptive immune processes that capture, process, and present antigens on the cell surface to T-cells and induce their polarization into effector cells. They show chemotaxis toward small glycoprotein granulocyte-macrophage colony stimulating factor (GM-CSF) as a potent inducer of DC differentiation, proliferation, and migration. Injectable macroporous alginate hydrogels loaded with GM-CSF in various ways can thus be employed as physical supports for the infiltration of DCs, thereby “herding” them.^92^ Combined placement within the hydrogel of polyacrylate copolymer nanoparticles (based on a polyacrylate of azido-sugar Ac_4_ManAz [poly(azido-sugar)_*n*_ (*n* = 25 or 400)]) followed by ultrasound has been proposed^93^ to allow release and then celluloselective metabolic labeling of the “herd” of DCs [Figure 17]. In this way, DCs were highly concentrated to gel injection sites within three days, compared to other immune cells (including neutrophils and macrophages), and then reacted. Subsequent SPAAC with various *iv*-administered DBCO-bearing conjugates allowed labeling of DCs (via a DBCO-Cy5 conjugate). These were then tracked to lymph nodes and allowed the creation of cytokine conjugates (via DBCO-IL-15/IL-15Rα) that appear to improve vaccine-induced neoantigen-specific CD8^+^ T-cell responses. In a dual DBCO-adjuvant/antigen approach, combined administration of DBCO-CpG with DBCO-E7-peptide (derived from human papillomavirus (HPV) E7 oncoprotein) also led to higher numbers of E7-tetramer^+^ CD8^+^ T-cells and interferon (IFN)-γ^+^ CD8^+^ T-cells with apparently full protection from an E7-expressing TC-1 tumor challenge in a prophylactic study. The seemingly striking effects here of inducing eventual celluloselectivity through physical location in an artificial cellular “corral” (where cells are then locally “bathed” in a metabolic labeling precursor) highlights an intriguing additional mode of selectivity.

### 4.1 Celluloselectivity through Genetic Control

Genetic strategies to imbue novel cellular selectivity can also exploit semiclassical and powerful strategies in animal genetics. Two strong examples, while not employing *in vivo* LSF chemistry, highlight the potential.

Use of an AAV-based vector under the control of a cell-type specific promoter can allow celluloselective expression of unnatural amino-acid biosynthetic machinery (*i.e.*, tRNA–tRNA synthetase pair). For example, application in wild-type mice has allowed neuronal-over-glial cell selectivity using an mCherry-P2A-*Mm*PylS genetic fusion under the control of synapsin 1 promotor in brain tissue for the incorporation of an alkyne-containing protected lysine uAA variant (AlkK) into proteins.^94^ Rather than suppress stop/amber codons, the tRNA in this case competed for a native sense codon (CAU), leading to stochastic incorporation of the unnatural amino acid in competition with His throughout the proteome.

In transgenic mice, semiclassical Cre-LoxP control can also be exploited. By crossing a mouse line that bears a “floxed-STOP” version of a methionine tRNA synthetase that carries a point mutant (L247G, which allows incorporation of azidonorleucine (Anl) at Met sites, MetRS*) with two different Cre-driver lines, celluloselectivity for Anl was enabled.^95^ Thus, glutamatergic excitatory neuron-selective (via CaMK2a-Cre) and GABAergic inhibitory neuron-selective (via glutamic acid decarboxylase (GAD) 2-Cre) expression of the mutant-MetRS under the control of Cre recombinase enabled proteome-wide celluloselective uAA Anl incorporation. Mice were fed uAA Anl simply in their drinking water. After brains had been harvested, *ex vivo* CuAAC allowed fluorescent labeling and covalent retrieval for chemoproteomic analyses. An interesting experiment where mice were exposed to either normal or enriched sensory environments allowed the identification of >200 proteins that were concurrently significantly up- or down-regulated.

### 4.2 Celluloselective Metabolic Labeling

The ability to prime the molecular substrates that are available via feeding with unnatural analogues in a cell is a now widespread approach (sections 2 and 3.5). Through selective pressure and/or use of depleted media, the bathing of a cell may permit the uptake of unnatural glycans (uGs) and unnatural amino acids (uAAs). This does not exploit *per se* any particular mode of celluloselectivity yet provides a useful means for placing potentially selectively reactive functionality into living systems. Met analogues allow exploitation of the plasticity^96^ of MetRS. This has been demonstrated in various strategies, including in chemical proteomics to provide a reactive tag in proteomes through low-level global labeling via methods such as BONCAT^97^ and QuanCAT^98^ that exploit selective reactions for retrieval, typically from cellular lysate. It may be directed toward certain cell types, even primary cells.^98^ For example, when neuronal cells were deprived of methionine for 30 min before being incubated with Aha for 2–4 h, resulting incorporation of Aha into newly synthesized proteins allowed cell-surface trafficking to be observed using a difluorocyclooctyne (DIFO)-biotin reagent via SPAAC due to its cell-surface impermeability. A quantum dot labeled streptavidin was then used to visualize these newly generated proteins and track their movement on the cell surface.^99^ However, its exploitation in cell-selective methods is more rare and can provide an additional selectivity filter (see also the discussion under tissues).

**Figure 18:**
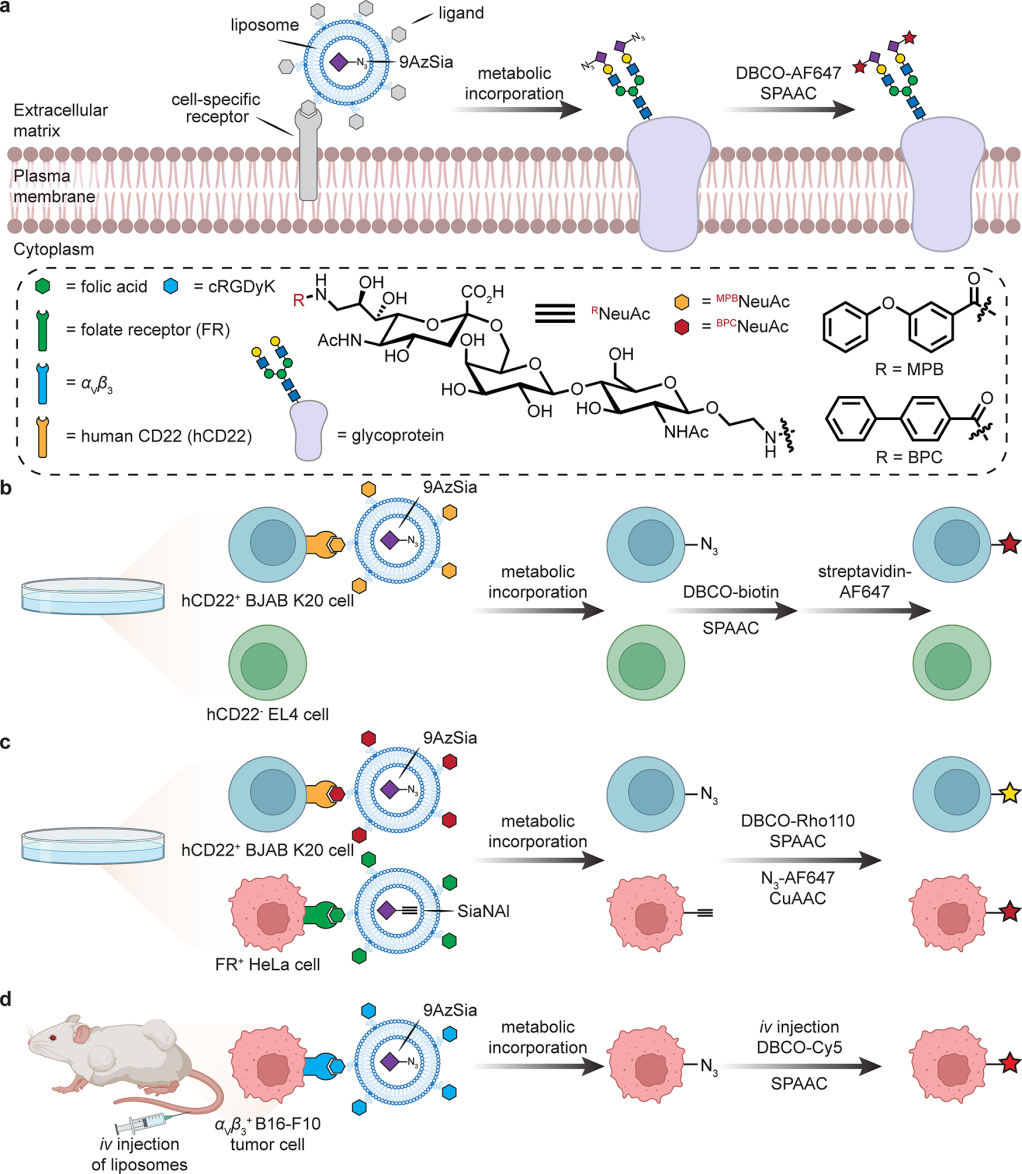
Cell-targeted metabolic labeling. The generation of liposomes displaying cell-specific targeting ligands and encapsulating modified sialic acid derivatives allows cell-type-specific uptake and display of the modified glycan.^101,102^ SiaNAl = *N*-(4-pentynoyl)neuramic acid.

Liposomal encapsulation can allow cellular access, and several targeted methods have been suggested [Figure 18]. Folate receptor (FR)-targeted liposomes (f-LPs)^100^ encapsulating 9-azido sialic acid (9AzSia) were internalized into endosomes and lysosomes successively via FR-mediated endocytosis. Here, rather than a ManNAc-derived Sia precursor, sialin is instead invoked as a pathway for the metabolic incorporation of 9AzSia into cell-surface glycans after initial FR-mediated uptake release and transport to cytosol by a lysosomal sugar transporter. This liposome-assisted strategy has been expanded to various ligand–receptor pairs to target given cell types.^101^ For instance, 9-*N*-*m*-phenoxybenzamido-NeuAcα2,6Galβ1,4GlcNAc (^MPB^NeuAc) is a synthetic glycan ligand of human CD22 (Siglec-2) specifically expressed on B-lymphocytes. 9AzSia was encapsulated in ^MPB^NeuAc-modified LPs and cocultured with CD22^+^ human B-cell BJAB K20 and CD22^–^ murine T-cell EL4 lines. After successive treatment with DBCO-biotin and streptavidin-AF647, a significantly selective labeling of K20 cells over EL4 was observed. Interestingly, this strategy could be further applied in a multiplexed labeling system. In this case, another synthetic glycan ligand of CD22, 9-*N*-biphenylcarboxyl-NeuAc-α-2,6-Gal-β-1,4-GlcNAc (^BPC^NeuAc), was utilized for targeting K20 cells, while f-LPs were employed for targeting FR^+^ HeLa cells. By encapsulating azido- and alkynyl-modified Sia, respectively, the corresponding LPs were able to selectively label K20 and FR^+^ HeLa cells in a dual coculture experiment. Similarly, cyclic Arg-Gly-Asp-d-Tyr-Lys (cRGDyK) pentapeptide has been suggested as a targeting ligand for recognition by integrin α_V_β_3_, which is overexpressed on certain tumor cells B16–F10. In mice, cRGDyK-9AzSia liposomes targeted B16–F10 tumor tissue after *iv* administration, followed by chased labeling with DBCO-Cy5. In the absence of the cRGDyK targeting peptide on the liposomes, the tumor tissue was still labeled but at a lower level; notably, several other organs were also labeled (*e.g.*, through renal clearance and accumulation of the liposomes in the liver and spleen).^102^

## 5 Organelloselectivity

Selective observation of and functionalization in different organelles has long been investigated, with a number of key selectivity modes or “drivers” often invoked [Figure 20]. Typically this involves installing targeting moieties into one of the reaction components to localize them to a specific organelle. There is also somewhat of an inherent circularity in these studies in that it is the localization of a given marker or dye that is itself sometimes used to characterize or even define a given organelle. The emergence of membraneless organelles/lipid droplets (LDs)/stress granules^103−107^ further challenges definitions. There is the general additional limitation that in many cases protein tags used for localization/location biology via microscopy may be at best correlative or fleeting in some cases.

**Figure 19:**
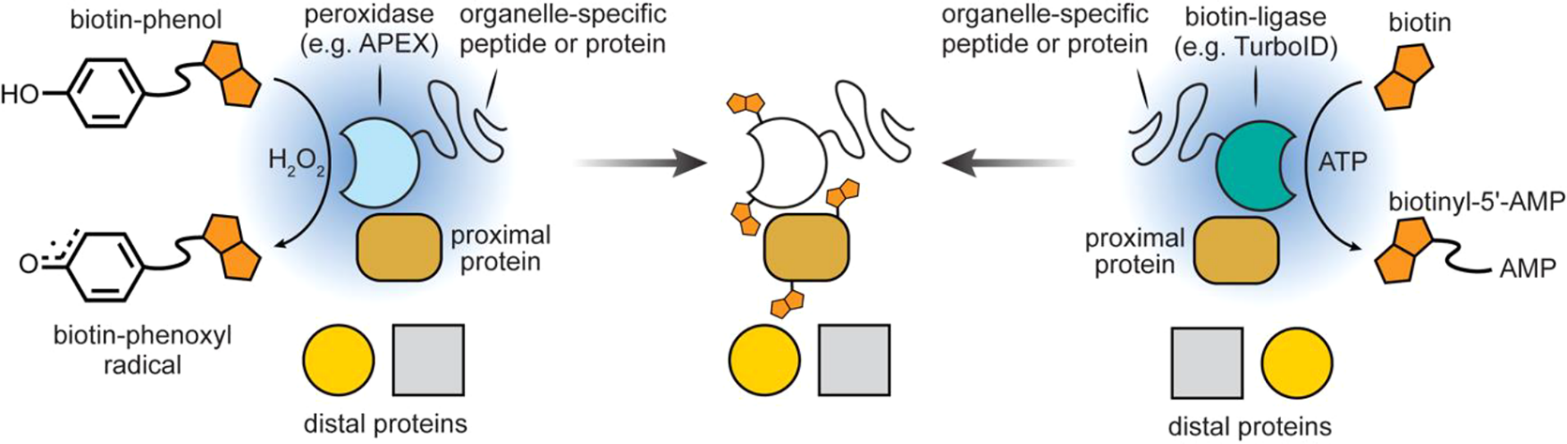
APEX and turboID allow for the labeling of proteins that are proximal to the target.^111−116^

**Figure 20:**
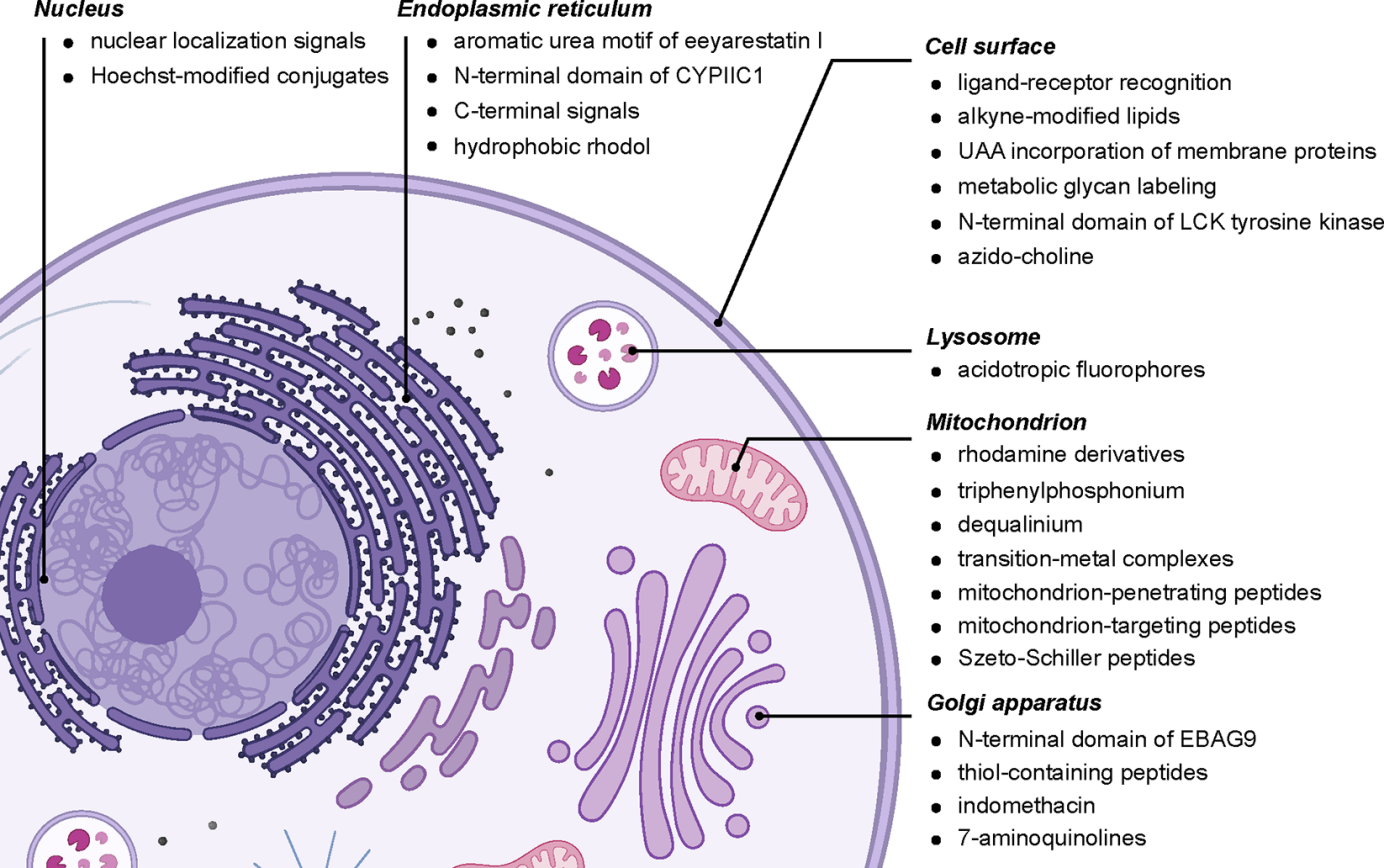
A selected summary of diverse organelle-directing motifs. Diverse targeting methods of differing provenance exist for the following: the nucleus,^48,119−122^ including those based on classical Hoechst “stain”;^123^ the ER via C-terminal signal KDEL,^124^ eeyarestatin,^125^ N-terminal signal CYPIIC1,^126^ or others;^127^ the cell surface via localization signals (*e.g.*, LCK_1–10_^128^), biomarker binding (*e.g.*, “covalent” aptamers^129^ or lectins^130^), or metabolically labeled residues (*e.g.*, glycans^131^ and lipids^132,133^); the lysosome;^134,135^ the mitochondrion^136,137^ via aryl phosphoniums,^138,139^ quinoliniums,^140^ rhodamines,^141−143^ metal complexes (*e.g.*, suggested gold,^144^ platinum,^145^ ruthenium,^146^ and iridium^147^) and targeting peptides;^136,148−150^ and Golgi (*e.g.*, EBAG9,^151^ suggested thiopeptides,^152−154^ and suggested aminoquinolines^155^).

Targeting often exploits the differential physicochemical environments present in different organelles. As part of the compartmentalization of life, specific organelles have evolved to carry out specific functions, requiring an often tightly controlled set of chemical (pH or redox potential) conditions in some cases.^108^ For example, the pH of certain organelles is essential for their function.^109^ While the cytoplasm is pH ∼ 7.2, the lysosome maintains a strongly acidic pH = 4.5–5.0, with finely tuned proteases operating optimally in this pH regime. Mitochondria, on the other hand, operate at pH ∼ 8.0 due to the proton gradient required to drive ATP generation. As another example, peroxisomes play a highly compartmentalized and specific role in a number of cellular processes, mainly through oxygen metabolism and generation of reactive oxygen species such as peroxide and superoxide anions.^110^ The maintenance of imbalances in pH and redox potential is therefore a dominant global feature of the function of such boundaries in living organisms that necessitate far-from-equilibrium conditions.

The proposed evolutionary origin of mitochondria as an engulfed prokaryote, as well as its maintained minimal genome, highlights it as almost a pseudo-organism within living cells, and this adds a further interesting layer to our considerations of selectivity (pseudo-organism targeting).

Classical genetic methods provide immediate organelle-targeting scope through the use of appropriate localization sequences. In this way, powerful engineered ascorbate peroxidase (APEX) systems, for example, can be directed toward the mitochondria, cytosol, nucleus, endoplasmic reticulum (ER), and cell surface, among others [Figure 19]. The short lifetime, diffusion-limited labeling radius, and relative membrane-impermeability of the generated phenoxyl radicals endow organelle-localized localization and reaction with organelle-specific proteins.^111,112^ By injecting an APEX-encoding plasmid with different organelle-specific signaling peptides into embryos of flies, this approach has been extended to live *Drosophila* tissues upon treatment of biotin-phenol and H_2_O_2_ in dissected tissues (muscle cells, imaginal discs, and salivary gland).^113^ APEX anchored to lipid droplets (LDs), by fusing from the C-terminus of the perilipin family member PLIN2, has also allowed targeting of LD proteins in noncanonical “organelles” in living cells.^114^ The APEX system requires exogenous H_2_O_2_, which can be toxic to living samples and may cause artifacts with redox-sensitive proteins. As an alternative, the exploitation of biotin ligase (BirA) allows local labeling with biotin, so-called bioID.^115^ Directed evolution has created a BirA-variant with more rapid (10 min) response (so-called “turboID”) to exogenous biotin that, again, may be directed with different organelle-specific signaling sequences.^116^

Some simple additional chemical principles can be explored. For example, physicochemical modes coupled with a proposed form of dynamic combinatorial optimization are invoked^117^ for some hydrazide organelle pretargeting reagents (mainly supported by initial microscopic studies) generated to target lipid microdroplets (bisoctyl moiety), membranes (dodecyl sulfonate), and mitochondria (triphenylphosphonium). Although not fully demonstrated beyond apparent reversibility, the authors argued that this allowed the reaction of the ketone in fluorescent drug doxorubicin via ketone-hydrazone formation.

In other cases, organelle-specific metabolism may simply be exploited for metabolic labeling. As an early (and now widespread) example, alkynyl-deoxyuridine can be incorporated into DNA in the nucleus of live HeLa cells and then, without fixing, labeled via the CuAAC reaction with a fluorescent dye.^118^

### 5.1 Cell Surface/Pericellular Regions

**Figure 21:**
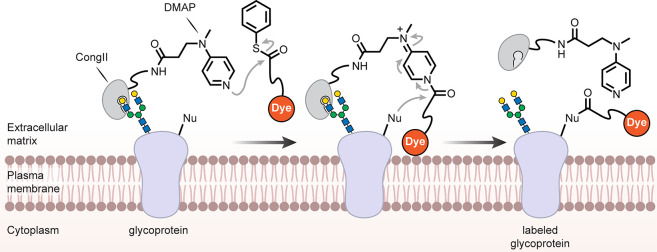
Semisynthetic lectin allows cell-surface-directed chemistries via glycocalyx binding.^130^

While lipidic interactions in plasma membranes are a logical target (see section 5.2), the often-extensive glycocalyx of many cells means that generalized membrane binding approaches may also usefully target cell-surface glycans. Broad lectins (for example, labeled wheat germ agglutinin, WGA) are routinely used reagents in microscopy; these principles have been elegantly extended to semisynthetic variants. Starting from conger eel galectin (CongII), a galactoside-binding lectin, one example has been generated bearing 4-dimethylaminopyridine (DMAP)-tethered moieties [Figure 21] to enable cell-surface catalysis. This modified CongII’s preparation exploits carbohydrate ligand-directed chemistries: sequential DMAP-catalyzed acylation via ligand-directed chemistry to introduce an alkyne and then CuAAC to add tethered DMAP. CongII bearing DMAPs then can be used for directed, selective chemistry on cell surfaces [Figure 21] by binding carbohydrates. Ingeniously, the initial use of the carbohydrate-binding site to direct chemistries proximal (but not into the binding site itself) during the creation of CongII then leaves this site free for mediating the later cell-surface directed chemistries. When cells (HeLa or COS7) were incubated with the CongII conjugate in the presence of fluorescent phenyl-thioesters, labeling of the cell surface was observed, which was dependent not only on the presence of the lectin but also on cell-surface glycosylation (removed by treatment with glycosidases). A number of modified cell-surface glycoproteins can be observed and identified via immunoblotting. Here the effective selectivity from an elegantly simple proximity-mediated activation of thioester by the DMAP-containing lectin (by proposed nucleophilic catalysis) highlights the likely power of densely arrayed targets and potentially multivalency (here displaying terminal galactose units).^130^

Metabolic incorporation approaches (see sections 2, 3.5, and 4.2) have widely exploited cell-surface glycosylation even in complex organisms.^66^ Dual variants prove possible; combined use of acetylated-mannose derivatives bearing terminal alkenes (for reaction with tetrazines via IEDDA, once incorporated into sialic acids displayed on *N*-glycans) and acetylated GalNAz (for reaction with DBCO, once incorporated into mucin type *O*-glycans) allowed two-color staining in HeLa cells. Interestingly, complementary distributions in distinct parts of cellular membranes could be observed.^131^

### 5.2 Plasma (and Other) Membranes

While *a priori* it may seem difficult to target one lipid bilayer over another,^156^ recent interesting fine-tuning of solvatochromic dye Nile Red suggests that some selectivity (*e.g.*, plasma membrane) via altered reversibility may be possible.^157^ In several of the examples that follow (not only in this section), the observed selectivity for certain organelles likely rests on aspects of the membrane that bounds a given organelle, at least in part.

**Figure 22:**
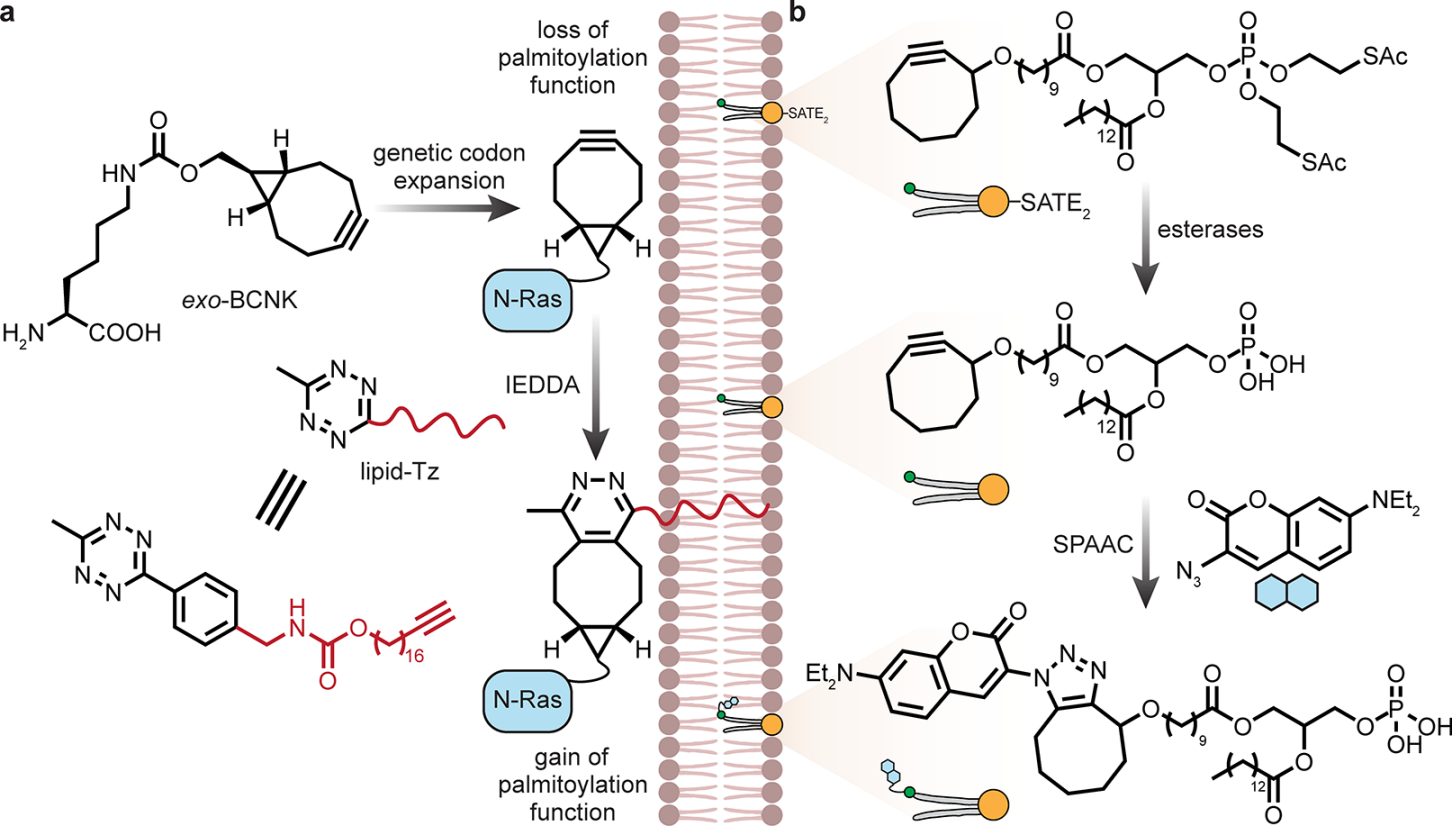
Unnatural lipids for membrane targeting. (a) Generation of a modified N-Ras protein bearing a lipid mimic via uAA incorporation results in membrane localization.^158^ (b) Insertion of a cycloalkyne into a lipid and subsequent coupling to a coumarin fluorophore probe allows lipid tracking.^132^

The lipid bilayer under the glycocalyx provides a target for potentially selective access to plasma membranes [Figure 22]. The inner plasma leaflet is the suggested site of a lipid-modified tetrazine used^158^ to drive the association of N-Ras with the plasma membrane. This was used to recapitulate aspects of the native Ras/ERK signaling pathway (increased phosphorylation of ERK observed only in the presence of the lipid-modified tetrazine). This system exploited the expression of N-Ras with a bicyclooctyne modified lysine (via amber codon suppression) at the typically modified cysteine site. Some caution should perhaps be applied to the interpreted reaction location in this case, however, since the increased membrane fraction of N-Ras could be driven by partitioning either pre- or post-IEDDA.

Metabolism can also be exploited to create unnatural lipids (uLs). Phosphatidic acid precursors (bearing *S*-acetylthioethoxy-protected head groups) have been designed on the simple but seemingly effective assumption that terminal alkynes, and impressively even cyclooctyne, can be carried with minimal perturbation at the terminus of fatty acid chains. In early examples applied to various cell types (including RAW macrophages),^132^ resulting membrane lipids could be labeled in cells after being fixed with an azido coumarin dye by CuAAC and also by direct treatment with a cyclooctyne variant. Fluorescence was distributed readily throughout cellular membranes, perhaps indicative of lipid movement in the cells.

Use of an alternative precursor, azido-choline, has provided a powerfully broad approach^133^ that has allowed both visualization and quantitative analysis of interorganelle lipid transport in live cells. Metabolic incorporation into choline-containing phospholipids (the major component of mammalian cell membranes) could then be partnered with different organelle-localizable DBCO derivatives for SPAAC (*e.g.*, Rhodol-DBCO for ER and Golgi apparatus; tetraethylrhodamine-DBCO for mitochondria). The authors elegantly demonstrated, in this way, that the autophagosomal membrane likely originates from the ER in a manner that moves past approaches that would have been merely correlative if explored with classical (*e.g.*, FP-based) approaches.

### 5.3 Mitochondria

**Figure 23:**
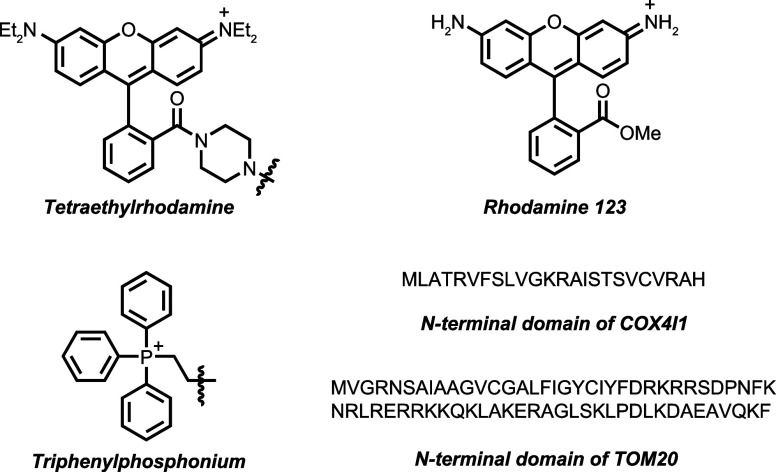
Example motifs used to target mitochondria.^159^ See also Figure 20.

Mitochondrial targeting has been driven frequently through the use of lipophilic cations; initial cell-permeability at the plasma membrane by virtue of an initial membrane potential (∼30–50 mV) thus leads to striking orders of magnitude concentration increases in mitochondria by virtue of an estimated up to 180 mV potential at the mitochondrial membrane. Motifs such as triphenylphosphonium^159^ or certain rhodamines are widely used in this context and can allow localization of a variety of reactive moieties (alkyl chloride, epoxide, thioester, *etc.*) for selective labeling of the mitochondrial proteome [Figure 23].^160^

Modulation of an *in situ* azide-DBCO reaction has been proposed as a measure of the mitochondrial membrane potential (driving uptake of attached phosphoniums in two respective precursors in line with the Nernst equation) *in vivo*. In mice, the heart showed a very high uptake of the two components and the SPAAC product was observed within 1 h after coinjection. The mitochondrial membrane potential could be altered either chemically via dinitrophenol or genetically through knockouts of various respiratory complex proteins; in that way, the levels of the corresponding product are significantly altered by virtue of second-order kinetics.^161^ This approach nicely extends simple chemical logic to relatively precise probing in not only an organelle-selective but also a tissue-selective (heart) manner.

By taking advantage of the localization of rhodamine 123 (Rh123) and proposed exploitation of its high triplet energy in specific energy-transfer-driven azide-to-nitrene transformation, the presence of excited dye has been explored in mitochondria.^162^ The wavelength of light used was also able to somewhat control organelle selectivity: a green LED (515 nm) resulted in excitation of the Rh123 and proximal activation of aryl azide to form reactive nitrene species and thus mitochondria-selective protein labeling, whereas UV irradiation (365 nm) resulted in direct UV-induced nitrene formation and thus nonspecific whole-cell protein labeling.

**Figure 24:**
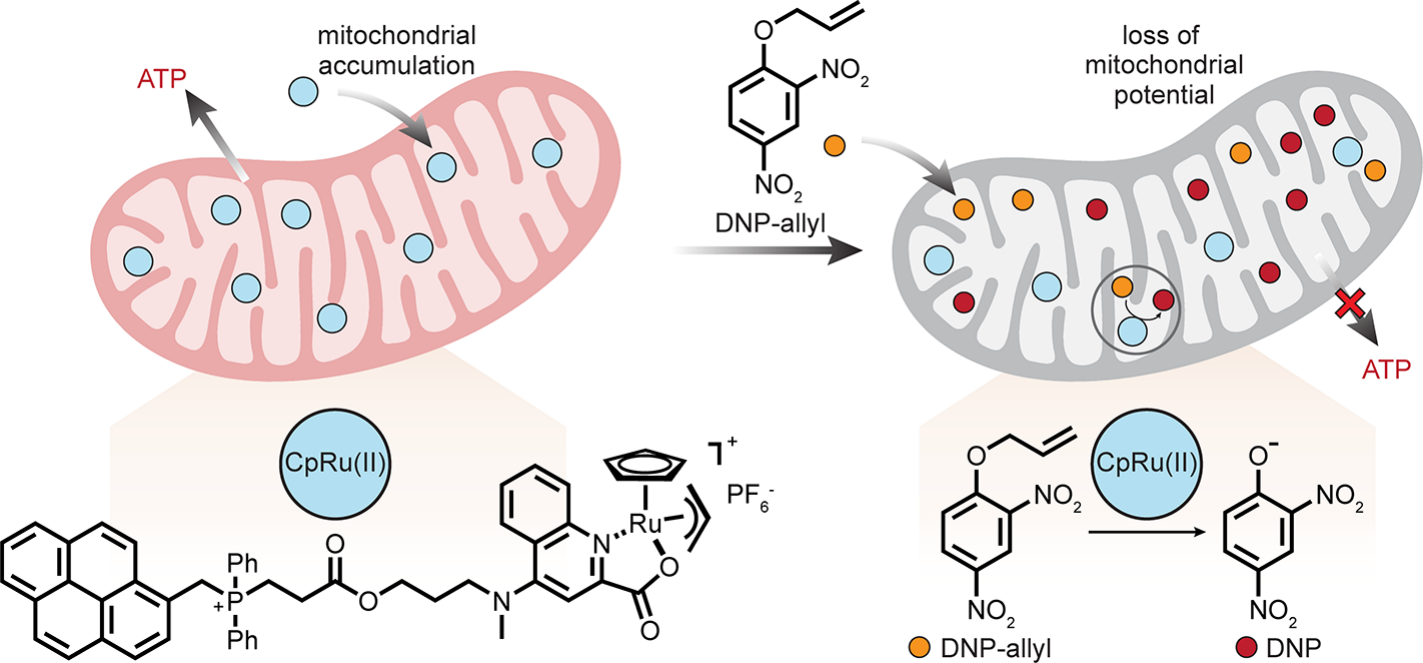
Localization of a ruthenium complex to the mitochondria allows deallylation and manipulation of mitochondrial potential.^163^ DNP = dinitrophenol.

The targeting of a CpRu(II) complex bearing a 2-quinoline carboxylate ligand via an appended triphenylphosphonium or diphenylpyrenephosphonium nicely enabled the uncaging of an allyl 2,4-dinitrophenol derivative. This occurred selectively in mitochondria, with a 15-fold accumulation in comparison with cytosol, as measured by inductively coupled plasma-mass spectrometry (ICP-MS)) [Figure 24]. The resulting Ru-dependent nitrophenol-mediated depolarization of mitochondria illustrated an elegant colocalization strategy for the selective manipulation of ATP production, potentially via transition-metal-mediated control.^163^

### 5.4 Lysosomes

**Figure 25:**
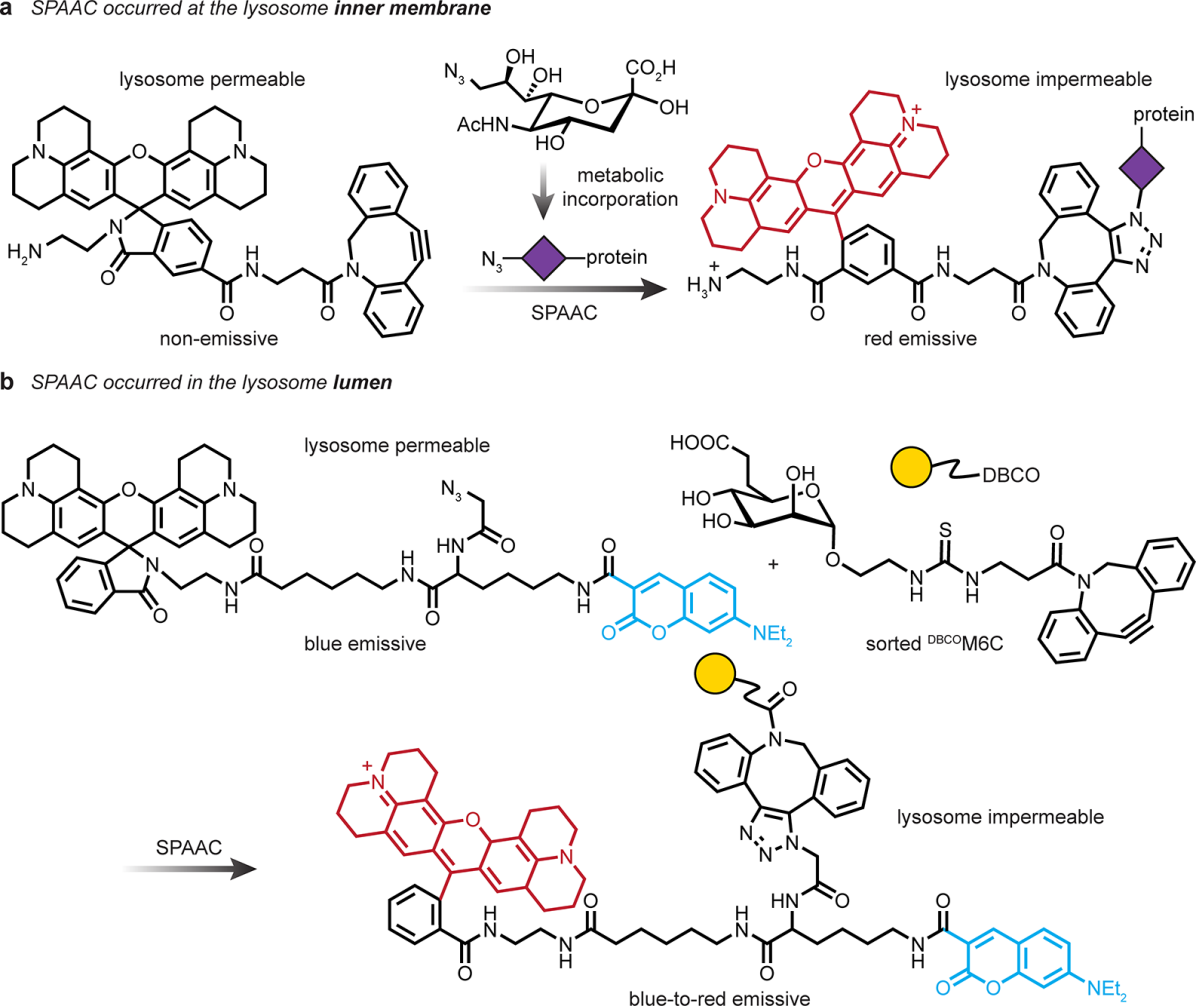
Intralysosomal targeting. (a) Inner membrane protein *N*-glycan labeling with a lysosome permeable dye.^164^ (b) Structure and reaction of a lyosomally accumulated sugar, 6-carboxy-mannoside (M6C).^165^

A proposed similarity of the inner membranes of lysosomes in their display of *N*-linked glycoprotein contents has been tested^164^ by a trick of metabolic incorporation similar to that used for cell surfaces. Use of acidotropic DBCOs to target localized azido-glycoproteins on the inner leaflet of lysosomes was suggested to overcome the typical dispersion of such dyes by a form of anchoring [Figure 25]. These probes showed selective uptake and preferential accumulation in the acidic lysosome driven by amine moieties. In cells treated with baflomycin A1, lysosomes are neutralized and such dyes typically dissipate from the lysosome. Use of direct 9-azido-Sia feeding followed by this form of “anchored SPAAC” is suggested, as it resulted in intralysosomal glycoproteins that showed apparent resistance to neutralization and did not dissipate compared to a “nonanchored” version. This, in turn, allowed for investigation of lysosome membrane permeabilization in different types of cell death pathways. A similar advantage of proposed sugar-sorting has been previously exploited in intralysosomal chemistries to trap a diffusible dye that was taken up by the well-known mannose-6-phosphate lysosomal trafficking pathway coupled with the use of 6-carboxy-mannosyl-DBCO [Figure 25].^165^

### 5.5 Other

**Figure 26:**
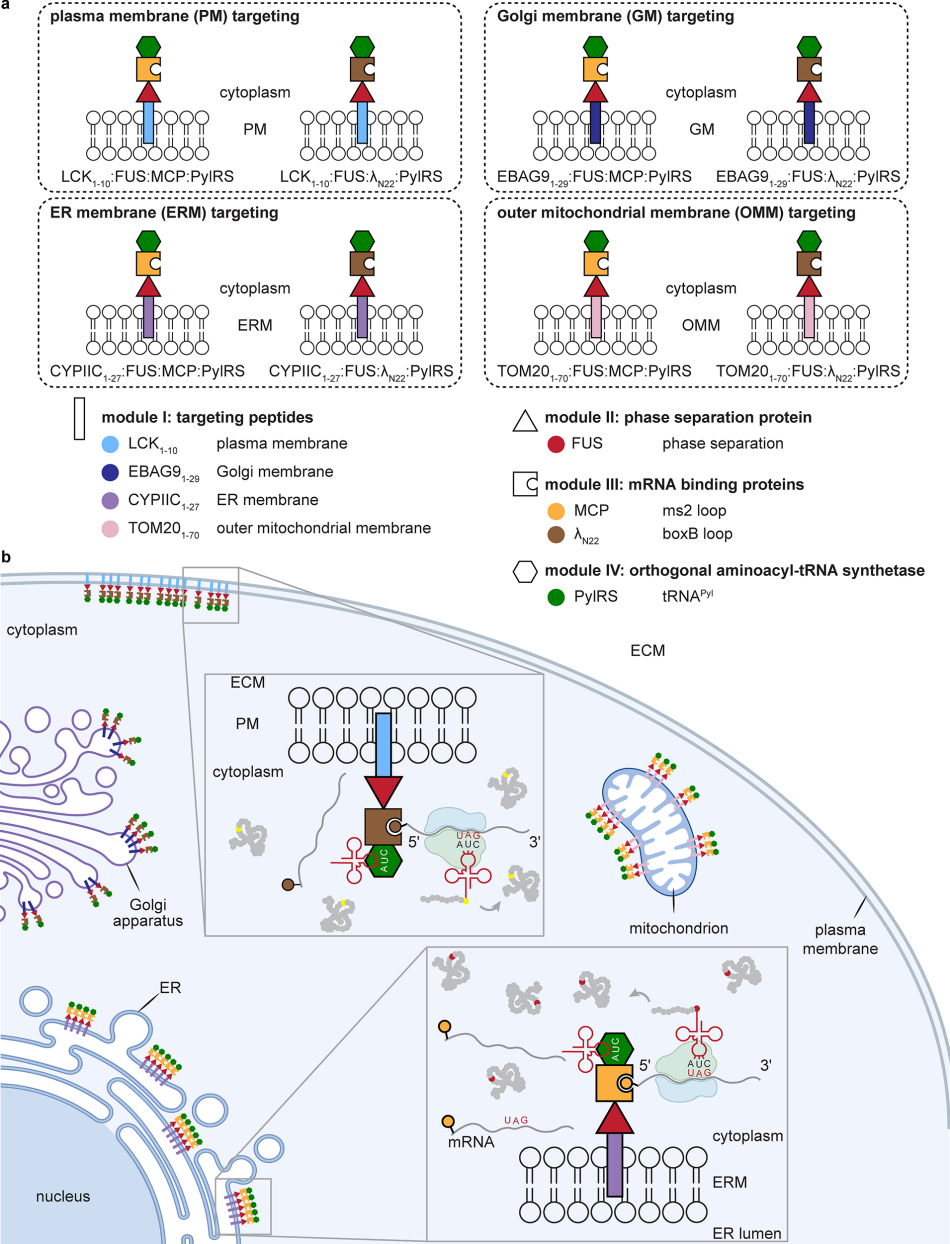
Exploiting pseudo-organelles.^166,167^ (a) Organelle-membrane-specific peptide tags were used to target mRNA binding proteins, phase separation proteins (to promote film formation), and orthogonal aaRSs to specific subcellular locations. (b) Multiple proteins bearing uAAs could be synthesized selectively at given locations within the cell. ECM = extracellular matrix.

Other broad approaches highlight the potential of organelles as essentially segregated localized systems within cells. A very interesting example of an engineered system has been used to install different noncanonical amino acids into proteins via amber stop codon suppression that succeeds by taking elegant advantage of designed enhanced local concentrations via scaffolding on organelles (here motif-tagged mRNA suppressing “charged” tRNA).^166^ In order to achieve this, fused-protein systems were constructed that generate so-called “film-like organelles” arranged/anchored on the cytoplasmic surfaces of various organelle membranes. Thus, four-module fusing of (i) a targeting peptide that anchors a desired protein into the specific membrane, (ii) a protein to induce a high local concentration via self-assembly and phase separation (*e.g.*, fused in sarcoma (FUS)), (iii) a specific orthogonal aaRS, and finally (iv) a specific mRNA-motif binding protein creates a pseudo-organelle for protein synthesis that is budded from the cytosolic side of the chosen membrane [Figure 26]. An mRNA for the POI is then used that also contains a specific mRNA motif to direct it to the RNA-binding protein module of the fusion protein located in the pseudo-organelle. Critical to this method is the fact that any mRNA in the cytoplasm will not fully read through (due to the stop codon); suppression happens only when there is a high effective/local concentration of charged orthogonal tRNA at the site of the “film-like organelle” on the membrane surface.^167^ Expressed POIs were then modified with IEDDA chemistry via noncanonical amino acids that were inserted. While in effect this latter chemistry was used for simple labelling in a nonselective manner, this work, to our mind, is highly prescient for its scaffolding of relevant biocatalytic activities (here protein synthesis) in a *de novo* localized organelle-like mode that is powerfully suggestive for exploitation in selective *in vivo* chemistries.

As well as genetic tags or physicochemical properties, organelle-associated catalysis may also be exploited for selective targeting in other ways. For example, an epoxomicin variant can act as a tagged covalent proteasome inhibitor that is “activity trapped” by its catalytic mechanism.^168^ Thus, when prelabeled with a norborene moiety at its N-terminus, the β-subunit of 20S proteasomes in human embryonic kidney (HEK) cells could undergo selective covalent labelling using such an approach. After lysis, labeling using tetrazine IEDDA could be observed.

**Figure 27:**
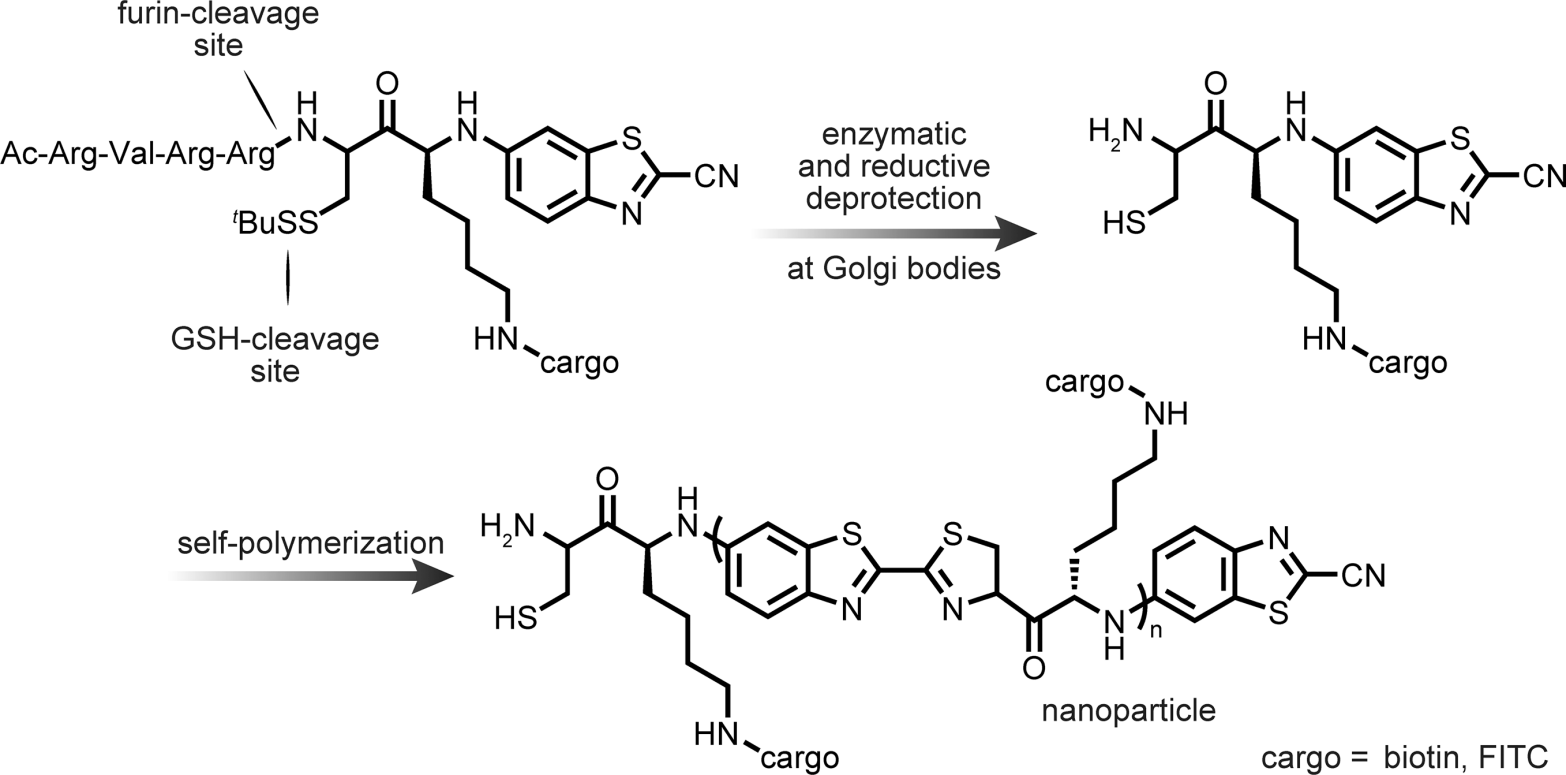
Golgi-selective polymerization. The *in situ* synthesis of “nanoparticles” is achieved through the exploitation of Golgi-localized enzyme furin.^169^

In other biocatalytic modes, a self-reactive/polymerizable molecule can be selectively activated by the presence of an organelle-specific enzyme, allowing the generation of observable “nanoparticles” in an organelle-selective manner [Figure 27].^169^ Thus, the use of a bivalent precursor containing both a cyanobenzothiazole and a 1,2-aminothiol moiety protected as a peptide with a furin-mediated cleavage site was designed to allow liberation of the aminothiol upon reaching the Golgi apparatus. Under a reducing cellular environment (mediated by glutathione (GSH)), the resulting self-reactive intermediate was observed to condense at/near the Golgi, as indicated by overlap with a Golgi-selective marker under fluorescence microscopy.

## 6 Biomolecule (Substrate) Selectivity

While all the above examples contain biomolecule selectivity to a degree, we have discussed them in terms of their “higher-order” selectivity and therefore biomolecule-specific points of principle remain. At one level, amber codon suppression (or potentially incorporation competition at other codons) provides a ready method by which metabolic incorporation modes (see sections 2, 3.5, 4.2, and 5.1) can be driven into selected proteins and so tracked longitudinally via pulsing of the reagent (the uAA or uAA precursor). For example, through pulsing a TCO-modified lysine into the protein neurofilament lightchain (NfL) in live neuronal cells, two distinct populations have been labeled with sequential tetrazine dyes via strain-promoted IEDDA (SPIEDDA, after 1 and 2 d). A first population was transported distally along the axons, but a newly synthesized second population was found to be mainly present in the cell body.^170^ Although *trans*-cyclo-oct-2-en-carbamoyl-Lys-derived IEDDA adducts can be unstable, in fact, in this system their use out-performed other variants and chemistries (a *trans*-cyclo-oct-4-enyl variant failed due to an inappropriate synthetase and an *endo*-bicyclo[6.1.0]non-4-ynyl variant failed due to observed toxicity). As an aside, these and other informative cellular studies^171^ continue to highlight how highly system-dependent the use of IEDDA, SPIEDDA, SPAAC, and other chemistries can be, suggesting that multiple factors (even beyond tetrazine reactivity, see section 7.1) play a role, including the stability of uAA precursors, their incorporation efficiencies at suppressed sites, reaction rates in chemistry once incorporated, and the resulting adduct stability.

**Figure 28:**
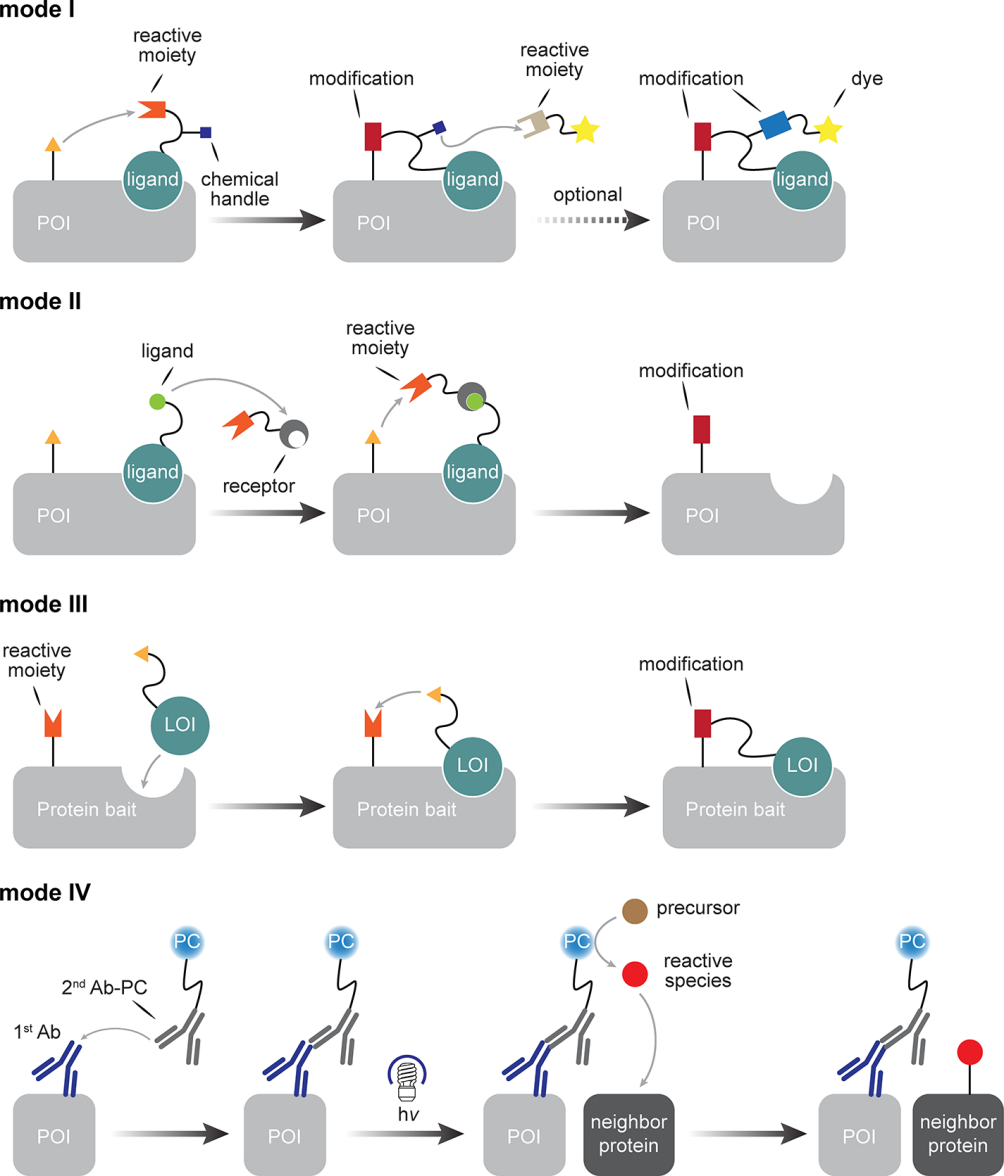
Illustration of four general modes of host–guest biomolecule selectivity. There can be considered to be, in essence, four general modes that exploit host–guest chemistry to allow selectivity toward biomolecule targets: (I) reactive guests in (biomolecule target) hosts, (II) guests that direct reactions toward (biomolecule target) hosts, (III) reactive hosts for (biomolecule target) guests, and (IV) hosts that direct reactions toward (biomolecule target) guests. Given the many variations-on-a-theme that can exist within this spectrum, we have illustrated these here with somewhat more-or-less specific cartoons. For example, in mode I, a trapped guest in a protein-of-interest (POI) host may then be further functionalized. In mode II, the “reactive splitting” of a ligand-directed reagent can be used for functionalization somewhat remote from the ligand site of a POI. In mode III, the target is now the guest, a ligand-of-interest (LOI), although potentially large and not necessarily “fully encompassed”. Examples of mode IV have often focused on somewhat relayed reactivities, as shown here, transmitted to neighboring proteins as pseudoguests, but can also be transiently targeted toward a direct ligand biomolecule. PC = photocatalyst.

The recognition of suborganelle motifs (see section 5) via specific biomolecule engagement highlights that “receptor” engagement (a term used here in its broadest sense to denote a biological host molecule and its interaction with a ligand guest) is a powerful mode of direct selectivity. We have divided biomolecular selectivity into four distinct classes [Figure 28]: (I) reactive guests in (biomolecule target) hosts, where a ligand for a biomolecule target bears a reactive group able to covalently modify the target; (II) guests that direct reactions toward (biomolecule target) hosts, where a ligand does not react with the biomolecule host itself but is able to direct a further reactive molecule selectively to the host; (III) reactive hosts for (biomolecule target) guests, where the reactive chemical functionality is installed into the biomolecule host that allows it to react with its specific ligand; and (IV) hosts that direct reactions toward (biomolecule target) guests, where the host biomolecule is not directly modified but used to direct reactive moieties toward other biomolecules that are interacting or proximal. On one level, selective ligand trapping has been known since, for example Fleet, Porter and Knowles’ use of antibodies as targeted hosts in selective labeling mediated by reactive ligand guest (mode I) arylnitrenes.^172^ Intervening variants have used reactive host proteins (mode III, SNAP-tag,^173^ CLIP-tag,^174^ and Halo-tag^175^) that are effectively single-turnover catalysts, which therefore exploit the local environment, coupled with functional-group-mediated chemoselectivity via enhanced reactivity. Of these, the human DNA repair protein *O*^6^-alkylguanine-DNA alkyltransferase (hAGT) that irreversibly transfers alkyl groups from *O*^6^-alkylguanine-DNA to its binding site cysteine residue displays useful plasticity in the substrate scope and breadth of derivatives that can be tolerated. This has led to the widespread use of *O*^6^-benzylguanine-based labels and its rapid adoption as the “SNAP-tag”; in so doing, the community has indicated a strategic appetite for selective, intracellular bond-forming methods.^173^ A recent variation on this theme suggests a method for reversible use of the system via photoresponsive *O*^6^-nitrobenzylguanine derivatives; a resulting 4,5-dimethoxy-2-nitrobenzyl-adduct could be removed upon UV light exposure to “tune” AGT activity in HEK293T and MCF-7 cells.^176^

### 6.1 Covalent Ligands for Biomolecules: Reactive Guests in Biomolecule Hosts

Covalent small-molecule inhibitors^177,178^ can be considered a straightforward example of the first mode [Figure 28, mode I]; when a normally noncovalent ligand or interacting partner for a biomolecule is modified to contain a reactive group, it can allow for covalent bond formation and thus cross-linking between the host and guest. Warheads include Michael-type acceptors, alkyl halides, and nitriles and can be incorporated into a variety of guests, *e.g.*, small molecules or peptides.

**Figure 29:**
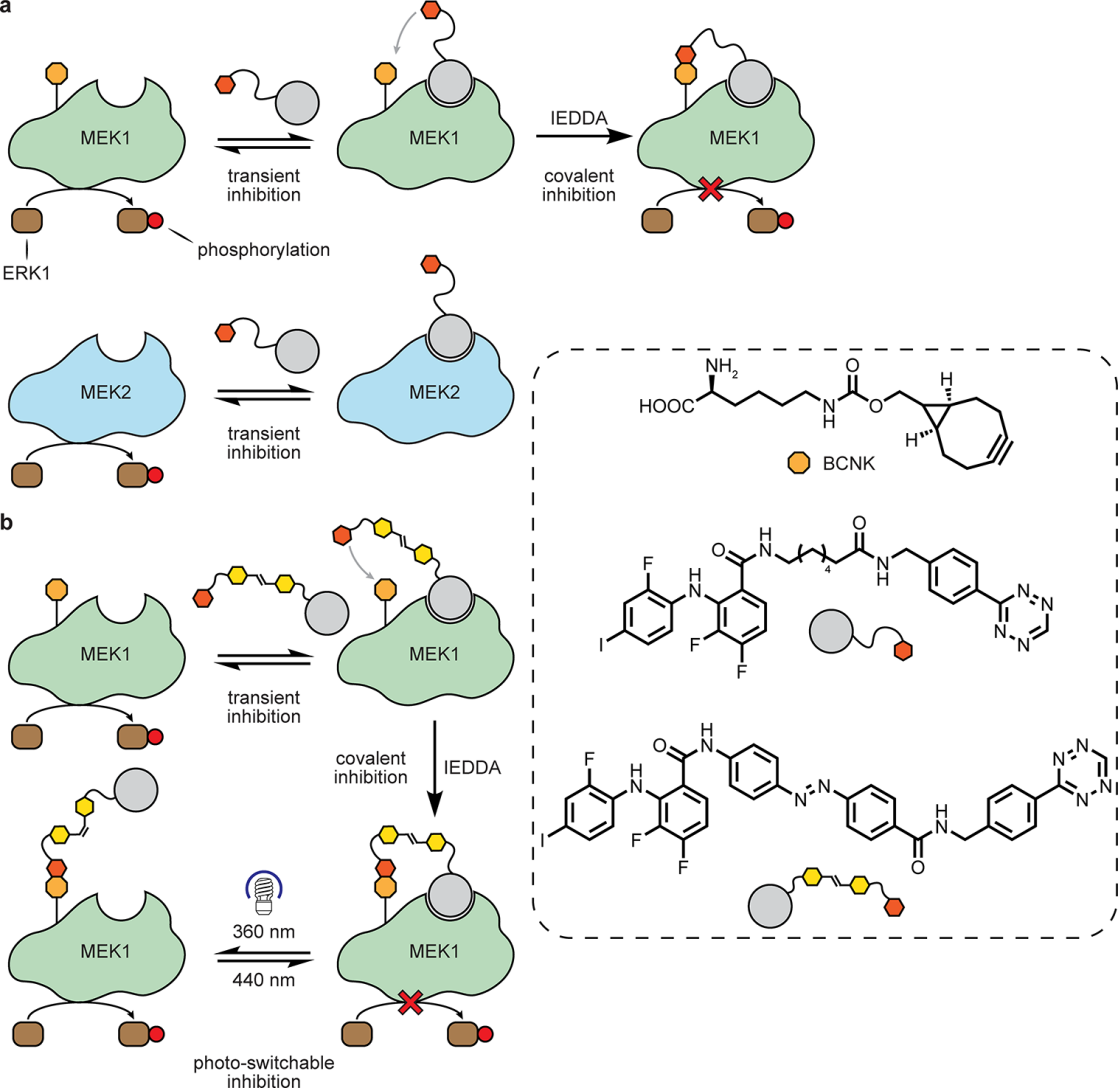
An approach for the expansion of small-molecule-mediated covalent inhibition. MEK1 kinase can be targeted over MEK2 kinase using a modified inhibitor that targets uAAs, thereby conferring selectivity when in a live mammalian cell culture^179^

This concept can be extended by targeting incorporated uAAs, thereby combining an additional aspect of biomolecule-specific chemoselectivity. This approach has allowed selective inhibition of MEK1 kinase over MEK2 kinase using a modified inhibitor, thereby conferring selectivity when in a live mammalian cell culture (unmodified inhibitor inhibits both in cells at the same IC_50_) [Figure 29]. By conjugating the inhibitor to a PEG-based tether containing a tetrazine reactive group, a modified inhibitor was generated that lost potency to wt-MEK1 (no inhibition at 10 μM). A lysine analogue bearing a strained bicyclononyne was then incorporated individually into 22 different solvent-exposed sites in MEK1. Of these variants, 10 were found to retain their catalytic ability to phosphorylate substrate protein EGFP-tagged ERK1. However, when these active variants were treated with the tetrazine inhibitor conjugate, six showed clear inhibition. When the tethered tetrazine was replaced with a phenyl moiety as a control, no inhibition was observed. An alternative analogue incorporating an azobenzene moiety in the linker could also be used, allowing its inhibition to be cycled on and off through light irradiation.^179^

Covalent ligands may also carry additional functional groups for subsequent chemoselective reactions. LasR is a bacterial quorum-sensing regulator from *Pseudomonas aeruginosa*. An isothiocyanate (ITC)-labeled variant of its cognate ligand 3-oxo-dodecanoyl homoserine lactone (3-oxo-C_12_-HSL) can selectively react with Cys79 in the ligand binding domain. This ligand contains a ketone moiety that allows selective subsequent oxime ligation with various methoxyamine small molecules (*e.g.*, containing BODIPY) in either *E. coli* or in *P. aeruginosa* as a form of pretargeted conjugation via a covalent ligand mimic.^180^

**Figure 30:**
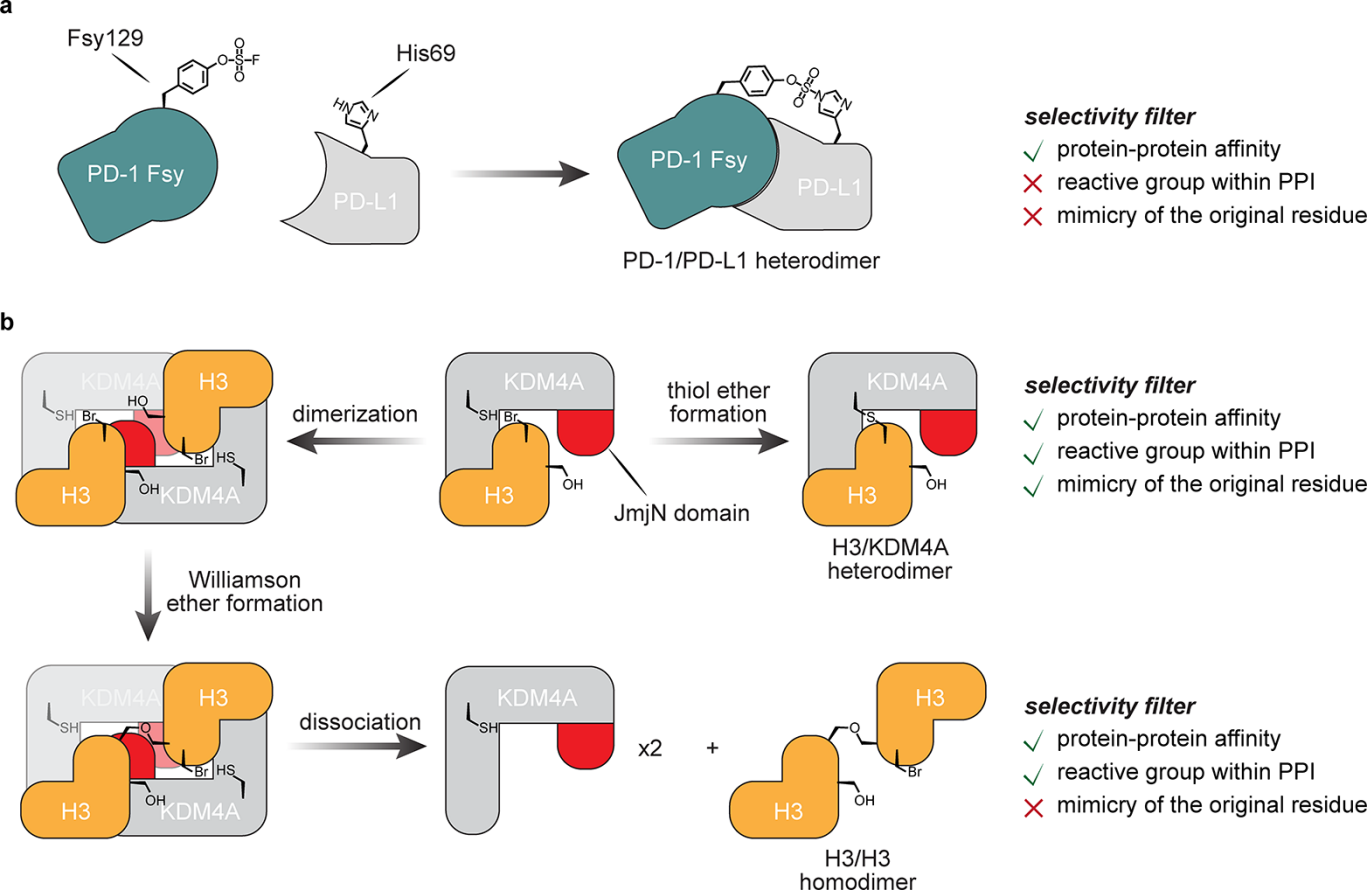
Approaches to covalent protein inhibitors via modes of biomolecule selectivity.^90,181^ Increasing levels of reactive selectivity can be engendered by exploitation of the inherently high complementarities found in protein–protein interfaces (PPIs). Through tight mimicry, selectivities driven by localization, acceleration over background (approximation), and guest–host recognition converge.

Extension of such covalent inhibition to reactive side chains in biomolecules that act as ligands creates the next step in selectivity by exploiting potential biomolecule-to-biomolecule interfaces (such as PPIs). From these, concepts of not only reactive inhibition but also functional (mechanism dependent) inhibition can now be considered in multiprotein complexes. Different examples of covalent protein inhibitors in this context have been proposed.^90,181^ In one guise,^90^ an inherently reactive moiety in a side chain, such as fluorosulfate in Fsy, is incorporated (*e.g.*, via amber codon suppression); incorporation of Fsy into the PD-1 ectodomain, for example, created a protein that was found to covalently cross-link to PD-L1 (see also above). In another example,^181^ the creation of perhaps the simplest form of reactive moiety, an alkyl halide side chain in, for example, bromo-homonorleucine (BrHnl), was utilized to exploit the differential reactivity of aqueous nucleophilic substitution in solvated (slow) and desolvated reactive complexes (enhanced, *i.e.*, inside a PPI). Incorporation of BrHnl into histone H3, for example, allowed observation not only of covalent cross-links to “eraser” protein KDM4A but also the trapping of transient H3 dimers observed through H3-to-H3 Williamson ether formation. There are essentially two modes of selectivity present in these differing examples of covalent protein inhibitors. In both, protein–protein affinity causes approximation^182^ that allows enhanced local reaction concentrations. In the latter, however, this is additionally modulated by the presence of the reactive moiety within the PPI, thereby creating a further selectivity “filter” [Figure 30]. Correct mimicry by the Lys analogue is additionally required, driving a “higher bar” for selectivity that at one level maybe considered mechanistically relevant, *i.e.*, only if the reactive analogue mimics *and* is thus localized will it react (likely via specific cross-linking modes) and so inhibit. In the former mode, PPIs will likely be trapped by more diverse and flexible cross-linking pathways. In this way, by analogy, the dual requirements of effective mimicry to form the PPI *and* reactivity constitute a protein-level example of activity- or mechanism-based inhibition [Figure 30].

### 6.2 Covalent Reagents with Directing Ligands: Guests That Direct Reactions toward Biomolecule Hosts

**Figure 31:**
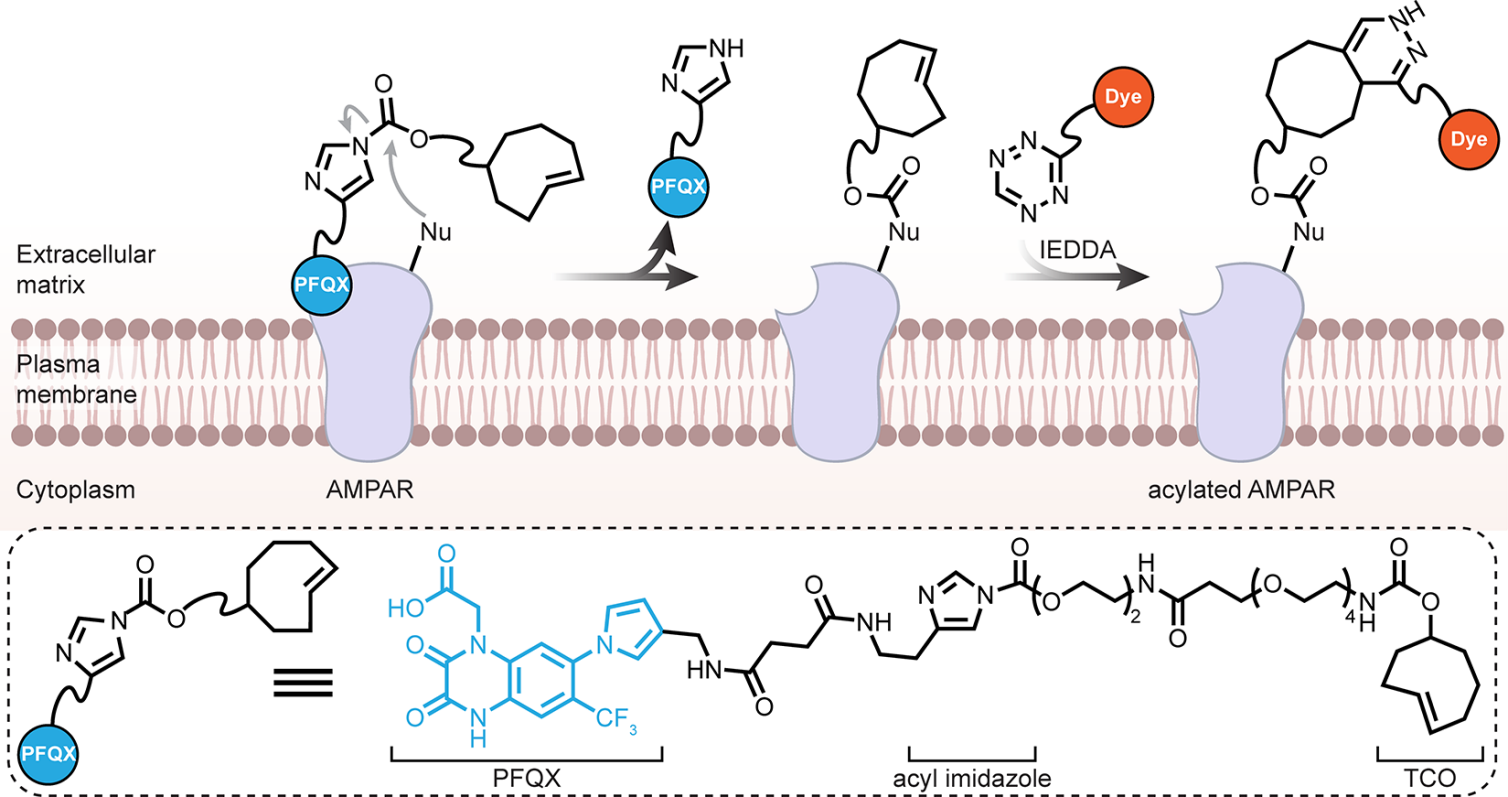
Cell-surface labeling of a specific protein (AMPAR) with a TCO through a ligand-directed acyl transfer.^184^

Hamachi has been a long-term pioneer^183^ of the second mode of selectivity (Figure 28, mode II). Here a distinction can be made from simply reactive ligands (see section 6.1) in that the ligand moiety is not itself trapped, bringing with it potential advantages of “freeing up” this binding site (*e.g.*, for reuse). This can be applied in differing ways. One of these is essentially stoichiometric, where a covalent guest reagent is guided to a biomolecule via a moiety that is both a binding ligand and a leaving group in, for example, acyl transfer or direct nucleophilic substitution chemistry. In this way, little trace of the directing group is left but chemistry is guided toward a target of interest. The neurotransmitter α-amino-3-hydroxy-5-methyl-4-isoxazolepropionic acid (AMPA)-type glutamate receptor proteins (AMPARs) are present in the excitatory neurons of brain, which play vital roles in memory formation. A TCO that has been PEG-linked to acyl imidazole that itself bears an AMPAR ligand 6-pyrrolyl-7-trifluoromethyl-quinoxaline-2,3-dione (PFQX) has been shown to direct TCO-plus-linker labeling via acyl transfer to AMPARs. These can then be labeled with tetrazines via IEDDA in a manner that powerfully allows use in monitoring distribution and trafficking in living neurons [Figure 31].^184^

**Figure 32:**
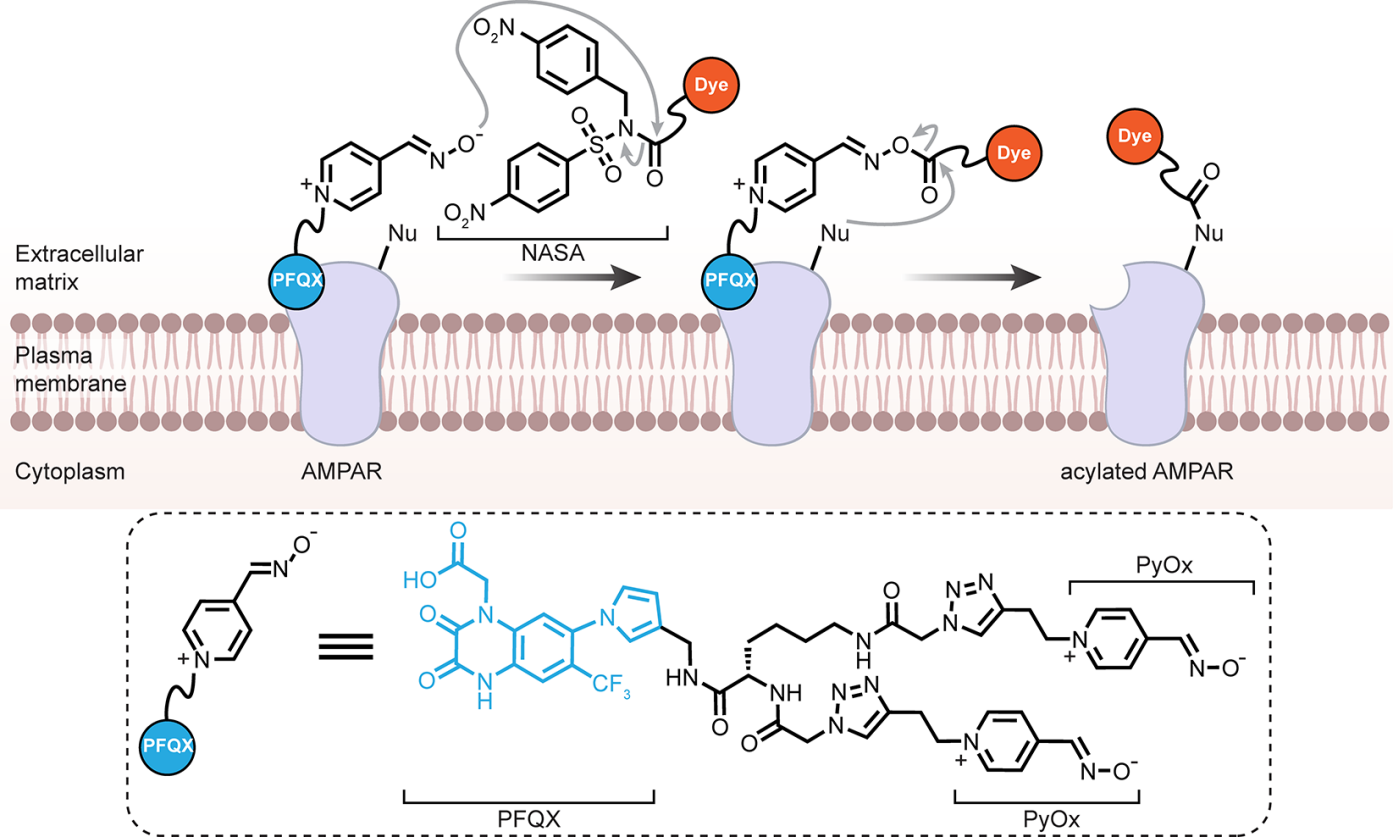
Cell-surface labeling of a specific protein (AMPAR) with a dye through ligand-directed catalytic activation of the mild electrophilic NASA substrate.^185^

In a second mode, a ligand is directed as a catalyst toward a target; this then drives the local reaction. Prior combinations of DMAP-derived organocatalysts and thioester-type acyl donors have, for example, been utilized for selective labeling of target proteins.^183^ However, these strategies can suffer from the low nucleophilicity of DMAP, off-target modifications, and the low stability of thioester acyl donors, limiting their applications in complex biological contexts. A recently developed generation of selective acyl-transfer labeling reagents has been successfully used to target proteins of interest on the surface of living cells. Using ligand-tethered pyridinium oxime (PyOx) species with suggested high nucleophilicities, mild electrophilic *N*-acyl-*N*-alkylsulfonamide (NASA) acyl donors allowed acyl transfer reactions to neighboring amino acid residues within target ligated proteins. With this strategy in hand, endogenous cell-membrane protein carbonic anhydrase XII was successfully labeled in living cells using a combination of a phenylsulfonamide-PyOx conjugate and NASA reagents. By transiently expressing the GluA2 subtype of AMPAR in living HEK293T cells, the use of a PFQX-PyOx conjugate allowed the selective labeling of Lys470 and Lys677 within GluA2. Moreover, this technique was also successfully used in selective labeling of endogenous AMPARs in acutely prepared living hippocampal and cerebellar slices from mouse brains [Figure 32].^185^

**Figure 33:**
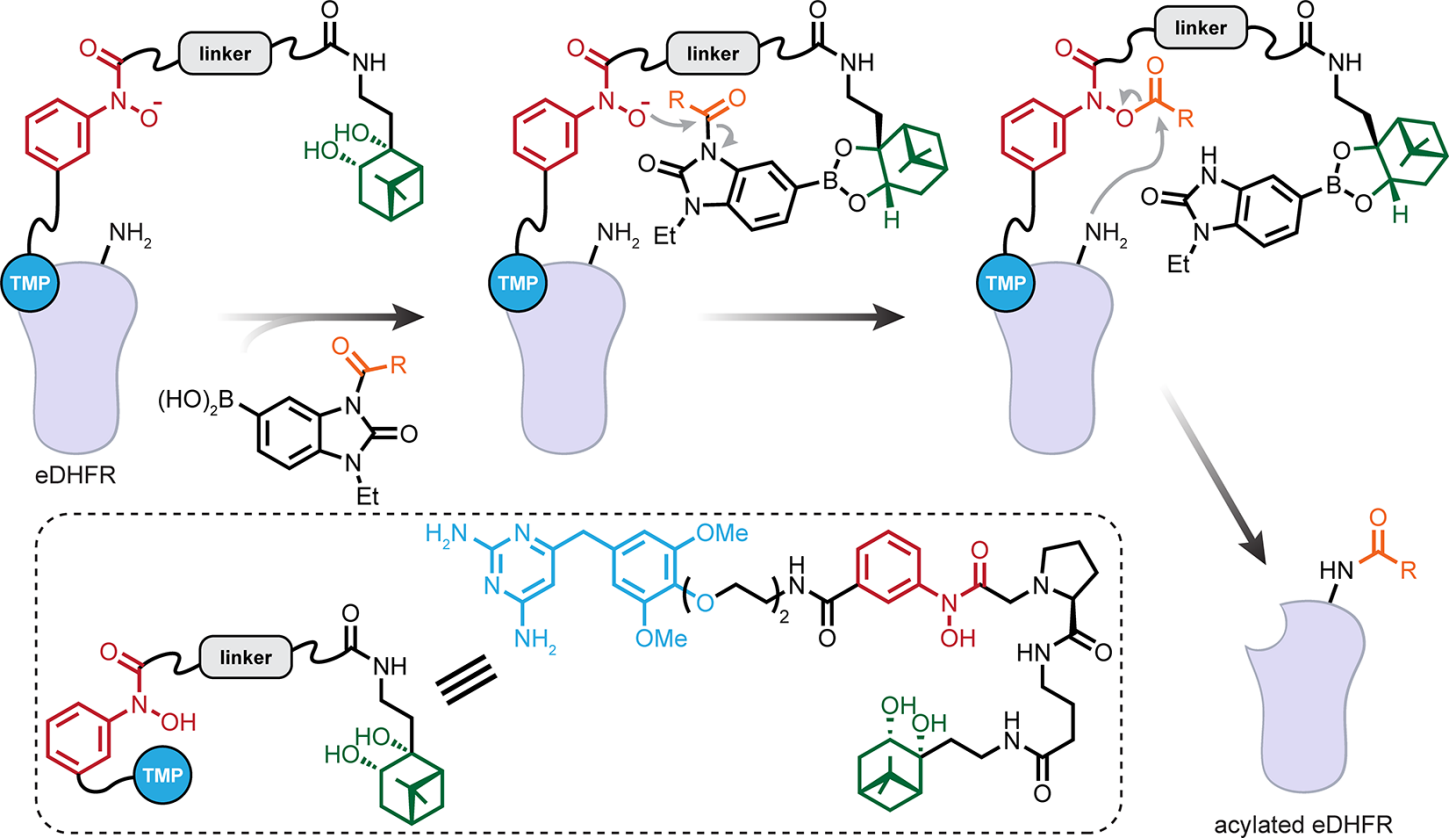
Residue-selective labeling through boronate formation and subsequent acyl activation.^186^

In these systems, nucleophilic catalysts direct the local reactivity. Enhanced recruitment of reagents has recently been suggested via boronate formation to augment this type of manifold. A selective ligand (trimethoprim, TMP) conjugated to a hydroxamide containing a linker and a nopoldiol-derived diol moiety plays the key role of recruiting a boronic acid (via boronic ester formation) that possesses an activated acyl moiety. In HeLa cells overexpressing *E. coli* dihydrofolate reductase (eDHFR), this directed reactive electrophilic acyl units to eDHFR, leading to selective labeling of a single Lys32 on the protein surface [Figure 33].^186^

Specific mimics of native small-molecule electrophiles, particularly electrophilic lipids, have been generated and targeted to specific proteins via covalent ligand-directed release. This allows potential study of the effect of a given electrophilic lipid on cell signaling roles in a controlled manner. Through the expression of a given POI bearing a Halo-tag protein module, cellular incubation with a small-molecule probe containing an appropriately reactive linker and a component able to release a reactive species (in this case, 4-hydroxynonenal (HNE)) upon light irradiation (via an alkoxyanthraquinone) then enables relayed delivery. Treatment of cells expressing a Halo–target fusion (Keap1) with the probe and subsequent irradiation resulted in targeted delivery of the reactive electrophile to the Keap1 protein, with the selectivity essentially driven by the fused Halo-tag module. In the absence of targeting, only global nonspecific labeling of the proteome was observed.^187^

Such ligand-directed light-released reagents can be complemented by light-reversed systems. The cAMP-dependent protein kinase catalytic subunit (PKA) has been targeted using a known small peptide ligand, PKI, conjugated through a nitrobenzyl moiety to a ruthenium complex (RuTAP_2_Phen)^2+^. Impressively, when the construct was microinjected into cells (to a final concentration of 2–5 μM), it was able to “pseudoirreversibly inhibit” endogenous PKA (ePKA) by cross-linking to active site residue Trp196 in the presence of blue light. After irradiation with UV light, cleavage of the nitrobenzyl moiety liberated the PKI portion of the construct, thereby restoring activity to ePKA (leaving Trp196 only partially modified).^188^

### 6.3 Reactive Biomolecule Hosts

A biomolecule host (such as a protein receptor), if it is rendered reactive through the installation of a reactive chemical functional group, may become capable of covalent bond formation with a binding partner.

Tyrosyl-fluorosulfates can usefully generate protein RNA cross-links, for example, when introduced into RNA-binding proteins via amber-codon suppression.^189^ When placed into an expressed known binder of *N*^6^-methyladenosine bases, namely, YTHDF1, this residue cross-linked at *N*^6^-methyladenosine bases in *E. coli* and HEK293T, allowing identification of new modification sites.^189^ When placed into carbohydrate-binding proteins, namely, lectins, a parallel carbohydrate-directed selectivity can be imagined. A reactive variant of the sialic acid-binding protein Siglec7, which in unmodified form regulates natural killer cells, was inferred (from greater killing compared to wild-type) to have redirected killing activity, possibly through directed reaction with cell-surface carbohydrates on a number of cancer cell lines overexpressing sialic acids.^190^

### 6.4 Covalent Reagents with Directing Hosts: Hosts That Direct Reactions toward Biomolecule Guests

There has long been exploration of what might be loosely termed “proximity” methods, some of which seek to exploit the creation of a diffuse “wave” of reactive small-molecule reagents emanating from a biomolecule, thereby generating interesting “local” selectivity. These are often generated by a biocatalyst or biomolecule–catalyst hybrid that attains its localization through protein–protein interface formation and so can report on endogenous (interactomes) or contrived (Ab-targeted) localization events. This concept relies on a finely tuned degree of selectivity within the given system. In attempts to map proteins within a defined proximity of a protein of interest (POI), a high degree of selectivity for the target POI is required. In one variant, the biocatalysts may simply be targeted through fusion to a probe biomolecule, as is used in bioID^115,191^/turboID^116^ or APEX^192,193^ (see section 5) approaches. In another, an antibody-mediated approach is combined with the generation of reactive species that are capable of labeling proximal proteins but are quenched within a rapid time frame so as to prevent extensive diffusion that might limit “local” selectivity. This narrow window has been nicely exploited in the following examples, allowing for some selectivity in the spatial domain.

**Figure 34:**
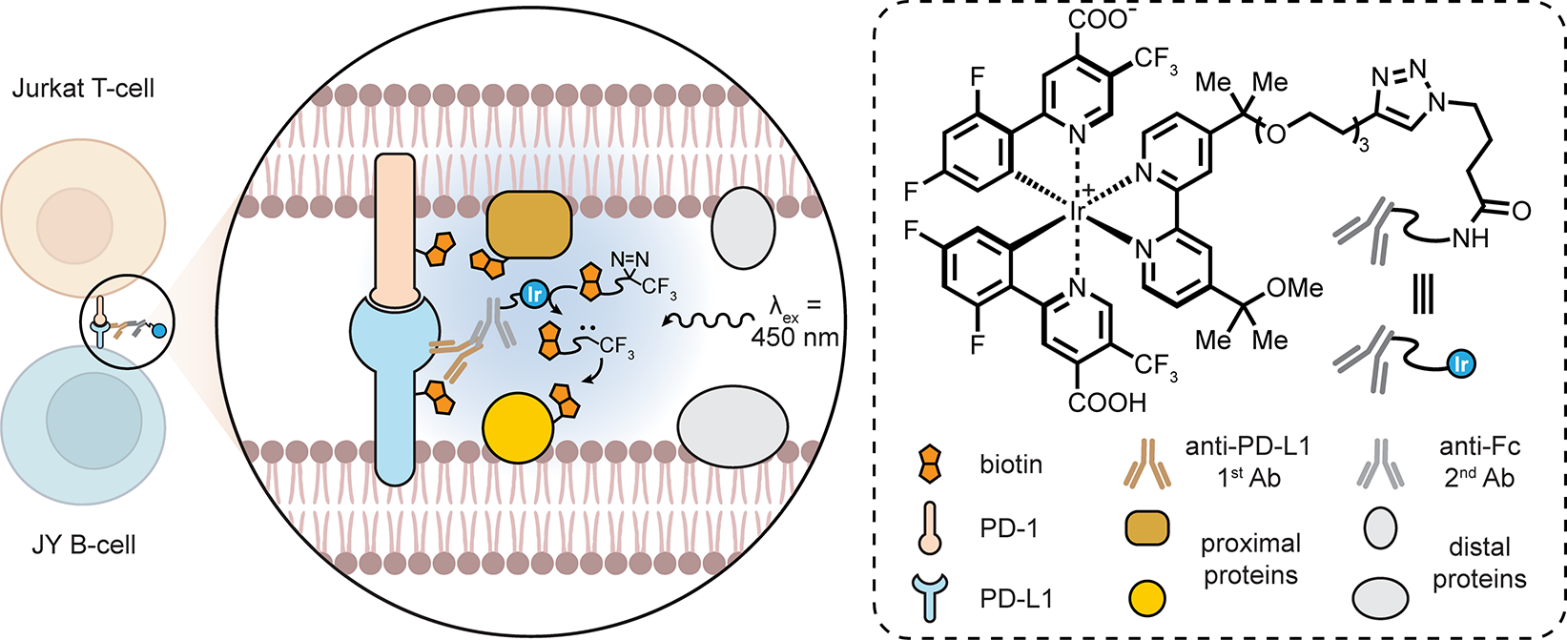
Antibody-tethered Ir photocomplex converts CF_3_-diazirine biotin probes to reactive carbene species under blue light irradiation. When the complex is tethered to an antimouse mAb, this approach can be used to target a mouse-derived primary antibody onto a cell surface to drive proximal reactions.^194^ Fc = fragment crystallizable.

An Ir(III) photocatalyst ({Ir[dF(CF_3_)ppy]_2_(dtbbpy)}PF_6_ bearing appended carboxylate, PEG and an alkyne moiety) has been conjugated with a secondary antimouse antibody that had been prefunctionalized with azides via NHS chemistry, resulting in a mixed conjugate pool (mAb/complex = ∼1:6) [Figure 34]. This pool can be used to recognize mouse-derived primary antibodies that target proteins-of-interest in cell surfaces. Upon concurrent treatment with a biotin-tethered CF_3_-diazirine under blue light irradiation (450 nm), carbene generation occurs; these carbenes react in a spatially limited manner (<4 nm suggested) with biomolecule neighbors. The carbene generation process is proposed to occur through a Dexter energy transfer process from the excited Ir-photocatalyst T_1_ state. By targeting PD-L1 in living lymphocytes, labeling within an immunosynaptic junction was tested.^194^

**Figure 35:**
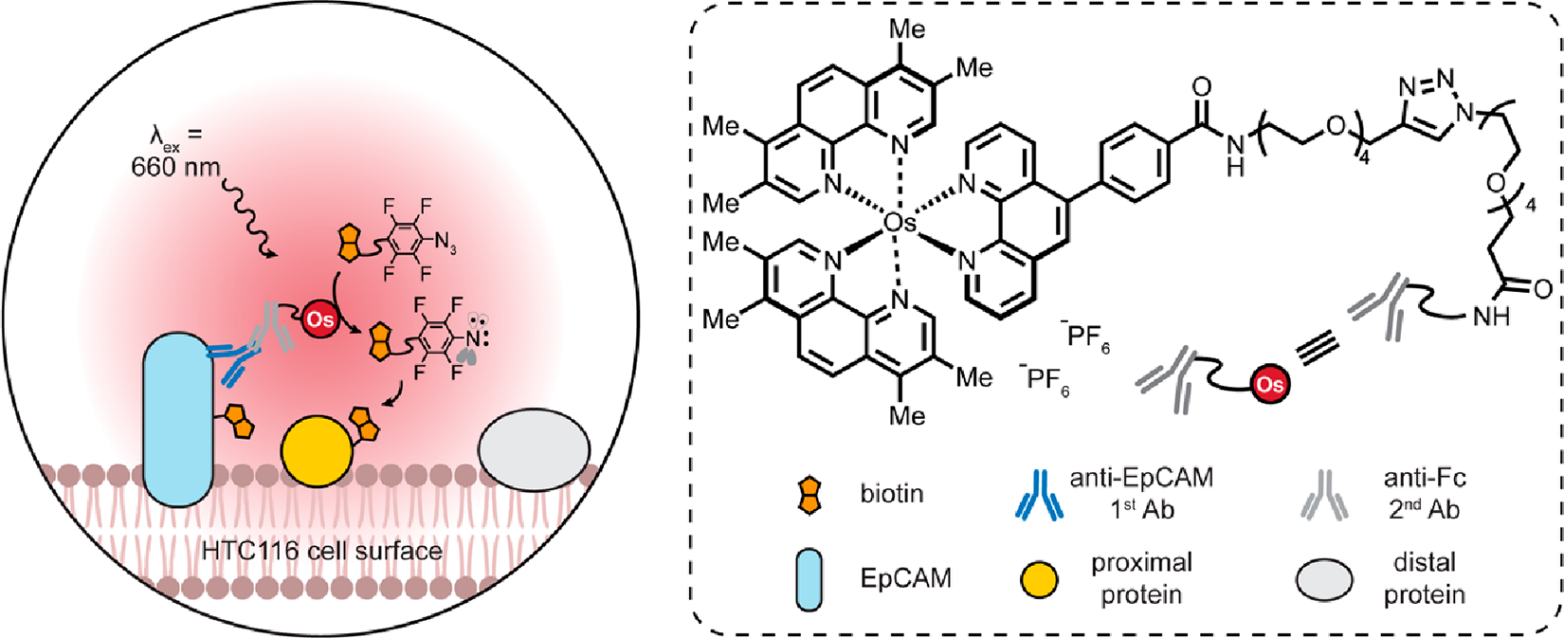
An Os photocatalyst variant conjugated to a secondary antimouse mAb can activate perfluoroarylazides to generate nitrenes. Under deep-red light irradiation, proximity-based cell-surface labeling can be achieved.^195^

In a similar vein, a pool of antibody-conjugated osmium photocatalyst [Os(3,4,7,8-Me_4_phen)_3_](PF_6_)_2_ has been developed for local activation [via *E*(Os^III^/Os^II*^) = −1.05 V vs SCE] of perfluorinated aryl azides under deep red light (>600 nm), selectively generating triplet nitrene species close to the catalyst [Figure 35]. These osmium complexes were conjugated via a PEG-alkyne handle, which in a similar manner to above, was reacted via CuAAC with an azide-prefunctionalized secondary antimouse mAb to give a mAb-Pcat conjugate pool in an ∼1:6 ratio. This system displayed higher selectivity than classical aryl azide photoaffinity labeling under high-energy UV or blue light, where resulting singlet nitrene species (*t*_1/2_ = 1–10 ns) rearrange to longer-lived ketenimine species (*t*_1/2_ = 5 ms to 1 s), giving the possibility of nonselective and nonlocalized labeling.^195^ Other red-light variants exploit the formation of aminyl radicals.^196^

Such directed catalysts may also be diversely hybrid in nature. In one example, nucleic acid-based catalysts have been applied in a manner that is, in part, strategically similar to some uses of APEX. Thus, aptamer-G-quadruplex/hemin complexes localized on cell surfaces are proposed to generate phenoxyl radicals that label adjacent proteins.^197^

**Figure 36:**
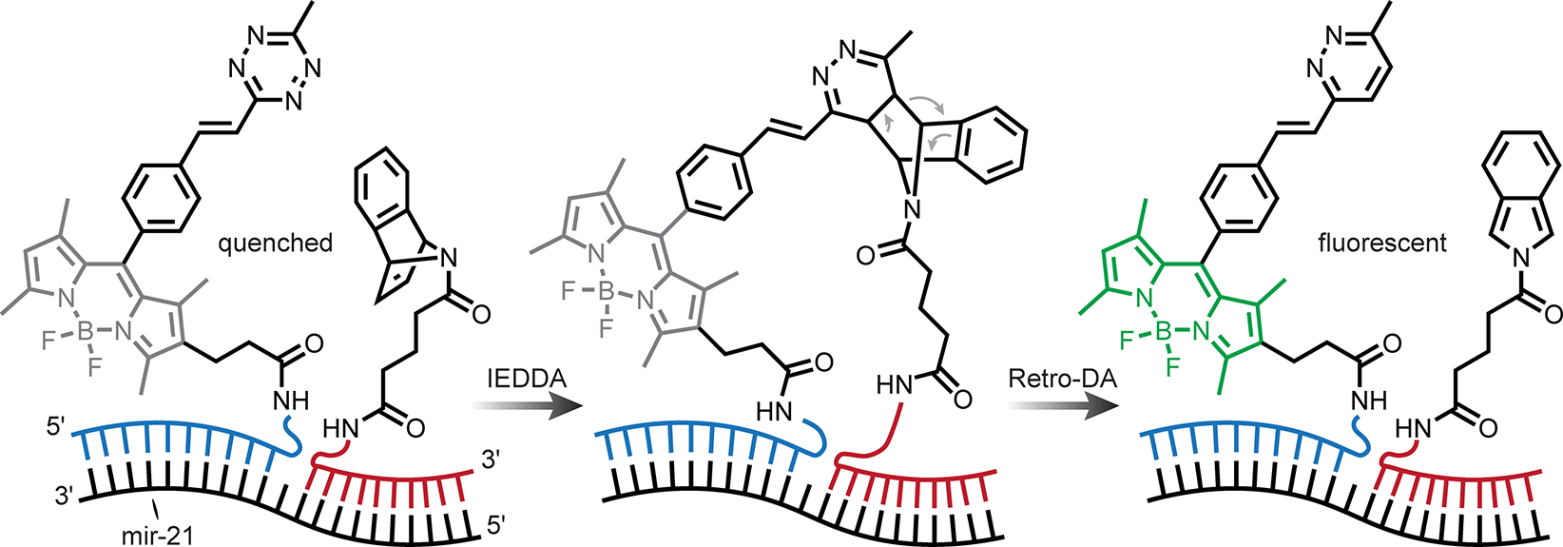
Proximity-mediated reaction in a split RNA. The two halves of a split RNA strand bearing reactive moieties at the 3′ and 5′ ends can undergo proximity-mediated reaction. This can allow for turn-on fluorescence imaging of specific miRNA driven by nucleic acid complementarity.^199^

Proximity can also be induced in mutual binding, a strategy long-exploited in nucleic acid science where, of course, complementary binding sequences can be faithfully designed and encoded in a near-unique manner. In addition to driving many familiar endogenous processes, it is worth revisiting the modes of biomolecule-selective reaction that these may mediate.^198^ By appending, for example, reactive components to the 5′ and 3′ ends of a split strand of RNA, selective reactions can be driven by proximity when both halves of the RNA bind to a complementary native strand, even *in vivo*. In one example, a BODIPY fluorophore internally quenched by a pendant tetrazine can be “turned on” by IEDDA tetrazine ligation followed by a retro-DA reaction with a 7-azabenzonorbornadiene component to generate a fluorescent pyridazine. This has allowed selective imaging of endogenous miRNA in a selective manner—mediated by complementarity—and was shown to be disrupted by the change of a single base pair in the target miRNA [Figure 36].^199^

### 6.5 Affinity Proteolysis: A Prominent Case of Biomolecule-Directed Biocatalysis

In these ligand-directed chemistries, both directed reactivity and directed chemocatalysis have been exploited. In keeping with our comparison of LSF with post-translational modification, the cleavage of protein backbones through enzyme-mediated proteolysis can be considered to be a form of functional activation (in the case of zymogens) but is typically a process for degradation. In some ways, the recent resurgence of affinity proteolysis methods, noting some long-standing concepts,^91,200,201^ highlights some of the strategic questions around all forms of LSF when applied in living systems. The notion of proteolysis-targeting chimeras (PROTACs) and other degradation methodologies based on bivalent interaction has become a widespread and broadly accepted methodology.^202^ There is strategic elegance in the use of ligand-directed reactivity (see section 6.2) to engage an endogenous activity, such as an E3-ligase. However, the underlying endogenous pathways^203^ and associated kinetics that permit such a three-body/ternary complex-mediated mechanism to be successful still remain somewhat opaque. In comparative ternary complex methodologies, an intermediate state (*e.g.*, binary) of high specificity is typically required to drive a pathway with high-level activity. In many cases, the lack of clinical translation of some targeted systems^204^ may be a consequence of missing underlying analyses associated with details of kinetic (*e.g.*, *k*_on_ and *k*_off_) parameters. Such analyses would similarly underpin the broader fields of substrate-, ligand-, and proximity-directed methods. Given the knowledge associated with inhibitory complexes from synthetic affinity proteolysis systems^200^ and the need for systematic variation for success^201^ to overcome such inhibition, it is perhaps informative that so many newly suggested variants apparently seem to form productive systems. Undoubtedly, greater innovation in determining “*in vivo*” kinetic parameters associated with such processes will inform the broader field of late stage functionalization of living systems.

## 7 Chemoselectivity

The fundamental basis of bioorthogonal chemistry has been one of chemoselectivity to a very large extent, and many excellent prior reviews have now explored this large depth and breadth of reactivity.^8,11,205−207^ In this regard, two focuses have emerged that may have partially distracting aspects and that have relevance to this Review, for which we would like to offer some caution: apparent kinetics and side-reactions.

For the first, the logic of “strive for speed” stems from a sensible desire to “match” fast kinetics with the apparent dynamics of biological systems, that is, to freeze or “catch nature in the act” as it were (before protein turnover, loss of location, *etc.*). This is clearly desirable in some ways but has to date largely been examined through the crude lens of pseudo-first-order kinetics (with excess of one partner) in small-molecule models.^206,207^ While this provides an excellent baseline and allows tables of comparisons,^206,207^ by their very nature these do not correctly consider the local concentrations of substrates *in vivo*, and few researchers have tested the differing relative reactivities that may be present in intact biomolecules through on-biomolecule methods.^208−210^

For the second, the elucidation of side reactions that may erode a notion of “true bioorthogonality” are in some sense important, but overfocus may not prove productive. Are we to be surprised if no functional groups are truly biooorthognal? As the archetypal examples, azides and alkynes are functional groups with immediately obvious oxidative/chemical potential (“bond count”). Moreover, the observation that (strained) alkyne variants may react with thiols^42,211,212^ (such as in Cys) has only contextual relevance, *i.e.*, in answering the question “Are local concentrations *in vivo* sufficient to sequester reagent (reduce reactivity) or drive unwanted reactions (false labeling)?” Observations of serum component interactions both *in vitro*(42) and *in vivo*(41,43) suggest that blood is a less productive reaction medium for strained alkynes. The high intracellular concentrations of glutathione may also suggest that side-reactions may be more prevalent inside cells. However, the clearly successful and selective nature of cell-surface SPAAC reactions highlights where utility is more readily achieved. As we have sought to highlight throughout this Review, the key to biological function will be for sufficient *selectivity* to be achieved; this will be given by the compounded kinetics that define such global selectivity (including those for a given ligation and side-reactions, but not solely). A focus on the *consequences* (if any) of speed or side-reactivity is therefore paramount.

There is therefore no need to recapitulate the cataloguing of multiple reactions nor indeed their potentially useful categorizations under bond-forming processes^205^ here. However, specific features of relevance to higher-order selectivities seem pertinent.

### 7.1 Inverse Electron Demand Diels–Alder Reactions as the “Cycloaddition” of Choice

In seeking the “ideal” click or bioorthogonal reaction, a pseudoevolution of reaction utility has emerged, largely tested by the fitness as measured by these two features. From these, IEDDA reactions have emerged as prime candidates, focused on the near-exclusive use now of 1,2,4,5-tetrazines as electron-poor “dienes” with a variety of strained, electron-rich dienophiles.^213−215^ Despite this intense interest, elegant studies highlight that even the fundamentals of tetrazine reactivity still remain only initially understood,^216^ noting an intriguing role for distortion in the tetrazine, not just its partner, that may be modulated by intramolecular repulsion. Starting from the popular methyl-2-pyridyl-tetrazine, this allowed the separation of reduced stability from reduced reactivity when tested in some biofluids. Such systematic studies are rare but valuable; we have already noted (see section 6) similarly valuable cellular studies of dienophile use.^171^

### 7.2 Metallocatalysis

A focus on the rate of mutual second-order reactivity is also born out of a desire for selectivity; enhanced cycloaddition rates (for example) may outcompete off-target modifications and reagent decomposition. Such methods may therefore be complemented by other modes of selectivity arising through enhanced reactivity, prime among which is the use of biomolecule-directed catalysis.^217^*In vitro*, the basic analogies between nature’s typical modes of catalysis and chemocatalysis are at one level obvious, yet the *de novo* application of metallocatalysis *in vivo* remain relatively rare. Some illustrative examples highlight opportunities.

Since suggestions of benign utility,^218−220^ the application of the wealth of abiotic metallocatalyzed processes to engender new selectivities has appeared immediately attractive.^221,222^ Transition-metal biocatalysis (essentially first-row) is, of course, central to biology and at the heart of many widespread metabolic processes.^223^ However, the simple binary notion that abiotic (“heavy”) metals are either toxic or not has perhaps overly dominated thinking. As for all chemistry, unwanted side-reactions may emerge at the wrong concentrations. However, given the varied use of metals as cofactors, a more nuanced analysis raises the possibility of *in vivo* modes of selectivity, some of which may indeed even exploit the natural modes of transition metal coordination (*e.g.*, for directed catalysis toward metal-binding sites in targets^224^). In this context, the ability of metals to exploit not only alternative coordination geometries in biomolecule targets but also reaction manifolds and corresponding redox potentials that nature will not have accessed creates alternative reaction pathways and therefore useful orthogonal selectivities.

The Suzuki–Miyaura reaction, mediated by preformed Pd-pyrimidine complexes, can be used to target iodo-Phe, introduced through stop-codon suppression, in *E. coli*.^219,220^ Homopropargylglycine (Hpg), introduced to the C-terminus of ubiquitin in Met-auxotrophic *E. coli* cells, can be addressed with similar complexes, allowing Pd-mediated Sonogashira reactions.^225^ Lysyl propargyl carbamates or iodo-benzyl carbamates introduced through stop-codon suppression in *E. coli*, *Shigella*, and *Salmonella* bacteria are also successfully targeted by Pd complexes.^226^

The nature of encapsulation or intracellular uptake and delivery of an abiotic metal in these systems is an interesting aspect that is not fully understood and could be a powerful selectivity element. The ligand state in a metal source likely has a clear bearing^218,226,227^ on both reactivity and possible transport, if only to allow metal centers to find preferred catalytic states, perhaps on biomolecule targets.^224^ In some modes, protein carriers have been suggested.^228^ And while the uptake of transient soluble catalysts may be a preferred mode of application in its mimicry of biology, the prospect of heterogeneous metallocatalysis might also present powerful opportunities even for (*e.g.*, particle-based) delivery vehicles, as has been shown for Pd nanoparticles encapsulated in polystyrene^229^ or biocompatible poly(lactide-*co*-glycolide)-polyethylene glycol block^230^ polymers; the latter were reported to accumulate in tumor tissues when administrated intravenously.

Indeed, even highly hierarchical heterogeneous metallocatalysis systems have been suggested. Mitochondrially-directed activity mediated by macroporous silica nanoparticles has been described,^231^ where these are embedded with ultrafine Pd nanoparticles within their “inner surfaces” and further functionalized with azobenzene and β-cyclodextrin moieties. When internalized into HeLa cells and exposed to UV light, selective activity (as measured by fluorescence in the mitochondria) was seen from use of an Alloc-caged fluorophore reporter, as well as from combined triphenylphosphonium aryl boronate ester and nonfluorogenic fluorescence-triflate precursors (reacting via a suggested *in situ* Suzuki–Miyaura reaction). Here “unblocking” of pores mediated by *cis*–*trans* azobenzene isomerization within the nanoparticle is suggested as the light-controlled “switch”.

Purified exosomes have also been suggested as delivery vehicles. Those derived from non-small cell lung carcinoma A549 cells (named Exo^A549^) were incubated with K_2_PdCl_4_ and treated with CO to mediate the formation of Pd^0^. These treated exosomes were observed to generate self-assembled Pd “nanosheets”.^232^ Apparent cell-type selectivity is suggested to be driven by an unspecified affinity of the exosome for its parent cell type.

## 8 Conclusions and Future Perspectives

In this Review, we have sought to highlight the powerful role of chemical selectivity, as a notion derived from small-molecule systems, when translated into more complex living systems. Some consideration for the corresponding methodology that will be required in the future is therefore due and can perhaps be summed up by two questions: “How does one design a reaction that will selectively ‘edit’ biology?” and “What outcomes should we expect?”

In seeking to answer the first question, some may find the analogy that we have drawn here between selectivity in small molecules and the use of notions of selectivity to address complex biology somewhat contrived. However, we argue that clear separation of modes, as in small-molecule chemistry, remains strategically important in planning the ultimate goal of reaching one molecule type in a given living organism in a reproducible manner through directed chemistry. For example, the obvious differences associated with partitioning into organelle-associated structures allow chemistry (and the molecules that may mediate chemistry) to be arrayed in a spatially addressable manner that is deeply powerful.^166^ The resulting ability to near-stably array substrates and reagents is a “slower”/near-thermodynamically controlled mode at a selectivity level that can be considered to be quite distinct from those at selectivity levels that will need to exploit greater dynamics, such as clearance from serum into targeted organs. When these modes of selectivity are then further coupled with more chemically traditional methods, such as approaches to regio-^208,210^ and stereoselective^233^ biomolecule manipulation, a possible path to fully precise organismal editing emerges.

The viable reaction types are undoubtedly still limited. Previous reviews^14^ have correctly highlighted that the reactions that are currently applied to living systems are a very narrow subset: variants on essentially the same cycloaddition themes, largely restricted to SPAAC and variants of IEDDA, *e.g.*, Tz + TCO reactions. Along with early applications of Staudinger ligation, these have dominated *in vivo* applications. As we have noted, reaction choice appears still to be driven on the whole by a perception of needed fast kinetics, despite clear and excellent *in vivo* studies highlighting that enhanced efficacy is in fact derived from reaction *type*. The greater efficiency^41^ in splenocytes of the much slower SL is likely attributable at least in part to the notions of global, layered selectivity that we have tried to set down here, including off-target sequestration, *e.g.*, by serum for certain modes of cycloaddition.

In the search for other reaction paths, methodological themes emerge. A consideration of what Jencks termed approximation^182^ remains useful and at one level is a driver of greater selectivity in intracellular systems than might instinctively be anticipated from *in vitro* comparison. As well as the inherent compartmentalization of cellular structures, the greater density of cellular fluids^234^ (and so presumably reduced diffusion) may give rise to stronger proximity effects intracellularly than in model systems. The now widespread utilization of so-called proximity-directed methods highlights an assumption that this is a broadly effective mode. This may only require, therefore, the initial localization of catalyst or mediator such that the combination of these methods with amplifying (*e.g.*, catalytic) effects can drive effective modes for “local” selectivity. In this sense one can observe many parallels: the pioneering ligand-directed chemocatalysis of acylation chemistry of Hamachi^183^ is at a functional level analogous to the directed biocatalysis of acylation mediated by BirA variants in bioID/turboID.^115,116,191^

Model systems can be useful if they are focused on parameters of perhaps greater relevance to *in vivo* chemistry. One interesting idea is the development of “biological solvents” that might better mimic the conditions that will be encountered in living systems. While the use of serum or plasma could be readily considered (and sometimes employed), ambiguity in composition will clearly hamper application. Perhaps the time is right for a given source of serum or serum-like solvent to be adopted by a community interested in probing *in vivo* chemical function (*e.g.*, Dulbecco’s phosphate buffered serum^235^). As noted above, this is perhaps even more the case for intracellular components where the estimated fluid densities and concentrations in the cytosol might instead suggest gel-like properties, with the implied effects (“crowding”) that this will have on diffusion-limited processes. The proper mimicry of such fluids (as well as their phase transitions) could prove a profound challenge with great value to the field, illustrated in part by emerging observations associated with the proposed function of biological condensates.^103^

Metabolic incorporation has played a prevalent role in priming molecular substrates in living systems with functional groups that may be addressed primarily through chemoselectivity; it is a powerful method for installing chemically reactive handles/tags into living wild/wild-type systems. In this way, through a “pretreatment”, one can exploit new, induced, and noninherent selectivities. The degree of selectivity that can be imparted to a specific tissue or cell type could therefore, in principle, be varied by exploiting (or even changing) the biosynthetic pathway that is being hijacked or simply by exploiting (or even changing) different expression levels of corresponding enzymes involved in the biosynthetic pathways. Such pathway engineering strategies to drive unnatural residue (uR) incorporation (*e.g.*, uAA or uG or uL) are only in their infancy, and the ability to enhance selectivity in metabolic incorporation strategies is worthy of further research. The identification of further pathways that lend themselves to the modular notion of other residues that may be incorporated *en bloc* is also worthy of greater pursuit; use of atypical modules (*e.g.*, lignin^73^) are rare. Moreover, in an organismoselective context, exploitation of the intrinsic biosynthetic machineries of different organisms and their ability to handle uRs is likely a fruitful avenue.^236^

Increasing levels of molecular characterization that the synthetic chemistry methodologist would consider essential are undoubtedly driving great change, and the confluence of new technologies is opening a door to a bold future. The sensitivity of MS^n^ methods continues to improve, albeit perhaps overly focused on more tractable peptidic structures, and this now makes cell-level characterization for more abundant biomolecules initially feasible. This in turn allows new bars to be set; the routine demonstration of molecular level fidelity of bond-forming “editing” of biomolecules within living systems should in time be expected as a guide to the notions of selectivity that we have set down here and the resulting gains of function caused. This will be the basis of new exciting structure–activity relationships.

In turn, therefore, this leads to some answers to the second question that we posed, that of goals and expectations: we suggest that where LSF of living systems has been used, it has essentially largely been applied to localizing either a dye or a dose (with rarer examples of retrieval). The opportunities therefore to “edit” biology to create new function—akin to nature’s use of post-translational modification—have been somewhat neglected, and this creates an enormous future opportunity. This will allow us to move beyond simply the role of chemistry in “localization biology” or “retrieval biology” through *in vivo* bond formation to “gain of function biology”.

For example, many applications to date have been essentially restricted to imaging and drug delivery strategies. In some cases, these have valuably expanded strategies previously based on other localization methods (as in pretargeting, where the move from biotin–avidin affinity to covalent attachment brings benefits of selectivity and compatibility), while others are essentially chemically mediated prodrug release, such as so-called “click-to-release”.

As we note in the introduction, we do not seek to dismiss the possible lessons that can be learned from such prodrug-type chemistry in terms of compatibility and utility of chemistry in living systems, but the scientific aims are different from the focus of this Review. In some systems, while seemingly successful, the multipartite, hierarchical nature of the suggested processes may make dissection of the actual selectivities being exploited somewhat hard to interpret. Nonetheless, certain examples allow us to consider interesting concepts of selectivity.

One provocative example is that of a set of Cu-bearing aptamers that are proposed as tumor-selective catalysts for the *in vivo* synthesis of anticancer agents through CuAAC. Creation of a panel with Cu-binding thymine-rich domains joined via a linker domain to an anti-MUC1 aptamer domain drove the apparent formation of two drug “fragments” that circulated freely without reaction until reaching tumor tissue. As a result, in a nude mouse bearing MCF-7 tumors, tumor inhibition was seen with apparently negligible side effects to major organs.^237^ The use of “split” systems is a frequent strategy for *in situ* noncovalent reassembly (fragment complementation) of much larger protein (*e.g.*, enzyme) systems with some success.^238^ Therefore, the use of alternative biomolecule “fragments” (combined through bond formation *in situ*) could be similarly envisaged via functional covalency (perhaps via similar *in situ* metallocatalysis), for example, of biomolecules already known to act as mimics *in vivo* when intact.^208^

**Figure 37:**
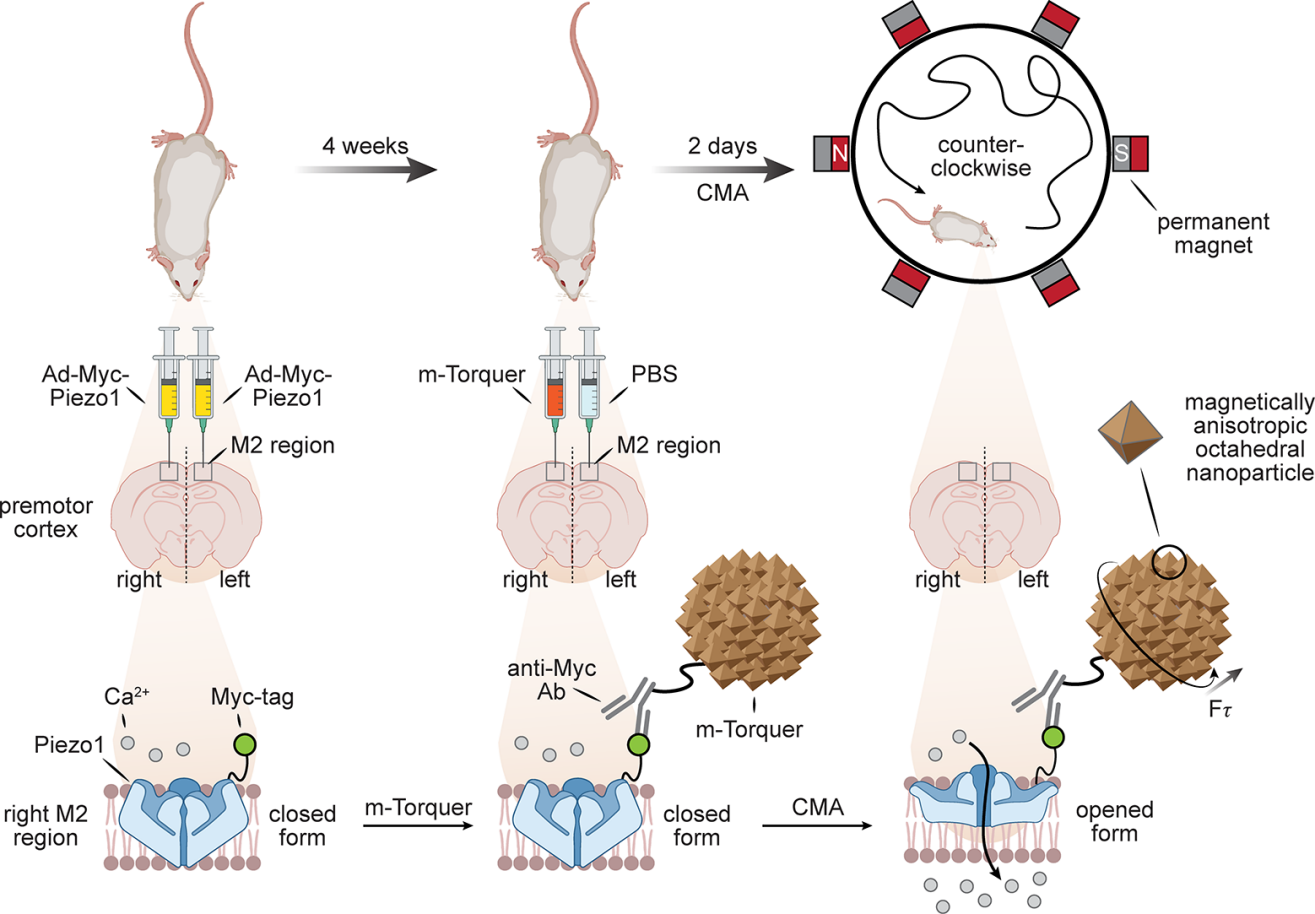
*In vivo* control of a mouse mediated by magnetism. Transgenic mice expressing a Myc-tagged mechanosensitive ion channel in the M2 of the premotor cortex were injected with an anti-Myc mAb conjugated to a magnetic nanoparticle called m-Torquer. Injection into the right-hemisphere only and placement of the mouse into a rotating magnetic field led to more active mice that traveled in a counterclockwise direction.^239^ CMA = circular magnet array.

The reader of this Review will likely have the pragmatic question as to whether examples yet exist in our view of fully worked multilevel selectivity that we seek to set out here as a mode of organismal editing. In short, the answer is “no”. However, there are examples that provide a sense of alternative hierarchical strategies and inventions that may be required. In one,^239^ a route to *in vivo* control in living organisms (albeit not directly through chemistry) presents a thought provoking experiment to inspire LSF of organisms [Figure 37]. Thus, magnetic force was used to induce torque in mechanosensitive neuronal ion channels in living mice through the use of magnetic nanoparticles in a system named m-Torquer. Transgenic mice were generated by expressing the mechanosensitive ion channel Piezo1 conjugated to a Myc tag using an adenoviral system after injection into both the left and right hemispheres of the M2 region of the mouse premotor cortex. After four weeks, anti-Myc-mAb-conjugated m-Torquer nanoparticles were injected to the right hemisphere only, and (after two days of recovery) mice were placed into a rotating magnetic field. Under these conditions, the mice traveled further and faster compared with control mice and, interestingly, circled unilaterally in a counterclockwise direction. This intriguing study presents a unique and fundamentally physical modality for manipulating biology. While the selectivity presented in this example is perhaps somewhat contrived (*i.e.*, injection into the desired hemisphere of the brain mediated through antibody–antigen binding), the striking functionality and control exerted on a living organism are worth noting and could well be logically extended to modes of chemical functionalization in different contexts.

In final brief summary, with selective methodologies now increasingly in place to boldly attempt organismal edits (mediated by bond-formation and/or bond-breaking) and technologies increasingly able to “see” and assess their effects, the “late-stage functionalization” of life as a mode of combined chemistry, biology, physiology, and even medicine is tractable.

## Brief Acronym Glossary (BAG)

Ab: antibody
Aha: azidohomoalanine
Anl: azidonorleucine
BCN: bicyclo[6.1.0]non-4-yne
BrHnL: bromohomonorleucine
CA: carbonic anhydrase
CuAAC: copper-catalyzed azide alkyne
DIFO: difluorocyclooctyne cycloaddition
DBCO: dibenzocyclooctyne
DOTA: 2,2′,2″,2‴-(1,4,7,10-tetraazacyclododecane-1,4,7,10-tetrayl)tetraacetic acid
Hpg: homopropargylglycine
IEDDA: inverse electron demand Diels–Alder
IME: 2-imino-2-methoxyethyl
ITC: isothiocyanate
NASA: *N*-acyl-*N*-alkylsulfonamide
NHS: *N*-hydroxy-succinimide
PyOX: pyridinium oxide
SL: Staudinger ligation
SPAAC: strain-promoted azide–alkyne cycloaddition
SPIEDDA: strain-promoted inverse electron demand Diels–Alder.
TCO: *trans*-cyclooctyne
Tz: tetrazine
uAA: unnatural amino acid
uG: unnatural glycan
uL: unnatural lipid
uR: unnatural residue
